# Development of PCR-based markers and whole-genome selection model for anthracnose resistance in white lupin (*Lupinus albus* L.)

**DOI:** 10.1007/s13353-020-00585-1

**Published:** 2020-09-23

**Authors:** Sandra Rychel-Bielska, Nelson Nazzicari, Piotr Plewiński, Wojciech Bielski, Paolo Annicchiarico, Michał Książkiewicz

**Affiliations:** 1 Department of Genetics, Plant Breeding and Seed Production, Wroclaw University of Environmental and Life Sciences, Plac Grunwaldzki 24A, 50-363 Wrocław, Poland; 2 Institute of Plant Genetics, Polish Academy of Sciences, Strzeszyńska 34, 60-479 Poznań, Poland; 3 CREA-FLC, Council for Agricultural Research and Economics, Research Centre for Fodder Crops and Dairy Production, Viale Piacenza 29, 26900 Lodi, Italy

**Keywords:** White lupin, Marker-assisted selection, Genomic selection, Anthracnose resistance, Quantitative trait

## Abstract

White lupin (*Lupinus albus* L.) is a high-protein grain legume crop, grown since ancient Greece and Rome. Despite long domestication history, its cultivation remains limited, partly because of susceptibility to anthracnose. Only some late-flowering, bitter, low-yielding landraces from Ethiopian mountains displayed resistance to this devastating disease. The resistance is controlled by various genes, thereby complicating the breeding efforts. The objective of this study was developing tools for molecular tracking of Ethiopian resistance genes based on genotyping-by-sequencing (GBS) data, envisaging linkage mapping and genomic selection approaches. Twenty GBS markers from two major quantitative trait loci (QTLs), antr04_1/antr05_1 and antr04_2/antr05_2, were converted to PCR-based markers using assigned transcriptome sequences. Newly developed markers improved mapping resolution around both anthracnose resistance loci, providing more precise QTL estimation. PCR-based screening of diversified domesticated and primitive germplasm revealed the high specificity of two markers for the antr04_1/antr05_1 locus (TP222136 and TP47110) and one for the antr04_2/antr05_2 locus (TP338761), highlighted by simple matching coefficients of 0.96 and 0.89, respectively. Moreover, a genomic selection approach based on GBS data of a recombinant inbred line mapping population was assessed, providing an average predictive ability of 0.56. These tools can be used for preselection of candidate white lupin germplasm for anthracnose resistance assays.

**Electronic supplementary material:**

The online version of this article (10.1007/s13353-020-00585-1) contains supplementary material, which is available to authorized users.

## Introduction

Lupins are valuable crops appreciated as a source of protein for food and feed, as well as plants enhancing mobilization of soil phosphorus, improving soil fertility through symbiotic nitrogen fixation, and increasing economic payback for succeeding crops (Lambers et al. 2013). The seed of modern white lupin (*Lupinus albus* L.) germplasm is characterized by high content of protein, around 38–42% on a dry-weight basis (Papineau and Huyghe 2004); moderate content of oil, around 10–12% (Annicchiarico et al. 2014) with outstanding food quality (Boschin et al. 2007); and low alkaloid content (Lin et al. 2009). Moreover, extracts from different lupin species were revealed to have antimicrobial activity (Confortin et al. 2018; Confortin et al. 2017; Confortin et al. 2019; Erdemoglu et al. 2007; Romeo et al. 2018). During the domestication process, germplasm resources with dwarf architecture determinate growth habit and higher cold tolerance have also been selected (Harzic et al. 1995; Huyghe and Papineau 1990; Julier et al. 1993). Moreover, the assay of 121 entries representing the global white lupin germplasm revealed that potentially high-yielding landraces are available for exploitation in breeding programs (Annicchiarico et al. 2010). However, worldwide attempts of white lupin improvement have been hampered by high susceptibility to anthracnose, caused by the pathogenic fungus, *Colletotrichum lupini* (Bondar) Nirenberg, Feiler & Hagedorn (Nirenberg et al. 2002). Typical symptoms were observed as early as in 1912 in Brazil, but the underlying fungus was identified three decades later (Weimer 1943). Early screening of the resistance revealed some level of resistance in *L. angustifolius* and *L. luteus* germplasm and high susceptibility of all *L. albus* accessions tested (Weimer 1952). The appearance of the disease on white lupins in France (1982) and Ukraine (1983) has challenged dramatically the European white lupin breeders (Gondran et al. 1996). Soon afterwards, anthracnose appeared worldwide in nearly all regions where white lupins are cultivated, including major producers such as Australia, Poland, and Germany (Frencel 1998; Frencel et al. 1997; Sweetingham et al. 1995; Talhinhas et al. 2016). Anthracnose susceptibility is a more important issue in white lupin than in the narrow-leafed lupin, because the latter has several independent sources of resistance already present in improved germplasm (Boersma et al. 2005; Fischer et al. 2015; Yang et al. 2004; Yang et al. 2008). This disease proved to be a critical obstacle for the agronomic improvement of white lupin, as the only source of resistance, located in a mountainous region of Ethiopia, has been identified hitherto (Phan et al. 2007) in the form of several accessions collected in one district (Adhikari et al. 2009). Recent studies revealed that Ethiopian germplasm landraces carry rare alleles and are relatively uniform genetically (Atnaf et al. 2017; Raman et al. 2014).

The lack of modern breeding tools hampered the rate of white lupin genetic improvement. Breeders have spent more than two decades to harness Ethiopian anthracnose resistance alleles with very limited success rate: only a few cultivars showing an incremental improvement of anthracnose resistance were bred (Adhikari et al. 2009; Adhikari et al. 2013; Jacob et al. 2017; Talhinhas et al. 2016). Molecular genomic resources of white lupin include two mapping populations with associated low-density linkage maps (Croxford et al. 2008; Phan et al. 2007; Vipin et al. 2013) and a draft transcriptome assembly (O'Rourke et al. 2013). Some sequence tagged site (STS) markers linked to low alkaloid content (PauperM1) and anthracnose resistance (WANR1, WANR2 and WANR3) (Lin et al. 2009; Yang et al. 2010) were developed, but the recombination rate between these markers and corresponding trait loci was too high for their implementation in marker-assisted selection. The reference recombinant inbred line (RIL) mapping population developed from the cross Kiev Mutant (Ukraine) × P27174 (Ethiopia) segregates for many agronomic traits, including also the resistance to anthracnose inherited from Ethiopian parents (Phan et al. 2007). Trait loci have been localized on the linkage map (Cowley et al. 2014; Phan et al. 2007; Vipin et al. 2013), but low marker density, namely one marker per 10.8 cM (Phan et al. 2007) and one per 4.6 cM (Vipin et al. 2013), impeded quantitative trait loci (QTL) mapping attempts. Recently, genotyping-by-sequencing (GBS) data were exploited to develop a new high-density consensus linkage map of the species based on new, transcriptome-anchored markers (Książkiewicz et al. 2017). Mapping of white lupin QTLs revealed polygenic control of anthracnose resistance and provided a bunch of allele-sequenced markers tagging these QTLs, opening new possibilities for development of tools for molecular tracking of anthracnose resistance. For traits controlled by various genes, an alternative approach to marker-assisted selection is represented by genomic selection, by which major and minor gene effects are taken into account by a statistical model for breeding value prediction that is constructed from the joint analysis of phenotyping and genotyping data of a germplasm sample that represents well the target genetic base (Heffner et al. 2009; Meuwissen et al. 2001). The first investigations of genomic selection for white lupin revealed high ability to predict grain yield in contrasting European environments (Annicchiarico et al. 2019) and several morphophysiological and agronomic traits in Northern Italy (Annicchiarico et al. 2020).

The present paper aims to exploit the molecular information generated by markers (Książkiewicz et al. 2017) to develop PCR-based array tagging two major white lupin anthracnose resistance QTLs, ultimately facilitating preselection of germplasm carrying candidate Ethiopian alleles of anthracnose resistance genes. As an additional objective, we assessed preliminarily the applicability of a genomic selection approach for prediction of anthracnose resistance based on GBS data of a mapping population.

## Materials and methods

### Transformation of GBS polymorphisms to PCR-based markers

To select reference transcript sequences for primer design, GBS marker sequences from QTL loci (Książkiewicz et al. 2017) were aligned to assembled transcriptomes of Kiev and P27174 lines as well as to reference white lupin gene index LAGI01 (O'Rourke et al. 2013) by BLAST (Altschul et al. 1990) implemented in the Geneious software (Kearse et al. 2012) under an *e*-value cut-off of 1e−10. Selected transcripts were then aligned to the genome sequence of the narrow-leafed lupin (Hane et al. 2017) under an *e*-value cut-off of 1e−15, extracting matching regions with context size of 5000 nt. To find exon/intron boundaries, extracted narrow-leafed lupin genome regions were assembled together with white lupin GBS and transcript sequences into contigs using progressive Mauve algorithm (Darling et al. 2004) assuming genome collinearity. Mauve alignments consisting of corresponding markers, *L. albus* Kiev, P27174 and LAGI01 transcripts and *L. angustifolius* scaffolds were screened for the presence of polymorphic loci. The primers flanking these loci were designed using Primer3Plus (Untergasser et al. 2007) and *L. albus* cDNA sequences as templates. DNA was isolated from *L. albus* Kiev and P27174 using DNeasy Plant Mini Kit (Qiagen, Hilden, Germany) and ethyl alcohol (96%, < 0.1% methanol, Avantor Performance Materials, Gliwice, Poland). Amplification was performed using GoTaq® Flexi DNA Polymerase (Promega, Madison, USA) and Labcycler Gradient thermal cycler (Sensoquest, Göttingen, Germany). Ninety-six-well PCR plates (4titude, Wotton, Surrey, UK) and standard tips (Neptune Scientific, San Diego, USA) were used. Amplicons were purified directly from the post-reaction mixtures (QIAquick PCR Purification Kit; Qiagen) and sequenced (ABI PRISM 3130 XL Genetic Analyzer; Applied Biosystems, Hitachi) in the Laboratory of Molecular Biology Techniques, Faculty of Biology, Adam Mickiewicz University (Poznan, Poland). Nucleotide substitution polymorphisms were resolved by the cleaved amplified polymorphic sequence (CAPS) (Konieczny and Ausubel 1993) or derived CAPS (dCAPS) (Neff et al. 1998) approaches. Restriction sites and dCAPS primers were identified using dCAPS Finder 2.0 (Neff et al. 2002) and SNP2CAPS (Thiel et al. 2004). Restriction enzymes were supplied by New England Biolabs (Ipswich, USA) and Thermo Fisher Scientific (Waltham, USA), depending on price and availability. Restriction products were separated by agarose gel electrophoresis (Wide Range Agarose, Serva, Heidelberg, Germany) with the agarose concentration (1–3%) adjusted to follow the size of the expected digestion products. Standard Tris-Borate-EDTA buffer was exploited (Serva). Electronic expandable multichannel pipette (Matrix, Thermo Fisher Scientific) was used for transfers of samples between PCR plates and gels. Data on developed molecular markers, including primer sequences, annealing temperature, nucleotide sequence identity, and polymorphic loci are provided in Tables 1 and 2 as well as in the Supplementary Table S1.

**Table 1:** List of developed markers for antr04_1/antr05_1 QTL, with primer sequences, PCR amplification temperature, validated enzymes, and restriction product sizes

Name	Primers	PCR °C	Enzyme	Products Kiev Mutant (length in bp)	Products P27174 (length in bp)
WANR1F^a^ WANR1R	GAGTCACTTAGAAATAAAGG GATCCATGAAGATACATTG	51	–	~ 190	~ 180, ~ 220
TP23903F TP23903R	CAGCAATATTGAGAGCAACCAA TGTATTATATGGCTTTGATTGTGTC	56	*Bse*GI	49, 15	64
TP229924F TP229924R	CACACTTGCACTTAATGGTTATATGCACG AAAATCCCCACCCAAATGGC	60	*Rsa*I	54, 30	84
TP272531F TP272531R	TCATTGTCATAGATAATTGCAGCT GAGCCACTTGATGCATGTACA	60	*Hpy*F3I	112, 49	161
TP222136F TP222136R	CTTCACCCAGTCTCTATCTGCAC AATGAGCATGCTTAATCTTGTTGCA	63	*Cvi*KI-1	168, 27, 15	183, 27
TP47110F TP47110R	TTAGCTGGTTTAATGTGGTGGCACTCA AAAGAGCAAACCAAGCCTATCT	60	*Hpy*F3I	42, 24	66
TP446132F TP446132R	CAAAAGCAGGTTGATGTGAATTCT CAGCTGCTGGTTTTCGTTGAAA	60	*Taa*I	240	130, 110
TP291372F TP291372R	GCGAATGCTTTCTCTTGTTCTTG ACATACCCACTGAGATCAAGCA	60	*Mun*I	54, 51	105
TP237794F TP237794R	GCTCATAACTTAGCTCCTTTTCCCT AAATGAGGCACCTACATCAAGAACTTA	64	*Mse*I	39, 27	66
TP38227F TP38227R	AATGAAACGAACTCTTCTTGCAGC TGGCTTTCACTTCTCAGCTATTTG	60	*Mwo*I	76, 29	105
TP88533F TP88533R	CTGAACCCAGCATCAGTGTT ATCAAATAGCTGAGAAGTGAAAGCC	60	*Nla*III	99	55, 44

^a^As previously published (Yang et al. 2010)

**Table 2:** List of developed markers for antr04_2/antr05_2 QTL, with primer sequences, PCR amplification temperature, validated enzymes, and restriction product sizes

Name	Primers	PCR °C	Enzyme	Products Kiev Mutant (length in bp)	Products P27174 (length in bp)
WANR3F^a^ WANR3R	TTAAGCCAAGATTCTACTTAG ACTAGCACTTGTGTGTGTGTGTG	51	–	160	141
TP149038F TP149038R	CCTCAGCATGTCCAAGTCGAA TTCACTTTGCCAGCCTTTTCTT	60	*Mun*I	100	65, 35
TP416765F TP416765R	CACACTGGTCATGCTTCCTTCAA GCGGCTGGATGTGGAGGA	60	*Bse*DI	54, 38	92
TP3712F TP3712R	TCAACAACACAAATCAATGCAACA CCGAAGCAGAACCACCAAAAT	60	*Cvi*KI-1	171, 29	123, 48, 29
TP364001F TP364001R	CCGAAGCAGAACCACCAAAAT CACAAATCAATGCAACAATCACA	56	*Cvi*KI-1	116, 48, 29	164, 29
TP37593F TP37593R	CAGCAGCACCCATTTGGAAAG AGTAACGTCATCCACTATAGAAAA	56	*Mbo*II	68, 51	119
TP26007F TP26007R	TCTGGTTCACCGGTTTATCTCCGACTGAC GGTAGCCACTGATTTCGGTTC	60	*Nla*III	107	75, 32
TP106254F TP106254R	GTCCCGAAGTATATTTATCAGAAGG GGGATCACTCATCTATCCAAT	56	*Tas*I	52, 50, 16	68, 50
TP338761F TP338761R	TCCTTGAGAGAATCCAAGCTGC CTACAATGCACACGAGATTGCC	60	*Sch*I	83, 28	64, 47
TP440375F TP440375R	TACCCACATTTATGTAAACCTTTGACTTT AAAAGGCTGAGTTAGACACACAC	60	*Dra*I	70, 29	99
TP93026F TP93026R	CAGTGGTTGCTGGCTCTTCCA CGATCATCCACGCCACTATGC	56	*Nla*III	33, 23	56

^a^As previously published (Yang et al. 2010)

### Linkage mapping

Genetic mapping was performed using the reference Kiev x P27174 F_8_ recombinant inbred line (RIL) population (*n* = 196) delivered by the Department of Agriculture and Food Western Australia. This population was derived from a cross between the anthracnose-resistant Ethiopian landrace P27174 and susceptible Ukrainian line Kiev Mutant (Phan et al. 2007). Kiev Mutant-like scores were assigned as *b*, P27174-like scores as *a*, and heterozygotes as *h*.

Chi-square (*χ*^2^) values for Mendelian segregation in F_8_ RILs were estimated using the following expected segregation ratios: 0.4961 (Kiev Mutant), 0.4961 (P27174), and 0.0078 (heterozygote). The calculation of probability was based on *χ*^2^ and 2 degrees of freedom. *L. albus* marker segregation files (Książkiewicz et al. 2017; Phan et al. 2007; Vipin et al. 2013), together with those developed in this study, were imported to JoinMap 4.1 (Stam 1993). Multipoint mapping was performed after grouping under independence LOD of 11.0 (parameters in the Supplementary Table S2). Linkage group optimization was performed according to the procedure described by Książkiewicz et al. 2017.

The Pearson product-moment correlation coefficient between averaged anthracnose disease resistance scores (Książkiewicz et al. 2017) and marker allelic phases for the set of 191 mapping population lines was calculated in Excel. The *p* value (both one-tailed and two-tailed probability) of a Pearson correlation coefficient was calculated using the *p* value calculator for correlation coefficients (http://www.danielsoper.com/statcalc).

#### Anthracnose resistance QTL mapping

Data on anthracnose resistance scores (Książkiewicz et al. 2017) and an updated linkage map from this study were used to re-draw QTL loci. As the study was aiming to develop PCR-based markers for preselection of germplasm carrying published candidate anthracnose resistance loci, we did not perform new phenotyping of RIL population. Thus, we used average values (trait antr_avg) across two years of anthracnose resistance screening in Perth, Australia, which encompassed three independent experiments per year (trait antr04, *n* = 151; trait antr05, *n* = 191) (Książkiewicz et al. 2017; Phan et al. 2007). Interval mapping (van Ooijen 1992) was performed using MapQTL 6 (Kyazma, Wageningen, Nederlands) (Supplementary Table S3). The LOD threshold of 3.5 was used for QTL determination. QTLs were located at positions with the highest LOD values. Composite interval mapping was performed in Windows QTL Cartographer V2.5 (North Carolina State University, Raleigh, USA) using 20 background control markers window size 10 cM and walk speed 0.5 cM to exactly follow the approach used during the reference study (Książkiewicz et al. 2017). Linkage groups and LOD graphs were drawn in MapChart (Voorrips 2002). Marker sequences developed in this study (LC416306–LC416345) were aligned to the *L. albus* genome assembly (Hufnagel et al. 2020), using custom BLAST database (*e*-value 1e−20, 1 best hit) implemented in Geneious, to find genome regions collinear to QTL loci conferring anthracnose resistance. Genes localized in these regions were screened for the presence of typical domains using the Disease Resistance Analysis and Gene Orthology (DRAGO 2) tool at the Plant Resistance Genes database (PRGdb) (Osuna-Cruz et al. 2018).

### Assay of marker polymorphism in diversified germplasm

The set of 107 *L. albus* lines derived from the European Lupin Gene Resources Database maintained by Poznań Plant Breeders Ltd. station located in Wiatrowo was used: 51 primitive populations, 30 landraces, 16 cultivars, 7 cross derivatives, and 3 mutants. These lines originated from 22 countries (Supplementary Table S4). Markers were scored using the same methods as those applied for the RIL population. For binary data similarity analysis, Kiev Mutant-like scores were assigned as 0, P27174-like scores as 1, heterozygotes as 1, and additional alleles as 0. Simple matching (Sokal and Michener 1958) and Rogers-Tanimoto (Rogers and Tanimoto 1960) coefficients were calculated using a Binary Similarity Calculator http://www.minerazzi.com/tools/similarity/binary-similarity-calculator.php.

### Genomic selection model

A genomic selection model was built to investigate the possibility of predicting anthracnose resistance scores derived from the RIL population. The set of GBS-derived single nucleotide polymorphism (SNP) markers, after validation via chi-square test, was filtered for growing levels of missing rate per marker (10%, 20%, 30%, 40%, and 50%), resulting in five different genotype matrices with a growing number of markers (631, 1859, 2430, 2833, and 3230, respectively). Following Nazzicari et al. (2016), the remaining missing data-points were imputed using Random Forest Imputation by the R package *missForest* (Stekhoven and Bühlmann 2012) with parameters ntree = 100, maxiter = 10, and parallelize = variables. The target traits were the two sets of anthracnose disease resistance scores issued from each year of evaluation (traits antr04 and antr05) and the average score across years (trait antr_avg).

For genome-wide predictions, we applied the ridge regression BLUP model (Searle et al. 2008), which displayed comparatively high predictive ability in prior studies on other legume species such as pea (Annicchiarico et al. 2017a), white lupin (Annicchiarico et al. 2019), and alfalfa (Annicchiarico et al. 2015). Ridge regression BLUP analysis was performed through the R software package rrBLUP (Endelman 2011), assessing the model predictive ability as Pearson’s correlation between true and predicted scores. In particular, the mixed model:

$$ y= Zu+e $$

with *y* being the phenotypes, *Z* being the genotype matrix, and *u* a vector of random effects with variance *σ*^2^_*u*_ was solved in a maximum likelihood (ML/REML) framework using the function “mixed.solve” from rrBLUP package. With this approach, the (equivalent in ML of the) ridge parameters is estimated via *λ = σ*^2^_*e*_/*σ*^2^_*u*_ and thus it can be computed directly from the data without variance component estimation.

The model was trained in a tenfold cross-validation schema, and training was repeated 50 times for each trait and then averaged to ensure numerical stability, using the R package GROAN (Nazzicari and Biscarini 2017).

## Results

### Twenty PCR-based markers were developed for two major anthracnose resistance QTLs

The region from 0.00 to 4.74 cM on the linkage group ALB02, which carried the antr04_1/antr05_1 anthracnose resistance QTL (explaining approximately 25–28% of phenotypic variance) (Książkiewicz et al. 2017; Phan et al. 2007) and contained 22 sequence-defined markers developed by GBS, was selected. Perfectly matching transcriptome sequences were assigned for 14 markers (64%) using available datasets (Książkiewicz et al. 2017; O'Rourke et al. 2013). Eleven GBS markers were selected for transformation to PCR-based markers (one directly and ten using complementary transcriptome sequences), based on the position on the linkage map and availability of affordably priced restriction enzymes. The summary of this transformation is included in Supplementary Table S5. Comparative mapping to *L. albus* transcriptome and *L. angustifolius* genome sequences (Hane et al. 2017) provided coordinates for intron/exon boundaries for ten markers. The list of *L. albus* transcriptome contigs anchored to GBS sequences is provided in Supplementary Table S6, whereas the coordinates of corresponding *L. angustifolius* genome regions are provided in Supplementary Table S7. The position of markers confirmed the highly conserved collinearity between the regions of *L. albus* linkage group ALB02 and *L. angustifolius* chromosome NLL-20. The WANR1 marker already implemented in marker-assisted selection of antr04_1/antr05_1 QTL in Australian breeding programs (Yang et al. 2010) was also included in the analysis. PCR amplicons were obtained for all primer pair combinations. Restriction enzyme cleavage of these amplicons yielded expected products for all markers except TP254603, which revealed additional unspecific amplification. The list of developed markers with primer sequences, PCR amplification temperature, validated enzymes, and restriction product sizes is given in Table 1.

A second region selected for marker development was that from 115.99 to 128.75 cM on the linkage group ALB04, which carried antr04_2/antr05_2 anthracnose resistance QTL (explaining approximately 15–23% of phenotypic variance) (Książkiewicz et al. 2017; Phan et al. 2007) and included 23 GBS markers. Twelve GBS sequences were chosen for marker development (Supplementary Table S5). Alignment to *L. albus* transcriptome and *L. angustifolius* genome sequences provided matching sequences for ten and twelve GBS sequences, respectively, and provided novel evidence for collinearity between the regions of *L. albus* linkage group ALB04 and *L. angustifolius* chromosome NLL-02 (Supplementary Table S6 and S7). WANR3 marker used for molecular selection of antr04_2/antr05_2 allele in Australian breeding programs (Yang et al. 2010) was also included in the analysis. PCR amplicons were obtained for 11 primer pairs (85%). Restriction enzyme cleavage confirmed the presence of expected polymorphic loci in all amplicons (Table 2).

### PCR-based markers refined mapping resolution at both QTL loci

**Fig. 1:**
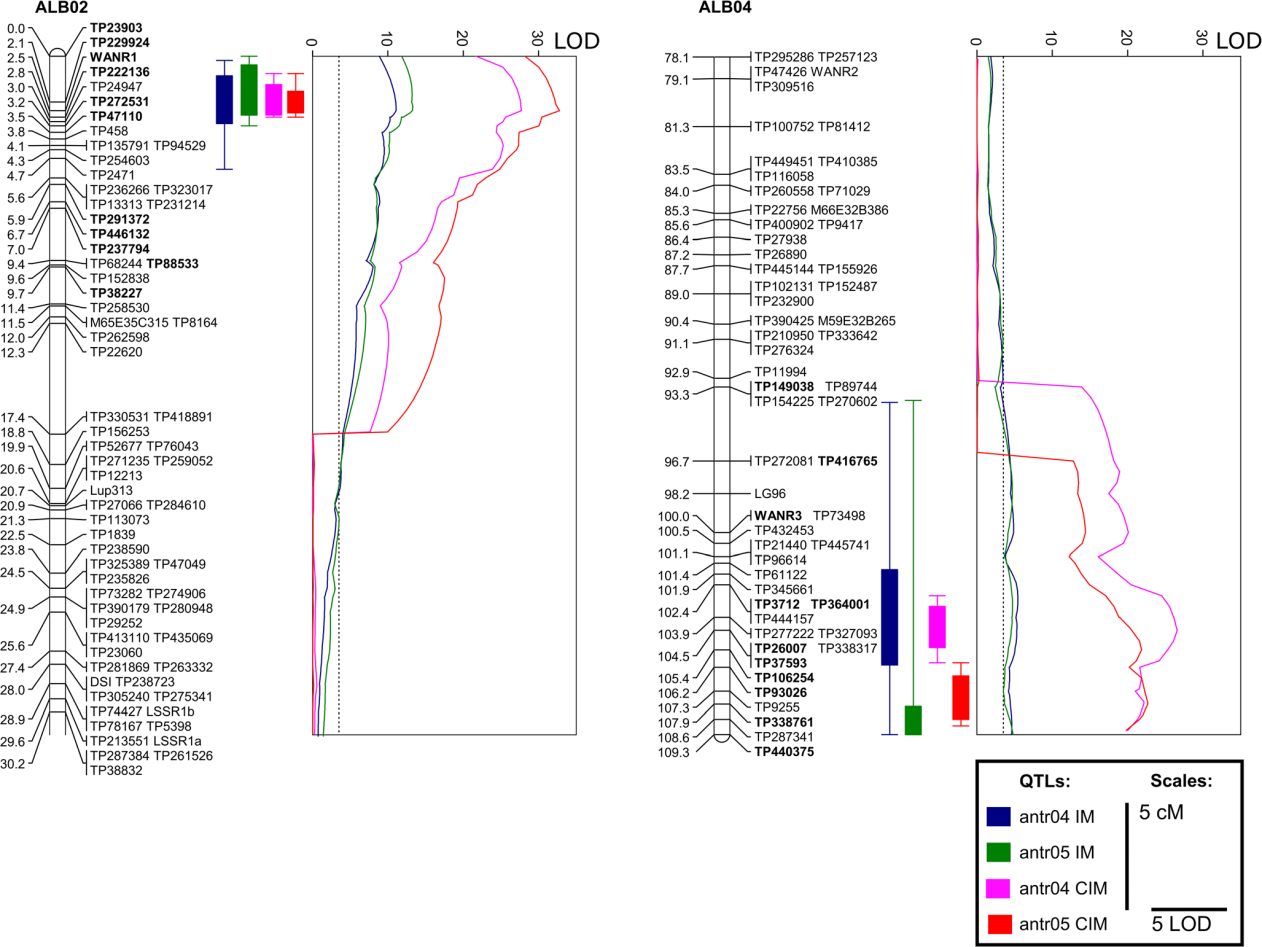
Major QTLs for anthracnose resistance in white lupin. Linear plots show LOD values (threshold 3.5); rectangles, LOD-based QTL ranges (LOD 2.0 and 1.0 below the maximum value), whereas bar graphs visualize corresponding linkage group fragments. Names of markers included into PCR-based assay are bold faced. Colors correspond to QTL assays: interval mapping, IM, blue (antr04, the first year) and green (antr05, the second year); composite interval mapping, CIM, pink (antr04) and red (antr05). Linkage groups and LOD graphs are drawn to scale

The segregation pattern of 22 markers in the white lupin RIL mapping population was resolved. Newly developed GBS-derived and previously published simple sequence repeat (SSR)-derived PCR markers revealed high amplification stability, providing on average 98.4% (antr04_1/antr05_1) and 98.5% (antr04_2/antr05_2) reads for the RIL population. Taking into consideration that GBS markers had on average 71.4% RIL data for antr04_1/antr05_1 and 74.4% for antr04_2/antr05_2, this approach improved segregation data by 37.8% and 32.4%, respectively (Supplementary Table S8 and S9). Thus, it contributed to linkage mapping refinement and increased map resolution as visualized by smaller blocks of co-segregating markers in both regions (Fig. 1). The order of markers was highly reproducible, as highlighted by low SD values of relative marker positions calculated for ten mapping runs: from 0.0 to 0.6 for antr04_1/antr05_1 and from 0.0 to 2.2 for antr04_2/antr05_2 loci (Supplementary Table S9). High fidelity of these markers was reflected by high LOD values of linkage to adjacent markers, ranging from 22.3 to 55.1 in antr04_1/antr05_1 and from 18.6 to 56.0 in antr04_2/antr05_2.

The updated linkage map was subjected to QTL mapping using phenotype observations from previous studies (Książkiewicz et al. 2017). The presence of two major anthracnose resistance QTLs in linkage groups ALB02 and ALB04, resolving about 45–50% of observed phenotypic variance, was confirmed (Table 3, Fig. 1). Linkage map improvement contributed to higher LOD values compared to the earlier study (Książkiewicz et al. 2017), indicating strengthened statistical significance of QTL mapping results. LOD values for antr04_1/antr05_1 locus were 13.3 in this study vs 10.3 (the most recent linkage map) in interval mapping (MapQTL), and 32.8 vs 22.7 in composite interval mapping (WinQTL Cartographer). The same result was observed for antr04_2/antr05_2, yielding LOD values 5.4 vs 5.2 in interval mapping and 26.6 vs 14.7 in composite interval mapping. The position of the LOD peak for the major resistance QTL (antr04_1/antr05_1) was almost identical for both experiments and methods, fitting within the range of 0.2 cM. The position of the LOD peak for the second QTL (antr04_2/antr05_2) covered the range of 6.2 cM.

**Table 3:** Two major anthracnose resistance QTLs detected in a recombinant inbred line population of white lupin. PVE - proportion of phenotypic variance explained by QTL

QTL	LG	Interval mapping (MapQTL6)				Composite interval mapping (WinQTL Cartographer)			
		Locus (cM)	LOD	Additive effect	PVE	Locus (cM)	LOD	Additive effect	PVE
antr04_1	ALB02	2.5	11.1	− 0.58	28.8	2.5	27.7	− 0.61	25.1
antr05_1	ALB02	2.3	13.3	− 0.46	27.7	2.5	32.8	− 0.45	23.9
antr04_2	ALB04	103.1	5.4	− 0.42	15.3	104.5	26.6	− 0.54	23.1
antr05_2	ALB04	109.3	4.8	− 0.28	11.0	107.9	22.7	− 0.34	14.6

### Three markers revealed applicability for Ethiopian allele selection

Pearson correlation between the observed anthracnose resistance phenotype and marker genotype in the RIL population was in the range of 0.49–0.58 for antr04_1/antr05_1 and 0.28–0.39 for antr04_2/antr05_2. The significance (both one-tailed and two-tailed probability) of the correlation coefficients, given the correlation values and the sample size (190 RILs with anthracnose resistance scores), was very high: *p* values were ~ 0.0 for antr04_1/antr05_1 and below 0.00009 for antr04_2/antr05_2.

**Fig. 2:**
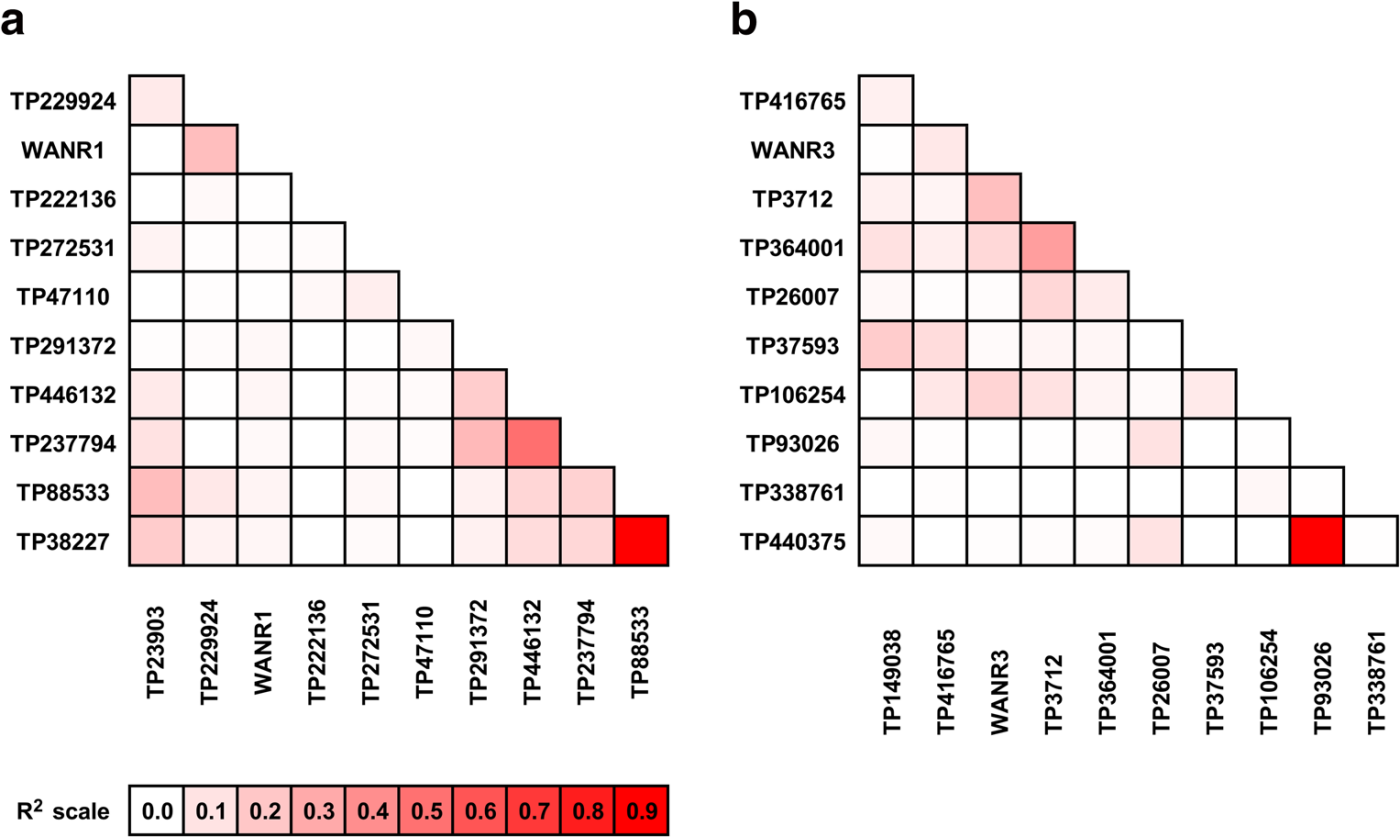
Linkage disequilibrium pattern observed for PCR-based markers from ALB02 (**a**) and ALB04 (**b**) linkage groups. The set of 107 white lupin lines originating from 22 countries (81 primitive populations and landraces and 26 domesticated) was used to estimate *R*^2^ values

Significant linkage disequilibrium decay was observed in both anthracnose resistance QTL regions in a set of 107 white lupin core collection lines (Fig. 2, Supplementary Table S10). As the resistance allele originated from one mountainous region of Ethiopia (Adhikari et al. 2013; Raman et al. 2014), it is uncommon to have it in the germplasm which has not been crossed with any Ethiopian line. Therefore, we expected to have “susceptible” marker scores for all European, African, and Middle East lines except the Ethiopian parent P27174 (distribution ratio like 106 vs 1). Simple matching coefficients were calculated, to compare the marker and expected phenotype. These values ranged from 0.25 to 0.96 for antr04_1/antr05_1 and 0.33–0.89 for antr04_2/antr05_2, indicating high similarity of marker pattern and hypothetical possession of resistant alleles.

We calculated values of the Rogers-Tanimoto (RT) coefficient, to address the putative applicability of markers in the selection of germplasm for further disease resistance assays. This coefficient is a variant of the simple matching coefficient that gives double weight to mismatching variables, thereby resulting more convenient for the analysis of false-positive scores. We found high RT values, with maxima of 0.93 in antr04_1/antr05_1 and 0.80 in antr04_2/antr05_2. This was a considerable improvement over the previous markers, highlighted by RT values as low as 0.59 for antr04_1/antr05_1 (WANR1) and 0.49 for antr04_2/antr05_2 (WANR3). Two newly developed markers for antr04_1/antr05_1 (TP222136 and TP47110) and one for antr04_2/antr05_2 (TP338761) were revealed to be applicable for selection of Ethiopian alleles with > 90% confidence (Table 4, Supplementary Figure S1).

**Table 4:** Results of PCR marker validation: distance to QTL peak on the linkage map (cM), correlation values between anthracnose resistance phenotype and marker genotype in RIL population, and simple matching (SM) and Rogers-Tanimoto (RT) coefficient values, in lines of a white lupin core collection

Name	Distance to QTL (cM)	Correlation coefficient for RIL population	SM value for germplasm collection	RT value for germplasm collection	Applicability for marker-assisted selection
TP23903	− 2.50	0.55	0.54	0.37	−
TP229924	− 0.36	0.58	0.78	0.63	−
WANR1	− 0.02	0.58	0.74	0.59	−
TP222136	+ 0.33	0.56	0.96	0.93	+
TP272531	+ 0.67	0.55	0.83	0.71	−
TP47110	+ 1.02	0.53	0.96	0.93	+
TP291372	+ 3.39	0.49	0.34	0.20	−
TP446132	+ 4.19	0.53	0.25	0.14	−
TP237794	+ 4.45	0.51	0.25	0.14	−
TP88533	+ 6.96	0.48	0.52	0.35	−
TP38227	+ 7.20	0.51	0.49	0.32	−
TP149038	− 12.89	0.28	0.53	0.36	−
TP416765	− 9.50	0.36	0.56	0.39	−
WANR3	− 6.20	0.36	0.65	0.49	−
TP3712	− 3.83	0.38	0.58	0.41	−
TP364001	− 3.83	0.39	0.33	0.20	−
TP37593	− 1.68	0.38	0.65	0.49	−
TP26007	− 1.68	0.38	0.64	0.48	−
TP106254	− 0.84	0.36	0.65	0.49	−
TP93026	0.00	0.35	0.64	0.48	−
TP338761	+ 1.89	0.34	0.89	0.80	+
TP440375	+ 3.10	0.37	0.66	0.50	−

### Genome regions carrying anthracnose resistance QTLs encode candidate disease resistance genes

Alignment of marker sequences developed in this study (LC416306–LC416345) to the *L. albus* genome assembly (Hufnagel et al. 2020) revealed high collinearity between the linkage map and genome assembly in both QTLs. The list of genes identified in the regions of white lupin genome carrying anthracnose resistance loci is provided in the Supplementary Table S12. Several candidate genes were identified, namely Lalb_Chr02g0142231 (putative protein ENHANCED DISEASE RESISTANCE 2, EDR2), Lalb_Chr02g0141611 (putative protein kinase RLK-Pelle-LRR-XI-1 family), and Lalb_Chr02g0141701 (putative transferase, protein kinase RLK-Pelle-LRR-II family) for the antr04_1/antr05_1 locus and Lalb_Chr04g0264801 (putative protein kinase RLK-Pelle-RLCK-IXb family) for the antr04_2/antr05_2 locus. Screening of coding sequences using the Plant Resistance Genes database revealed the presence of kinase, coiled-coil (CC), leucine-rich repeat (LRR), nucleotide binding site (NBS), Toll/interleukin-1 receptor (TIR), and transmembrane (TM) domains (Supplementary Table S13).

### Genomic selection displayed moderately high ability to predict anthracnose tolerance

**Fig. 3:**
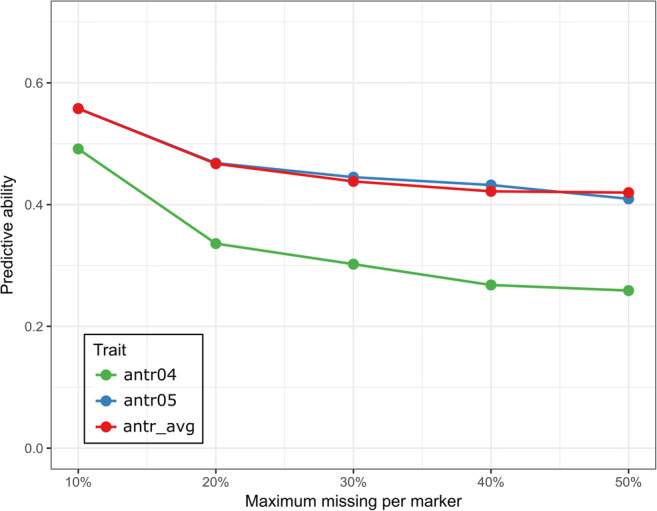
Predictive ability of ridge regression BLUP models as measured by Pearson’s correlation between true and predicted values as a function of the maximum allowed missing rate for single SNP markers, for three anthracnose disease resistance scores from the first year (antr04), the second year (antr05), and mean from both years (antr_avg). Values are derived through 10-fold cross-validations and averaged over 50 repetitions

Average predictive abilities of whole-genome regression models for the three traits (antr04, antr05, and antr_avg) are reported in Fig. 3. The best predictive ability values were found for antr05 and antr_avg, which showed similar values. The trait antr04 displayed lower predictive ability values, probably because of the lower number of available samples (only 151, compared to 191 available for antr05). When one of the two scores was missing, the average resistance score (antr_avg) was based only on data of the other score (Książkiewicz et al. 2017). The effect of filtering on maximum allowed missing rate for markers exhibited a clear pattern of better predictions with low missing rates. The absolute best performances were achieved for 10% maximum missing rate (implying 631 markers), which resulted in predictive abilities of 0.491, 0.558, and 0.557 for antr04, antr05, and antr_avg, respectively. This can be linked to the effective total missing rate pre-imputation. For the filtering thresholds of 10%, 20%, 30%, 40%, and 50%, the utilized data set resulted in missing rates of 1.8%, 4.8%, 7.2%, 9.8%, and 13.2%, respectively.

## Discussion

### Ethiopian sources of anthracnose resistance arise from non-domesticated primitive lines

Following the anthracnose appearance in 1996, all *L. albus* lines from the Lupin Genetic Collection at the Department of Agriculture and Food Western Australia were phenotyped for anthracnose resistance. From more than 8 hundred accessions tested, originating from 21 countries and four continents, all breeding materials (416 lines) and 97% of primitive populations and landraces were found to be very susceptible to anthracnose (Adhikari et al. 2009). Despite testing of a large seed collection, a significant and reproducible level of resistance was found only in several Ethiopian landrace accessions, P27172, P27174, P27175, P27178, P28512, P28523, and P28538 (Adhikari et al. 2009). The three most resistant lines (P27172, P27174, P27178) were collected in one district, Debre Markos, at altitudes around 2000 m. Genotyping of 94 white lupin accessions with PCR-based SSR and microarray-based Diversity Array Technology markers revealed that Ethiopian accessions formed a separate clade, indicating their close genetic relation and distinctiveness from other germplasm (Raman et al. 2014). Assay based on the analysis of agronomical and phenological traits revealed relatively high similarity of Ethiopian white lupin germplasm grouped into several clusters showing significant genetic distance from the German accession used as an outgroup (Atnaf et al. 2015). Recent SSR-based screening of 212 Ethiopian white lupin landraces confirmed low population differentiation among four major white lupin collection areas in the country (Atnaf et al. 2017). These observations have substantial consequences for breeders, who shall apparently deal with the only genetic source of white lupin anthracnose resistance worldwide, buried in landrace germplasm locally adapted to mountain Ethiopian climatic conditions and carrying numerous undesired traits such as high alkaloid content, late flowering, vernalization responsiveness, and low yield (Adhikari et al. 2009; Kroc et al. 2017; Lin et al. 2009; Phan et al. 2007). Indeed, Ethiopian germplasm displayed poor adaptation to European environments in a multi-environment evaluation (Annicchiarico et al. 2010). The line P27174 as a well-recognized anthracnose resistance donor was crossed with the very susceptible Kiev Mutant to generate an advanced recombinant inbreed line population for mapping studies (Phan et al. 2007). Anthracnose phenotyping in independent experiments, followed by marker development and linkage mapping, provided clear evidence for a quantitative pattern of resistance, indicating the involvement of several unrelated heritable factors (Adhikari et al. 2009; Książkiewicz et al. 2017; Phan et al. 2007; Vipin et al. 2013; Yang et al. 2010). White lupin breeding for anthracnose resistance has been hampered so far by a lack of molecular markers to track resistant alleles. There were only few markers linked to white lupin agronomic traits published hitherto, one for the low-alkaloid *pauper* locus and three for two anthracnose resistance loci, and the usefulness of these markers was limited by non-negligible ratios of false-positive scores in diversified germplasm (Lin et al. 2009; Yang et al. 2010). White lupin anthracnose resistance donors are late flowering. Both traits are under polygenic control, which makes breeding attempts much more challenging than in the narrow-leafed lupin. Combining anthracnose resistance with early flowering by traditional breeding has been fairly unsuccessful over two decades, and the hypothesis of a linkage between these traits was put forward (Adhikari et al. 2009). This hypothesis was recently confirmed, as the major anthracnose resistance QTL (antr04_1/antr05_1) and one of the major early flowering QTLs (nonv05_1/nonv15_1/nonv16_1) were located in the same linkage group (Książkiewicz et al. 2017; Rychel et al. 2019). Moreover, one of the few minor early flowering QTLs was found just several centimorgans away from the antr04_1/antr05_1 locus.

### Availability of marker-assisted selection for lupin breeders

The white lupin PCR-based marker toolbox contains several markers developed for candidate genes (*FLOWERING LOCUS T*, *GIGANTEA*, *SEPALLATA*, and *FRIGIDA*) conferring QTLs of flowering time (Rychel et al. 2019), a pair of markers tagging low-alkaloid *pauper* locus, including one anchored in a candidate gene (*LaAT*), and six markers for anthracnose resistance, including three developed in this study (Lin et al. 2009; Rychel and Książkiewicz 2019; Rychel et al. 2019; Yang et al. 2010).

Unlike white lupin, narrow-leafed lupin exhibited high effectiveness of anthracnose resistance breeding, leading to the development of a large collection of resistant germplasm (Fischer et al. 2015; Yang et al. 2012). Three factors contributed to this achievement: (i) the monogenic inheritance of the resistance, (ii) the presence of resistance alleles in germplasm that had already been subjected to selection for regional adaptation, and (iii) the development and successful implementation of truly selective markers into breeding programs. The resistance to anthracnose in narrow-leafed lupin is controlled by several single dominant genes that were discovered in different germplasm resources, namely *Lanr1* in cv. Tanjil, *AnMan* in cv. Mandelup, and *LanrBo* in the breeding line Bo7212 (Fischer et al. 2015; Yang et al. 2004; Yang et al. 2008). Interestingly, none of these loci were localized in genome regions collinear to the white lupin regions carrying QTLs for anthracnose resistance (Książkiewicz et al. 2017). Nevertheless, annotation of white lupin genome regions carrying two major anthracnose resistance QTLs revealed the presence of protein domains which are typical for disease resistance genes (Bent 1996; Dangl and Jones 2001; Jones 2001). Moreover, a homolog of the *EDR2* gene conferring *Arabidopsis thaliana* resistance to biotrophic powdery mildew pathogen *Erysiphe cichoracearum* (Tang et al. 2005) was identified.

Narrow-leafed lupin breeding was also facilitated by the development of sequence-defined SSR-derived markers linked to key agronomic traits. These include soft seediness (Li et al. 2012a), reduced pod shattering (Boersma et al. 2007b; Boersma et al. 2009; Li et al. 2010; Li et al. 2012b), low alkaloid profile (Li et al. 2011), early flowering (Boersma et al. 2007a; Nelson et al. 2017), and resistance to various fungal diseases, including anthracnose (Yang et al. 2004; Yang et al. 2008; You et al. 2005), *Phomopsis* stem blight (Yang et al. 2002), and lupin rust (Sweetingham et al. 2005). These markers, except those for lupin rust, were subsequently implemented in Australian breeding programs. The development of low-cost high-throughput sequencing methods opened new possibilities for molecular genetics. Mass sequencing has been exploited in narrow-leafed lupin, to provide several markers linked to anthracnose and *Phomopsis* stem blight resistance genes. Some of these SNPs were revealed to have a lower recombination rate between the particular trait and marker loci than the previously developed SSR-based markers, and were implemented into breeding practice (Książkiewicz et al. 2020; Książkiewicz and Yang 2020; Yang et al. 2015a; Yang et al. 2015b; Yang et al. 2013a; Yang et al. 2013b; Zhou et al. 2018). All of these molecular resources have greatly contributed to the improvement of narrow-leafed lupin as a crop.

Marker-assisted selection in lupin breeding in Europe is currently at an initial stage, contrary to Australia, where it has been commenced for more than 15 years, targeting around 20,000 plants annually (Książkiewicz and Yang 2020; Yang and Buirchell 2008). Various techniques for polymorphism detection were implemented in the Australian breeding program, including 96-well polyacrylamide denaturing gel electrophoresis, CAPS/dCAPS, single-stranded conformation polymorphisms, high-resolution melting, and high-throughput allele-specific nanofluidic array (Boersma et al. 2009; Li et al. 2012b; Yang et al. 2015a; Yang et al. 2015b; Yang et al. 2002; Yang et al. 2013a; Yang et al. 2013b; Zhou et al. 2018). In this study, CAPS/dCAPS markers were developed, which are preferred in small-scale experiments (Shavrukov 2016). Moreover, other markers for white lupin agronomic traits are also based on PCR and electrophoresis (Książkiewicz et al. 2017; Lin et al. 2009; Rychel and Książkiewicz 2019; Rychel et al. 2019; Yang et al. 2010). However, such markers are relatively expensive per data point (about 1–1.5 USD per sample) and have limited capacity. As sequences of developed markers have been publicly released (see accession numbers LC416306–LC416345), selected SNPs can be transformed to the other system allowing the high-throughput approach. Popular medium-scale systems include PCR with TaqMan probes, Kompetitive Allele Specific PCR (KASP) and RNase H2 enzyme-based amplification (rhAmp) (Broccanello et al. 2018). These methods are considerably cheaper than CAPS and dCAPS (0.12–0.41 USD per sample), provided that the number of analyzed samples corresponds to the number of reactions supported by the assay mix (min. 2–10 thousand) (Ayalew et al. 2019).

### Genomic selection can assist marker-assisted selection of anthracnose resistance

In this study, QTL mapping was performed to investigate the association of the resistance score to specific parts of the genome. A valid alternative analysis was genome-wide association mapping (GWAS), which can be used to score the association between thousands of SNP markers and the desired trait. Thus, GWAS was recently exploited in *L. angustifolius*, highlighting novel candidate genes for phenology, growth, and yield traits (Plewiński et al. 2020). However, it was shown that GWAS can lead to false associations if the trait of interest is present in a very small proportion (< 5%) of the population (Sonah et al. 2015). QTL mapping does not present such a limitation and was, therefore, preferred for the trait in the test RIL population.

An additional aim of this study was investigating the ability of a whole-genome genomic regression approach to predict anthracnose disease resistance scores. This modeling approach is typically used for marker-based selection of genetically complex traits (Heffner et al. 2010) relative to crop yield or crop quality, and did prove promising to improve the selection efficiency for crop yield and key quality traits of various legume species (Annicchiarico et al. 2015; Annicchiarico et al. 2017b; Biazzi et al. 2017; Jarquin et al. 2014). This unprecedented attempt to apply genomic selection for a legume crop trait controlled mainly by a few major QTLs revealed predictive ability values in the range of 0.50–0.55. These values are comparable with those observed for several morphophysiological traits such as pod fertility, individual seed weight, plant height, leaf size, and mainstem proportion of seeds and number of leaves in a pioneer study focusing on a world collection of white lupin landraces (Annicchiarico et al. 2020). Results from the same germplasm collection or other material indicated that onset of flowering (also under oligogenic control) is highly predictable genomically (Annicchiarico et al. 2020), whereas the predictive ability of white lupin grain yield was in the range of 0.40–0.51. The current predictive ability values may justify the inclusion of this resistance trait in GBS-based genomic selection programs in which this trait is one of several target traits under selection, possibly combining the predicted values of the different target traits into a selection index. Our genome-wide predictions were obtained using trait-agnostic markers, leveraging the similitude of the selection candidate to a general “resistant genetic profile” to predict future resistance. Besides the possible practical interest of applying genomic selection for the resistance trait by the same operational tool used of other target traits (i.e., without adopting an additional marker-based tool specific for the trait), the whole-genome selection approach may also allow explaining some of the ~ 50% phenotypic variance that was not explained by the two observed QTLs, thereby adding some of the unavoidable missing heritability relative to QTLs (Brachi et al. 2011; Manolio et al. 2009) to the measurable contribution of the genetic background. For example, GBS-based genomic selection may address the possible presence of a third QTL for resistance to anthracnose (antr04_3) whose effect, however, did not reach statistical significance in composite interval mapping (Książkiewicz et al. 2017).

We noted that a lower number of markers (631, at 10% missing rate) resulted in predictive abilities higher than those obtained with more markers (e.g., 3230 at 50% missing rate). While this can be surprising at face value, it must be considered that the information provided by markers with more missing data is more noisy. In other terms, there is a trade-off between accepting more information at the cost of that information being of lower quality.

Breeding white lupin for anthracnose resistance without molecular selection has been an arduous process, as the polygenic control of major traits implies low frequency of desired phenotypes in the progeny. Moreover, anthracnose testing is largely destructive for seed samples, because of the impact of the disease and the lack of full immunity. In the present study, two types of molecular tools were developed: markers to focus essentially on this trait by the PCR-based procedure, and genomic selection to enable future multi-trait selection based on trait-specific models for material genotyped by GBS. These tools could enable the selection of germplasm carrying candidate Ethiopian alleles of two major anthracnose resistance loci what would decrease the number of lines subjected to disease screening. Also, they could prove valuable for locating other putatively tolerant material in germplasm collection. It should be noted that white lupin anthracnose resistance assays were performed using the approach exploiting spreader plants inoculated by spray inoculation, to mimic naturally occurring anthracnose (Phan et al. 2007; Yang et al. 2010) and an isolate *C. lupini* isolate 96A4 (IMI375715), classified into vegetative compatibility group 2 (Yang and Sweetingham 1998). Other methods, such as direct spraying of plants with spore suspension, inoculation of seeds before sowing, or injection of spores using a hypodermic needle to cotyledons or stems, were also used in lupin studies (Weimer 1952). Moreover, the Kiev Mutant × P27174 mapping population was phenotyped for resistance to anthracnose in Australia in wintertime, which is characterized by relatively low daily temperatures compared to those occurring in European regions where lupins are cultivated as a spring-sown crop. As the pathogenicity of *C. lupini* depends on the temperature pattern during infections and the strain of the pathogen (Dubrulle et al. 2020; Thomas et al. 2008), candidate lines selected by molecular markers should be subjected to disease resistance screening in local environment using domestic isolates.

## Electronic supplementary material

ESM 1 (DOCX 955 kb)

## Data Availability

All data generated in this study are included in this published article and its supplementary information files and in the DNA Data Bank of Japan (LC416306–LC416345).

## Abbreviations

ALB02: *L. albus* linkage group and chromosome
ALB04: *L. albus* linkage group and chromosome
*AnMan*: Anthracnose resistance gene present in *L. angustifolius* cultivar Mandelup
antr04_1: Anthracnose resistance locus present in *L. albus* linkage group ALB02
antr04_2: Anthracnose resistance locus present in *L. albus* linkage group ALB04
antr05_1: Anthracnose resistance locus present in *L. albus* linkage group ALB02
antr05_2: Anthracnose resistance locus present in *L. albus* linkage group ALB04
BLAST: Basic Local Alignment Search Tool
CC: Coiled-coil domain
*EDR2*: *ENHANCED DISEASE RESISTANCE 2*
GBS: Genotyping by sequencing
GWAS: Genome-wide association mapping
KASP: Kompetitive Allele Specific PCR
*Lanr1*: Anthracnose resistance gene present in *L. angustifolius* cultivars Tanjil and Wonga
*LanrBo*: Anthracnose resistance gene present in *L. angustifolius* breeding line Bo7212
LOD: Logarithm of the odds (used to estimate power of marker linkage)
LRR: Leucine-rich repeat
NBS: Nucleotide binding site
NLL-02: *L. angustifolius* linkage group and chromosome
NLL-20: *L. angustifolius* linkage group and chromosome
P27174: Anthracnose-resistant *L. albus* primitive landrace from Ethiopia
PVE: proportion of phenotypic variance explained by QTL
QTL: Quantitative trait locus
rhAmp: RNase H2 enzyme-based amplification
RIL: Recombinant inbred line
SNP: Single nucleotide polymorphism
SSR: Simple sequence repeat
STS: Sequence tagged site
TIR: Toll/interleukin-1 receptor
TM: Transmembrane domain
WANR1: Marker linked with anthracnose resistance locus in *L. albus* linkage group ALB02
WANR3: Marker linked with anthracnose resistance locus in *L. albus* linkage group ALB04

## References

[CR1] Adhikari KN, Buirchell BJ, Thomas GJ, Sweetingham MW, Yang H (2009). Identification of anthracnose resistance in *Lupinus albus* L. and its transfer from landraces to modern cultivars. Crop Pasture Sci.

[CR2] Adhikari KN, Thomas G, Diepeveen D, Trethowan R (2013). Overcoming the barriers of combining early flowering and anthracnose resistance in white lupin (*Lupinus albus* L.) for the Northern Agricultural Region of Western Australia. Crop Pasture Sci.

[CR3] Altschul SF, Gish W, Miller W, Myers EW, Lipman DJ (1990). Basic local alignment search tool. J Mol Biol.

[CR4] Annicchiarico P, Harzic N, Carroni AM (2010). Adaptation, diversity, and exploitation of global white lupin (*Lupinus albus* L.) landrace genetic resources. Field Crop Res.

[CR5] Annicchiarico P, Manunza P, Arnoldi A, Boschin G (2014). Quality of *Lupinus albus* L. (white lupin) seed: extent of genotypic and environmental effects. J Agric Food Chem.

[CR6] Annicchiarico P, Nazzicari N, Li X, Wei Y, Pecetti L, Brummer EC (2015). Accuracy of genomic selection for alfalfa biomass yield in different reference populations. BMC Genomics.

[CR7] Annicchiarico P, Nazzicari N, Pecetti L, Romani M, Ferrari B, Wei Y, Brummer EC (2017a) GBS-based genomic selection for pea grain yield under severe terminal drought. The Plant Genome 10 doi:10.3835/plantgenome2016.07.0072 10.3835/plantgenome2016.07.0072 28724076

[CR8] Annicchiarico P, Nazzicari N, Wei Y, Pecetti L, Brummer EC (2017). Genotyping-by-sequencing and its exploitation for forage and cool-season grain legume breeding. Front Plant Sci.

[CR9] Annicchiarico P, Nazzicari N, Ferrari B, Harzic N, Carroni AM, Romani M, Pecetti L (2019). Genomic prediction of grain yield in contrasting environments for white lupin genetic resources. Mol Breed.

[CR10] Annicchiarico P, Nazzicari N, Ferrari B, Singh KB, Kamphuis LG, Nelson MN (2020). Genetic and genomic resources in white lupin and the application of genomic selection. The Lupin Genome.

[CR11] Atnaf M, Tesfaye K, Dagne K, Wegary D (2015). Extent and pattern of genetic diversity in Ethiopian white lupin landraces for agronomical and phenological traits. Afr Crop Sci J.

[CR12] Atnaf M, Yao N, Martina K, Dagne K, Wegary D, Tesfaye K (2017). Molecular genetic diversity and population structure of Ethiopian white lupin landraces: implications for breeding and conservation. PLoS One.

[CR13] Ayalew H, Tsang PW, Chu C, Wang J, Liu S, Chen C, Ma X-F (2019). Comparison of TaqMan, KASP and rhAmp SNP genotyping platforms in hexaploid wheat. PLoS One.

[CR14] Bent AF (1996). Plant disease resistance genes: function meets structure. Plant Cell.

[CR15] Biazzi E, Nazzicari N, Pecetti L, Brummer EC, Palmonari A, Tava A, Annicchiarico P (2017). Genome-wide association mapping and genomic selection for alfalfa (*Medicago sativa*) forage quality traits. PLoS One.

[CR16] Boersma JG, Pallotta M, Li C, Buirchell BJ, Sivasithamparam K, Yang H (2005). Construction of a genetic linkage map using MFLP and identification of molecular markers linked to domestication genes in narrow-leafed lupin (*Lupinus angustifolius* L.). Cell Mol Biol Lett.

[CR17] Boersma JG, Buirchell BJ, Sivasithamparam K, Yang H (2007). Development of a sequence-specific PCR marker linked to the *Ku* gene which removes the vernalization requirement in narrow-leafed lupin. Plant Breed.

[CR18] Boersma JG, Buirchell BJ, Sivasithamparam K, Yang H (2007). Development of two sequence-specific PCR markers linked to the *le* gene that reduces pod shattering in narrow-leafed lupin (*Lupinus angustifolius* L.). Genet Mol Biol.

[CR19] Boersma JG, Nelson MN, Sivasithamparam K, Yang H (2009). Development of sequence-specific PCR markers linked to the *Tardus* gene that reduces pod shattering in narrow-leafed lupin (*Lupinus angustifolius* L.). Mol Breed.

[CR20] Boschin G, D’Agostina A, Annicchiarico P, Arnoldi A (2007). The fatty acid composition of the oil from *Lupinus albus* cv. Luxe as affected by environmental and agricultural factors. Eur Food Res Technol.

[CR21] Brachi B, Morris GP, Borevitz JO (2011). Genome-wide association studies in plants: the missing heritability is in the field. Genome Biol.

[CR22] Broccanello C, Chiodi C, Funk A, McGrath JM, Panella L, Stevanato P (2018). Comparison of three PCR-based assays for SNP genotyping in plants. Plant Methods.

[CR23] Confortin TC (2017). Extraction and composition of extracts obtained from *Lupinus albescens* using supercritical carbon dioxide and compressed liquefied petroleum gas. J Supercrit Fluids.

[CR24] Confortin TC, Todero I, Luft L, Soares JF, Mazutti MA, Zabot GL, Tres MV (2018). Importance of *Lupinus albescens* in agricultural and food-related areas: a review. 3 Biotech.

[CR25] Confortin TC (2019). Extracts from *Lupinus albescens*: antioxidant power and antifungal activity in vitro against phytopathogenic fungi. Environ Technol.

[CR26] Cowley R, Luckett DJ, Ash GJ, Harper JDI, Vipin CA, Raman H, Ellwood S (2014). Identification of QTLs associated with resistance to Phomopsis pod blight (*Diaporthe toxica*) in *Lupinus albus*. Breed Sci.

[CR27] Croxford AE, Rogers T, Caligari PDS, Wilkinson MJ (2008). High-resolution melt analysis to identify and map sequence-tagged site anchor points onto linkage maps: a white lupin (*Lupinus albus*) map as an exemplar. New Phytol.

[CR28] Dangl JL, Jones JD (2001) Plant pathogens and integrated defence responses to infection. Nature 411 doi:10.1038/35081161 10.1038/35081161 11459065

[CR29] Darling AC, Mau B, Blattner FR, Perna NT (2004). Mauve: multiple alignment of conserved genomic sequence with rearrangements. Genome Res.

[CR30] Dubrulle G (2020). Phylogenetic diversity and effect of temperature on pathogenicity of *Colletotrichum lupini*. Plant Dis.

[CR31] Endelman JB (2011). Ridge regression and other kernels for genomic selection with R package rrBLUP. Plant Genome.

[CR32] Erdemoglu N, Ozkan S, Tosun F (2007). Alkaloid profile and antimicrobial activity of *Lupinus angustifolius* L. alkaloid extract. Phytochem Rev.

[CR33] Fischer K (2015). Characterization and mapping of *LanrBo*: a locus conferring anthracnose resistance in narrow-leafed lupin (*Lupinus angustifolius* L.). Theor Appl Genet.

[CR34] Frencel IM (1998). Report on first detection of anthracnose (*Colletotrichum gloeosporioides*) on lupins in Poland. Plant Dis.

[CR35] Frencel IM, Lewartowska E, Czerwińska A (1997) First report on anthracnose diagnosis and *Colletotrichum* spp. identification in white lupin (*Lupinus albus* L.) infection in Poland. Diagnosis and Identification of Plant Pathogens: Proceedings of the 4th International Symposium of the European Foundation for Plant Pathology, September 9–12, 1996, Bonn, Germany. Springer Netherlands, Dordrecht. doi:10.1007/978-94-009-0043-1_63

[CR36] Gondran J, Hill GD (1996). Anthracnose of white lupin (*Lupinus albus*): European prospects for a future sustainable crop. Towards the 21st century. Proceedings of the 8th International Lupin Conference.

[CR37] Hane JK (2017). A comprehensive draft genome sequence for lupin (*Lupinus angustifolius*), an emerging health food: insights into plant-microbe interactions and legume evolution. Plant Biotechnol J.

[CR38] Harzic N, Huyghe C, Papineau J (1995). Dry matter accumulation and seed yield of dwarf autumn-sown white lupin (*Lupinus albus* L.). Can J Plant Sci.

[CR39] Heffner EL, Sorrells ME, Jannink J-L (2009). Genomic selection for crop improvement. Crop Sci.

[CR40] Heffner EL, Lorenz AJ, Jannink J-L, Sorrells ME (2010). Plant breeding with genomic selection: gain per unit time and cost. Crop Sci.

[CR41] Hufnagel B (2020). High-quality genome sequence of white lupin provides insight into soil exploration and seed quality. Nat Commun.

[CR42] Huyghe C, Papineau J (1990). Winter development of autumn sown white lupin: agronomic and breeding consequences. Agronomie.

[CR43] Jacob I, Feuerstein U, Heinz M, Schott M, Urbatzka P (2017). Evaluation of new breeding lines of white lupin with improved resistance to anthracnose. Euphytica.

[CR44] Jarquin D, Kocak K, Posadas L, Hyma K, Jedlicka J, Graef G, Lorenz A (2014). Genotyping by sequencing for genomic prediction in a soybean breeding population. BMC Genomics.

[CR45] Jones JD (2001). Putting knowledge of plant disease resistance genes to work. Curr Opin Plant Biol.

[CR46] Julier B, Huyghe C, Papineau J, Milford GFJ, Day JM, Billot C, Mangin P (1993). Seed yield and yield stability of determinate and indeterminate autumn-sown white lupins (*Lupinus albus*) grown at different locations in France and the UK. J Agric Sci.

[CR47] Kearse M (2012). Geneious Basic: an integrated and extendable desktop software platform for the organization and analysis of sequence data. Bioinformatics.

[CR48] Konieczny A, Ausubel FM (1993). A procedure for mapping *Arabidopsis* mutations using co-dominant ecotype-specific PCR-based markers. Plant J.

[CR49] Kroc M, Rybiński W, Wilczura P, Kamel KA, Kaczmarek Z, Barzyk P, Święcicki W (2017). Quantitative and qualitative analysis of alkaloids composition in the seeds of a white lupin (*Lupinus albus* L.) collection. Genet Resour Crop Evol.

[CR50] Książkiewicz M, Yang H, Singh KB, Kamphuis LG, Nelson MN (2020). Molecular marker resources supporting the Australian lupin breeding program. The Lupin genome.

[CR51] Książkiewicz M (2017). A high-density consensus linkage map of white lupin highlights synteny with narrow-leafed lupin and provides markers tagging key agronomic traits. Sci Rep.

[CR52] Książkiewicz M (2020). Validation of *Diaporthe toxica* resistance markers in European *Lupinus angustifolius* germplasm and identification of novel resistance donors for marker-assisted selection. J Appl Genet.

[CR53] Lambers H, Clements JC, Nelson MN (2013). How a phosphorus-acquisition strategy based on carboxylate exudation powers the success and agronomic potential of lupines (*Lupinus*, Fabaceae). Am J Bot.

[CR54] Li X, Renshaw D, Yang H, Yan G (2010). Development of a co-dominant DNA marker tightly linked to gene *tardus* conferring reduced pod shattering in narrow-leafed lupin (*Lupinus angustifolius* L.). Euphytica.

[CR55] Li X, Yang H, Buirchell B, Yan G (2011). Development of a DNA marker tightly linked to low-alkaloid gene *iucundus* in narrow-leafed lupin (*Lupinus angustifolius* L.) for marker-assisted selection. Crop Pasture Sci.

[CR56] Li X, Buirchell B, Yan G, Yang H (2012). A molecular marker linked to the *mollis* gene conferring soft-seediness for marker-assisted selection applicable to a wide range of crosses in lupin (*Lupinus angustifolius* L.) breeding. Mol Breed.

[CR57] Li X, Yang H, Yan G (2012). Development of a co-dominant DNA marker linked to the gene *lentus* conferring reduced pod shattering for marker-assisted selection in narrow-leafed lupin (*Lupinus angustifolius*) breeding. Plant Breed.

[CR58] Lin R (2009). Development of a sequence-specific PCR marker linked to the gene “pauper” conferring low-alkaloids in white lupin (*Lupinus albus* L.) for marker assisted selection. Mol Breed.

[CR59] Manolio TA (2009). Finding the missing heritability of complex diseases. Nature.

[CR60] Meuwissen TH, Hayes BJ, Goddard ME (2001). Prediction of total genetic value using genome-wide dense marker maps. Genetics.

[CR61] Nazzicari N, Biscarini F (2017) GROAN: genomic regression workbench. R package version 1.1.0. https://CRAN.R-project.org/package=GROAN.

[CR62] Nazzicari N, Biscarini F, Cozzi P, Brummer EC, Annicchiarico P (2016). Marker imputation efficiency for genotyping-by-sequencing data in rice (*Oryza sativa*) and alfalfa (*Medicago sativa*). Mol Breed.

[CR63] Neff MM, Neff JD, Chory J, Pepper AE (1998). dCAPS, a simple technique for the genetic analysis of single nucleotide polymorphisms: experimental applications in *Arabidopsis thaliana* genetics. Plant J.

[CR64] Neff MM, Turk E, Kalishman M (2002). Web-based primer design for single nucleotide polymorphism analysis. Trends Genet.

[CR65] Nelson MN (2017). The loss of vernalization requirement in narrow-leafed lupin is associated with a deletion in the promoter and de-repressed expression of a *Flowering Locus T* (*FT*) homologue. New Phytol.

[CR66] Nirenberg HI, Feiler U, Hagedorn G (2002). Description of *Colletotrichum lupini* comb. nov. in modern terms. Mycologia.

[CR67] O'Rourke JA (2013). An RNA-Seq transcriptome analysis of orthophosphate-deficient white lupin reveals novel insights into phosphorus acclimation in plants. Plant Physiol.

[CR68] Osuna-Cruz CM (2018). PRGdb 3.0: a comprehensive platform for prediction and analysis of plant disease resistance genes. Nucleic Acids Res.

[CR69] Papineau J, Huyghe C (2004). Le lupin doux protéagineux.

[CR70] Phan HTT, Ellwood SR, Adhikari K, Nelson MN, Oliver RP (2007). The first genetic and comparative map of white lupin (*Lupinus albus* L.): identification of QTLs for anthracnose resistance and flowering time, and a locus for alkaloid content DNA. Res.

[CR71] Plewiński P et al. (2020) Innovative transcriptome-based genotyping highlights environmentally responsive genes for phenology, growth and yield in a non-model grain legume. Plant, Cell Environ: (in press) doi:10.1111/pce.13880 10.1111/pce.13880 32885839

[CR72] Raman R, Cowley R, Raman H, Luckett DJ (2014) Analyses using SSR and DArT molecular markers reveal that Ethiopian accessions of white lupin (*Lupinus albus* L.) represent a unique genepool. Open Journal of Genetics:87-98 doi:10.4236/ojgen.2014.42012

[CR73] Rogers DJ, Tanimoto TT (1960). A computer program for classifying plants. Science.

[CR74] Romeo FV, Fabroni S, Ballistreri G, Muccilli S, Spina A, Rapisarda P (2018). Characterization and antimicrobial activity of alkaloid extracts from seeds of different genotypes of *Lupinus* spp. Sustainability.

[CR75] Rychel S, Książkiewicz M (2019). Development of gene-based molecular markers tagging low alkaloid pauper locus in white lupin (*Lupinus albus* L.). J Appl Genet.

[CR76] Rychel S, Książkiewicz M, Tomaszewska M, Bielski W, Wolko B (2019). *FLOWERING LOCUS T*, *GIGANTEA*, *SEPALLATA* and *FRIGIDA* homologs are candidate genes involved in white lupin (*Lupinus albus* L.) early flowering. Mol Breed.

[CR77] Searle SR, Casella G, McCulloch CE (2008). Variance components. Wiley Series in Probability and Statistics.

[CR78] Shavrukov Y (2016). Comparison of SNP and CAPS markers application in genetic research in wheat and barley. BMC Plant Biol.

[CR79] Sokal RR, Michener CD (1958) A statistical method for evaluating systematic relationships. University of Kansas Scientific Bulletin 28:1409-1438

[CR80] Sonah H, O'Donoughue L, Cober E, Rajcan I, Belzile F (2015). Identification of loci governing eight agronomic traits using a GBS-GWAS approach and validation by QTL mapping in soya bean. Plant Biotechnol J.

[CR81] Stam P (1993). Construction of integrated genetic linkage maps by means of a new computer package: Join Map. Plant J.

[CR82] Stekhoven DJ, Bühlmann P (2012). MissForest—non-parametric missing value imputation for mixed-type data. Bioinformatics.

[CR83] Sweetingham M, Cowling WA, Buirchell B, Brown A, Shivas R (1995). Anthracnose of lupins in Western Australia. Australas Plant Path.

[CR84] Sweetingham MW, Yang H, Buirchell BJ, Shea G, Shield I, van Santen E, Hill GD (2005). Resistance to rust in narrow-leafed lupin and development of molecular markers. México, where old and new world lupins meet. 11th International Lupin Conference.

[CR85] Talhinhas P, Baroncelli R, Floch GL (2016). Anthracnose of lupins caused by *Colletotrichum lupini*: a recent disease and a successful worldwide pathogen. J Plant Pathol.

[CR86] Tang D, Ade J, Frye CA, Innes RW (2005). Regulation of plant defense responses in Arabidopsis by EDR2, a PH and START domain-containing protein. Plant J.

[CR87] Thiel T, Kota R, Grosse I, Stein N, Graner A (2004). SNP2CAPS: a SNP and INDEL analysis tool for CAPS marker development. Nucleic Acids Res.

[CR88] Thomas GJ, Sweetingham MW, Yang HA, Speijers J (2008). Effect of temperature on growth of Colletotrichum lupini and on anthracnose infection and resistance in lupins. Australas Plant Path.

[CR89] Untergasser A, Nijveen H, Rao X, Bisseling T, Geurts R, Leunissen JAM (2007). Primer3Plus, an enhanced web interface to Primer3. Nucleic Acids Res.

[CR90] van Ooijen JW (1992). Accuracy of mapping quantitative trait loci in autogamous species. Theor Appl Genet.

[CR91] Vipin CA (2013). Construction of integrated linkage map of a recombinant inbred line population of white lupin (*Lupinus albus* L.). Breed Sci.

[CR92] Voorrips RE (2002). MapChart: software for the graphical presentation of linkage maps and QTLs. J Hered.

[CR93] Weimer JL (1943). Anthracnose of lupines. Phytopathology.

[CR94] Weimer JL (1952) Lupine anthracnose. Circular No. 904. U.S. Department of Agriculture, Washington D.C.

[CR95] Yang H, Buirchell B, Palta JA, Berger JB (2008). Strategies in developing molecular markers for marker assisted selection in lupin breeding in Australia. Lupins for health and wealth, Proceedings of the 12th International Lupin Conference.

[CR96] Yang H, Sweetingham MW (1998). The taxonomy of Colletotrichum isolates associated with lupin anthracnose. Aust J Agric Res.

[CR97] Yang H, Shankar M, Buirchell J, Sweetingham W, Caminero C, Smith C (2002). Development of molecular markers using MFLP linked to a gene conferring resistance to *Diaporthe toxica* in narrow-leafed lupin (*Lupinus angustifolius* L.). Theor Appl Genet.

[CR98] Yang H, Boersma JG, You M, Buirchell BJ, Sweetingham MW (2004). Development and implementation of a sequence-specific PCR marker linked to a gene conferring resistance to anthracnose disease in narrow-leafed lupin (*Lupinus angustifolius* L.). Mol Breed.

[CR99] Yang H, Renshaw D, Thomas G, Buirchell B, Sweetingham M (2008). A strategy to develop molecular markers applicable to a wide range of crosses for marker assisted selection in plant breeding: a case study on anthracnose disease resistance in lupin (*Lupinus angustifolius* L.). Mol Breed.

[CR100] Yang H (2010). Development of sequence-specific PCR markers associated with a polygenic controlled trait for marker-assisted selection using a modified selective genotyping strategy: a case study on anthracnose disease resistance in white lupin (*Lupinus albus* L.). Mol Breed.

[CR101] Yang H, Tao Y, Zheng Z, Li C, Sweetingham MW, Howieson JG (2012). Application of next-generation sequencing for rapid marker development in molecular plant breeding: a case study on anthracnose disease resistance in *Lupinus angustifolius* L. BMC Genomics.

[CR102] Yang H (2013). Rapid development of molecular markers by next-generation sequencing linked to a gene conferring phomopsis stem blight disease resistance for marker-assisted selection in lupin (*Lupinus angustifolius* L.) breeding. Theor Appl Genet.

[CR103] Yang H (2013). Draft genome sequence, and a sequence-defined genetic linkage map of the legume crop species *Lupinus angustifolius* L. PLoS One.

[CR104] Yang H (2015). Application of whole genome re-sequencing data in the development of diagnostic DNA markers tightly linked to a disease-resistance locus for marker-assisted selection in lupin (*Lupinus angustifolius*). BMC Genomics.

[CR105] Yang H, Li C, Lam HM, Clements J, Yan G, Zhao S (2015). Sequencing consolidates molecular markers with plant breeding practice. Theor Appl Genet.

[CR106] You M, Boersma JG, Buirchell BJ, Sweetingham MW, Siddique KHM, Yang H (2005). A PCR-based molecular marker applicable for marker-assisted selection for anthracnose disease resistance in lupin breeding. Cell Mol Biol Lett.

[CR107] Zhou G (2018). Construction of an ultra-high density consensus genetic map, and enhancement of the physical map from genome sequencing in *Lupinus angustifolius*. Theor Appl Genet.

