# Supplementary material for: Development of PCR-based markers and whole-genome selection model for anthracnose resistance in white lupin (Lupinus albus L.)
Source: J Appl Genet. 2020 Sep 23;61(4):531–45. doi: 10.1007/s13353-020-00585-1 (PMC7652745; doi:10.1007/s13353-020-00585-1)
Supplement: Supplementary file 1 — (DOCX 955 kb) [file 13353_2020_585_MOESM1_ESM.docx]

**Supplementary Tables and Figures**

**Development of PCR-based markers and whole-genome selection model for anthracnose resistance in white lupin (*Lupinus albus* L.)**

Sandra Rychel-Bielska^1,2^, Nelson Nazzicari^3^, Piotr Plewiński^2^, Wojciech Bielski^2^, Paolo Annicchiarico^3^, Michał Książkiewicz^2*^

1. Department of Genetics, Plant Breeding and Seed Production, Wroclaw University of Environmental and Life Sciences, (Plac Grunwaldzki 24A, 50-363 Wrocław, Poland)

2. Institute of Plant Genetics Polish Academy of Sciences (Strzeszyńska 34, 60-479 Poznań, Poland)

3. CREA-FLC, Council for Agricultural Research and Economics, Research Centre for Fodder Crops and Dairy Production (Viale Piacenza 29, 26900 Lodi, Italy)

*correspondence: [mksi@igr.poznan.pl](mailto:mksi@igr.poznan.pl)

[Supplementary Table S1. The list of developed PCR-based markers for white lupin anthracnose resistance QTLs with all possible restriction enzymes detecting polymorphic loci and expected cleavage products. 2](#_Toc49941228)

[Supplementary Table S2. Parameters for linkage mapping. 11](#_Toc49941229)

[Supplementary Table S3. Parameters for QTL mapping. 12](#_Toc49941230)

[Supplementary Table S4. The list of white lupin lines used for anthracnose resistance marker validation. 13](#_Toc49941231)

[Supplementary Table S5. The summary of transformation of white lupin GBS-derived markers localized in anthracnose resistance loci to PCR-based markers. 15](#_Toc49941232)

[Supplementary Table S6. The list of white lupin transcriptome sequences anchored to white lupin GBS marker sequences with alignment data. 17](#_Toc49941233)

[Supplementary Table S7. The list of narrow-leafed lupin genome sequences anchored to designed white lupin PCR marker sequences with alignment data. 18](#_Toc49941234)

[Supplementary Table S8. Updated marker segregation data for white lupin RIL population. 19](#_Toc49941235)

[Supplementary Table S9. List of updated markers with percentages of RILs scored by GBS and PCR-based methods, Chi-square P-values for segregation distortion, previous and updated linkage group positions and LOD scores to adjacent loci on the updated map. 24](#_Toc49941236)

[Supplementary Table S10. Updated white lupin linkage groups ALB02 and ALB04. 25](#_Toc49941237)

[Supplementary Table S11. Marker scores for white lupin lines from world germplasm collection. 30](#_Toc49941238)

[Supplementary Table S12. Genes identified in the regions of white lupin genome carrying anthracnose resistance loci. 33](#_Toc49941239)

[Supplementary Table S13. Kinase, coiled-coil (CC), leucine-rich repeat (LRR), nucleotide binding site (NBS), Toll/interleukin-1 receptor (TIR) and transmembrane (TM) domains identified in genes localized in the regions of white lupin genome carrying anthracnose resistance loci. 42](#_Toc49941240)

[Supplementary Figure S1. Agarose gel electrophoresis of positively validated PCR-based markers for two major anthracnose resistance QTL loci, antr04_1/antr05_1 (TP222136 and TP47110) and antr04_2/antr05_2 (TP338761). 43](#_Toc49941241)

# Supplementary Table S1. The list of developed PCR-based markers for white lupin anthracnose resistance QTLs with all possible restriction enzymes detecting polymorphic loci and expected cleavage products.

P, line P27174; K, Kiev Mutant.

| **PCR marker sequence** | **PCR product length** | **Enzyme** | **Restriction sites** | **Restriction products** |
| --- | --- | --- | --- | --- |
| TP106254_P | 118 | Aco12261II | 65 | 65,53 |
| TP106254_K | 118 | Aco12261II |  | 118 |
| TP106254_P | 118 | AcsI | 50 | 68,50 |
| TP106254_K | 118 | AcsI | 50,66 | 52,50,16 |
| TP106254_P | 118 | ApoI | 50 | 68,50 |
| TP106254_K | 118 | ApoI | 50,66 | 52,50,16 |
| TP106254_P | 118 | MluCI | 50 | 68,50 |
| TP106254_K | 118 | MluCI | 50,66 | 52,50,16 |
| TP106254_P | 118 | Sse9I | 50 | 68,50 |
| TP106254_K | 118 | Sse9I | 50,66 | 52,50,16 |
| TP106254_P | 118 | TasI | 50 | 68,50 |
| TP106254_K | 118 | TasI | 50,66 | 52,50,16 |
| TP106254_P | 118 | TspEI | 50 | 68,50 |
| TP106254_K | 118 | TspEI | 50,66 | 52,50,16 |
| TP106254_P | 118 | XapI | 50 | 68,50 |
| TP106254_K | 118 | XapI | 50,66 | 52,50,16 |
| TP149038_P | 100 | AgsI | 40 | 60,40 |
| TP149038_K | 100 | AgsI |  | 100 |
| TP149038_P | 100 | MfeI | 35 | 65,35 |
| TP149038_K | 100 | MfeI |  | 100 |
| TP149038_P | 100 | MluCI | 35,47 | 53,35,12 |
| TP149038_K | 100 | MluCI | 47 | 53,47 |
| TP149038_P | 100 | MunI | 35 | 65,35 |
| TP149038_K | 100 | MunI |  | 100 |
| TP149038_P | 100 | Sse9I | 35,47 | 53,35,12 |
| TP149038_K | 100 | Sse9I | 47 | 53,47 |
| TP149038_P | 100 | TasI | 35,47 | 53,35,12 |
| TP149038_K | 100 | TasI | 47 | 53,47 |
| TP149038_P | 100 | TspEI | 35,47 | 53,35,12 |
| TP149038_K | 100 | TspEI | 47 | 53,47 |
| TP222136_P | 210 | AfaI | 150,171 | 150,39,21 |
| TP222136_K | 210 | AfaI | 150 | 150,60 |
| TP222136_P | 210 | AluBI |  | 210 |
| TP222136_K | 210 | AluBI | 168 | 168,42 |
| TP222136_P | 210 | AluI |  | 210 |
| TP222136_K | 210 | AluI | 168 | 168,42 |
| TP222136_P | 210 | Csp6I | 149,170 | 149,40,21 |
| TP222136_K | 210 | Csp6I | 149 | 149,61 |
| TP222136_P | 210 | CviJI | 183 | 183,27 |
| TP222136_K | 210 | CviJI | 168,183 | 168,27,15 |
| TP222136_P | 210 | CviKI-1 | 183 | 183,27 |
| TP222136_K | 210 | CviKI-1 | 168,183 | 168,27,15 |
| TP222136_P | 210 | CviQI | 149,170 | 149,40,21 |
| TP222136_K | 210 | CviQI | 149 | 149,61 |
| TP222136_P | 210 | HpyAXIV | 169 | 169,41 |
| TP222136_K | 210 | HpyAXIV |  | 210 |
| TP222136_P | 210 | PabI | 151,172 | 151,38,21 |
| TP222136_K | 210 | PabI | 151 | 151,59 |
| TP222136_P | 210 | RsaI | 150,171 | 150,39,21 |
| TP222136_K | 210 | RsaI | 150 | 150,60 |
| TP222136_P | 210 | RsaNI | 149,170 | 149,40,21 |
| TP222136_K | 210 | RsaNI | 149 | 149,61 |
| TP222136_P | 210 | SetI | 56,175 | 119,56,35 |
| TP222136_K | 210 | SetI | 56,170,175 | 114,56,35,5 |
| TP229924_P | 84 | AfaI |  | 84 |
| TP229924_K | 84 | AfaI | 30 | 54,30 |
| TP229924_P | 84 | AspBHI | 19,36,40,65,67 | 25,19,17,17,4,2 |
| TP229924_K | 84 | AspBHI | 19,36,65,67 | 29,19,17,17,2 |
| TP229924_P | 84 | BsaAI |  | 84 |
| TP229924_K | 84 | BsaAI | 28 | 56,28 |
| TP229924_P | 84 | BstBAI |  | 84 |
| TP229924_K | 84 | BstBAI | 28 | 56,28 |
| TP229924_P | 84 | BstC8I | 27 | 57,27 |
| TP229924_K | 84 | BstC8I |  | 84 |
| TP229924_P | 84 | Cac8I | 27 | 57,27 |
| TP229924_K | 84 | Cac8I |  | 84 |
| TP229924_P | 84 | Csp6I |  | 84 |
| TP229924_K | 84 | Csp6I | 29 | 55,29 |
| TP229924_P | 84 | CviQI |  | 84 |
| TP229924_K | 84 | CviQI | 29 | 55,29 |
| TP229924_P | 84 | Hpy166II |  | 84 |
| TP229924_K | 84 | Hpy166II | 31 | 53,31 |
| TP229924_P | 84 | Hpy8I |  | 84 |
| TP229924_K | 84 | Hpy8I | 31 | 53,31 |
| TP229924_P | 84 | HpyCH4IV |  | 84 |
| TP229924_K | 84 | HpyCH4IV | 27 | 57,27 |
| TP229924_P | 84 | HpySE526I |  | 84 |
| TP229924_K | 84 | HpySE526I | 27 | 57,27 |
| TP229924_P | 84 | MaeII |  | 84 |
| TP229924_K | 84 | MaeII | 27 | 57,27 |
| TP229924_P | 84 | MjaIV |  | 84 |
| TP229924_K | 84 | MjaIV | 31 | 53,31 |
| TP229924_P | 84 | PabI |  | 84 |
| TP229924_K | 84 | PabI | 31 | 53,31 |
| TP229924_P | 84 | Pfl8569I | 27 | 57,27 |
| TP229924_K | 84 | Pfl8569I |  | 84 |
| TP229924_P | 84 | Ppu21I |  | 84 |
| TP229924_K | 84 | Ppu21I | 28 | 56,28 |
| TP229924_P | 84 | PsuGI | 29 | 55,29 |
| TP229924_K | 84 | PsuGI |  | 84 |
| TP229924_P | 84 | RsaI |  | 84 |
| TP229924_K | 84 | RsaI | 30 | 54,30 |
| TP229924_P | 84 | RsaNI |  | 84 |
| TP229924_K | 84 | RsaNI | 29 | 55,29 |
| TP229924_P | 84 | SetI |  | 84 |
| TP229924_K | 84 | SetI | 30 | 54,30 |
| TP229924_P | 84 | TaiI |  | 84 |
| TP229924_K | 84 | TaiI | 30 | 54,30 |
| TP237794_P | 66 | BstDEI | 9,24,39 | 27,15,15,9 |
| TP237794_K | 66 | BstDEI | 9,24 | 42,15,9 |
| TP237794_P | 66 | Cch467III | 36 | 36,30 |
| TP237794_K | 66 | Cch467III |  | 66 |
| TP237794_P | 66 | DdeI | 9,24,39 | 27,15,15,9 |
| TP237794_K | 66 | DdeI | 9,24 | 42,15,9 |
| TP237794_P | 66 | Hpy99XXII | 44 | 44,22 |
| TP237794_K | 66 | Hpy99XXII |  | 66 |
| TP237794_P | 66 | HpyF3I | 9,24,39 | 27,15,15,9 |
| TP237794_K | 66 | HpyF3I | 9,24 | 42,15,9 |
| TP237794_P | 66 | MseI |  | 66 |
| TP237794_K | 66 | MseI | 39 | 39,27 |
| TP237794_P | 66 | SaqAI |  | 66 |
| TP237794_K | 66 | SaqAI | 39 | 39,27 |
| TP237794_P | 66 | Tru1I |  | 66 |
| TP237794_K | 66 | Tru1I | 39 | 39,27 |
| TP237794_P | 66 | Tru9I |  | 66 |
| TP237794_K | 66 | Tru9I | 39 | 39,27 |
| TP23903_P | 64 | BseGI | 42 | 42,22 |
| TP23903_K | 64 | BseGI |  | 64 |
| TP23903_P | 64 | BslFI |  | 64 |
| TP23903_K | 64 | BslFI | 49 | 49,15 |
| TP23903_P | 64 | BsmFI |  | 64 |
| TP23903_K | 64 | BsmFI | 49 | 49,15 |
| TP23903_P | 64 | BstF5I | 42 | 42,22 |
| TP23903_K | 64 | BstF5I |  | 64 |
| TP23903_P | 64 | BtsCI | 42 | 42,22 |
| TP23903_K | 64 | BtsCI |  | 64 |
| TP23903_P | 64 | EcoBLMcrX |  | 64 |
| TP23903_K | 64 | EcoBLMcrX | 39 | 39,25 |
| TP23903_P | 64 | FaqI |  | 64 |
| TP23903_K | 64 | FaqI | 49 | 49,15 |
| TP23903_P | 64 | FinI |  | 64 |
| TP23903_K | 64 | FinI | 36 | 36,28 |
| TP23903_P | 64 | FokI | 49 | 49,15 |
| TP23903_K | 64 | FokI |  | 64 |
| TP23903_P | 64 | Hpy99XIV-mut1 |  | 64 |
| TP23903_K | 64 | Hpy99XIV-mut1 | 38 | 38,26 |
| TP23903_P | 64 | PsuGI | 32,34 | 32,30,2 |
| TP23903_K | 64 | PsuGI | 32,34,40 | 32,24,6,2 |
| TP23903_P | 64 | RpaB5I |  | 64 |
| TP23903_K | 64 | RpaB5I | 59 | 59,5 |
| TP23903_P | 64 | Sth20745III |  | 64 |
| TP23903_K | 64 | Sth20745III | 38 | 38,26 |
| TP23903_P | 64 | StsI | 50 | 50,14 |
| TP23903_K | 64 | StsI |  | 64 |
| TP23903_P | 64 | TspARh3I |  | 64 |
| TP23903_K | 64 | TspARh3I | 38 | 38,26 |
| TP254603_P | 101 | BmsI | 39 | 62,39 |
| TP254603_K | 101 | BmsI | 39,48 | 53,39,9 |
| TP254603_P | 101 | BscAI | 38 | 63,38 |
| TP254603_K | 101 | BscAI | 38,49 | 52,38,11 |
| TP254603_P | 101 | HpyAXVI-mut1 | 54 | 54,47 |
| TP254603_K | 101 | HpyAXVI-mut1 |  | 101 |
| TP254603_P | 101 | HpyAXVI-mut2 |  | 101 |
| TP254603_K | 101 | HpyAXVI-mut2 | 54 | 54,47 |
| TP254603_P | 101 | LweI | 39 | 62,39 |
| TP254603_K | 101 | LweI | 39,48 | 53,39,9 |
| TP254603_P | 101 | MseI | 52 | 52,49 |
| TP254603_K | 101 | MseI |  | 101 |
| TP254603_P | 101 | SaqAI | 52 | 52,49 |
| TP254603_K | 101 | SaqAI |  | 101 |
| TP254603_P | 101 | SfaNI | 39 | 62,39 |
| TP254603_K | 101 | SfaNI | 39,48 | 53,39,9 |
| TP254603_P | 101 | Tru1I | 52 | 52,49 |
| TP254603_K | 101 | Tru1I |  | 101 |
| TP254603_P | 101 | Tru9I | 52 | 52,49 |
| TP254603_K | 101 | Tru9I |  | 101 |
| TP26007_P | 107 | AjiI |  | 107 |
| TP26007_K | 107 | AjiI | 29 | 78,29 |
| TP26007_P | 107 | BmgBI |  | 107 |
| TP26007_K | 107 | BmgBI | 29 | 78,29 |
| TP26007_P | 107 | BtrI |  | 107 |
| TP26007_K | 107 | BtrI | 29 | 78,29 |
| TP26007_P | 107 | CviAII | 29 | 78,29 |
| TP26007_K | 107 | CviAII |  | 107 |
| TP26007_P | 107 | FaeI | 32 | 75,32 |
| TP26007_K | 107 | FaeI |  | 107 |
| TP26007_P | 107 | FaiI | 30,63,78 | 33,30,29,15 |
| TP26007_K | 107 | FaiI | 63,78 | 63,29,15 |
| TP26007_P | 107 | FatI | 28 | 79,28 |
| TP26007_K | 107 | FatI |  | 107 |
| TP26007_P | 107 | Hin1II | 32 | 75,32 |
| TP26007_K | 107 | Hin1II |  | 107 |
| TP26007_P | 107 | HpyCH4IV |  | 107 |
| TP26007_K | 107 | HpyCH4IV | 28 | 79,28 |
| TP26007_P | 107 | HpySE526I |  | 107 |
| TP26007_K | 107 | HpySE526I | 28 | 79,28 |
| TP26007_P | 107 | Hsp92II | 32 | 75,32 |
| TP26007_K | 107 | Hsp92II |  | 107 |
| TP26007_P | 107 | MaeII |  | 107 |
| TP26007_K | 107 | MaeII | 28 | 79,28 |
| TP26007_P | 107 | NlaCI |  | 107 |
| TP26007_K | 107 | NlaCI | 10 | 97,10 |
| TP26007_P | 107 | NlaIII | 32 | 75,32 |
| TP26007_K | 107 | NlaIII |  | 107 |
| TP26007_P | 107 | SetI | 50,86 | 50,36,21 |
| TP26007_K | 107 | SetI | 31,50,86 | 36,31,21,19 |
| TP26007_P | 107 | TaiI |  | 107 |
| TP26007_K | 107 | TaiI | 31 | 76,31 |
| TP272081_P | 64 | Asi256I | 38 | 38,26 |
| TP272081_K | 64 | Asi256I |  | 64 |
| TP272081_P | 64 | Bsp143I | 37 | 37,27 |
| TP272081_K | 64 | Bsp143I |  | 64 |
| TP272081_P | 64 | BssMI | 37 | 37,27 |
| TP272081_K | 64 | BssMI |  | 64 |
| TP272081_P | 64 | BstKTI | 40 | 40,24 |
| TP272081_K | 64 | BstKTI |  | 64 |
| TP272081_P | 64 | BstMBI | 37 | 37,27 |
| TP272081_K | 64 | BstMBI |  | 64 |
| TP272081_P | 64 | ChaI | 41 | 41,23 |
| TP272081_K | 64 | ChaI |  | 64 |
| TP272081_P | 64 | DpnI | 39 | 39,25 |
| TP272081_K | 64 | DpnI |  | 64 |
| TP272081_P | 64 | DpnII | 37 | 37,27 |
| TP272081_K | 64 | DpnII |  | 64 |
| TP272081_P | 64 | Kzo9I | 37 | 37,27 |
| TP272081_K | 64 | Kzo9I |  | 64 |
| TP272081_P | 64 | MalI | 39 | 39,25 |
| TP272081_K | 64 | MalI |  | 64 |
| TP272081_P | 64 | MboI | 37 | 37,27 |
| TP272081_K | 64 | MboI |  | 64 |
| TP272081_P | 64 | NdeII | 37 | 37,27 |
| TP272081_K | 64 | NdeII |  | 64 |
| TP272081_P | 64 | Sau3AI | 37 | 37,27 |
| TP272081_K | 64 | Sau3AI |  | 64 |
| TP272531_P | 161 | AluBI | 22,34,66,124 | 58,37,32,22,12 |
| TP272531_K | 161 | AluBI | 22,34,53,66,124 | 58,37,22,19,13,12 |
| TP272531_P | 161 | AluI | 22,34,66,124 | 58,37,32,22,12 |
| TP272531_K | 161 | AluI | 22,34,53,66,124 | 58,37,22,19,13,12 |
| TP272531_P | 161 | BseMII |  | 161 |
| TP272531_K | 161 | BseMII | 38 | 123,38 |
| TP272531_P | 161 | BspCNI |  | 161 |
| TP272531_K | 161 | BspCNI | 39 | 122,39 |
| TP272531_P | 161 | BstDEI |  | 161 |
| TP272531_K | 161 | BstDEI | 49 | 112,49 |
| TP272531_P | 161 | CviJI | 22,34,66,124,158 | 58,34,32,22,12,3 |
| TP272531_K | 161 | CviJI | 22,34,53,66,124,158 | 58,34,22,19,13,12,3 |
| TP272531_P | 161 | CviKI-1 | 22,34,66,124,158 | 58,34,32,22,12,3 |
| TP272531_K | 161 | CviKI-1 | 22,34,53,66,124,158 | 58,34,22,19,13,12,3 |
| TP272531_P | 161 | DdeI |  | 161 |
| TP272531_K | 161 | DdeI | 49 | 112,49 |
| TP272531_P | 161 | HpyF3I |  | 161 |
| TP272531_K | 161 | HpyF3I | 49 | 112,49 |
| TP291372_D | 105 | BstMWI | 47 | 58,47 |
| TP291372_K | 105 | BstMWI | 47,56 | 49,47,9 |
| TP291372_D | 105 | HpyF10VI | 47 | 58,47 |
| TP291372_K | 105 | HpyF10VI | 47,56 | 49,47,9 |
| TP291372_D | 105 | MfeI |  | 105 |
| TP291372_K | 105 | MfeI | 54 | 54,51 |
| TP291372_D | 105 | MunI |  | 105 |
| TP291372_K | 105 | MunI | 54 | 54,51 |
| TP291372_D | 105 | MwoI | 47 | 58,47 |
| TP291372_K | 105 | MwoI | 47,56 | 49,47,9 |
| TP291372_D | 105 | SetI | 62,68 | 62,37,6 |
| TP291372_K | 105 | SetI | 68 | 68,37 |
| TP327093_P | 95 | CviRI | 36 | 59,36 |
| TP327093_K | 95 | CviRI |  | 95 |
| TP327093_P | 95 | HpyCH4V | 36 | 59,36 |
| TP327093_K | 95 | HpyCH4V |  | 95 |
| TP327093_P | 95 | MaeIII |  | 95 |
| TP327093_K | 95 | MaeIII | 37 | 58,37 |
| TP327093_P | 95 | PsuGI | 13,48,50,71 | 35,24,21,13,2 |
| TP327093_K | 95 | PsuGI | 13,36,48,50,71 | 24,23,21,13,12,2 |
| TP327093_P | 95 | Rsp008IV |  | 95 |
| TP327093_K | 95 | Rsp008IV | 36 | 59,36 |
| TP327093_P | 95 | SenSARA26III |  | 95 |
| TP327093_K | 95 | SenSARA26III | 36 | 59,36 |
| TP338761_P | 111 | BspD6I | 33 | 78,33 |
| TP338761_K | 111 | BspD6I | 42 | 69,42 |
| TP338761_P | 111 | CjeNII |  | 111 |
| TP338761_K | 111 | CjeNII | 33 | 78,33 |
| TP338761_P | 111 | CjePI | 95 | 95,16 |
| TP338761_K | 111 | CjePI | 52,95 | 52,43,16 |
| TP338761_P | 111 | CjuII |  | 111 |
| TP338761_K | 111 | CjuII | 32 | 79,32 |
| TP338761_P | 111 | HinfI | 10,38 | 73,28,10 |
| TP338761_K | 111 | HinfI | 10,34 | 77,24,10 |
| TP338761_P | 111 | MlyI | 47 | 64,47 |
| TP338761_K | 111 | MlyI | 28 | 83,28 |
| TP338761_P | 111 | PleI | 46 | 65,46 |
| TP338761_K | 111 | PleI | 29 | 82,29 |
| TP338761_P | 111 | PpsI | 46 | 65,46 |
| TP338761_K | 111 | PpsI | 29 | 82,29 |
| TP338761_P | 111 | SchI | 47 | 64,47 |
| TP338761_K | 111 | SchI | 28 | 83,28 |
| TP364001_P | 193 | AchA6III | 28 | 165,28 |
| TP364001_K | 193 | AchA6III | 28,76 | 117,48,28 |
| TP364001_P | 193 | Adh6U21I | 5,77 | 116,72,5 |
| TP364001_K | 193 | Adh6U21I | 5 | 188,5 |
| TP364001_P | 193 | BstC8I |  | 193 |
| TP364001_K | 193 | BstC8I | 75 | 118,75 |
| TP364001_P | 193 | Cac8I |  | 193 |
| TP364001_K | 193 | Cac8I | 75 | 118,75 |
| TP364001_P | 193 | CviJI | 29 | 164,29 |
| TP364001_K | 193 | CviJI | 29,77 | 116,48,29 |
| TP364001_P | 193 | CviKI-1 | 29 | 164,29 |
| TP364001_K | 193 | CviKI-1 | 29,77 | 116,48,29 |
| TP364001_P | 193 | DrdII | 11,77 | 116,66,11 |
| TP364001_K | 193 | DrdII | 11 | 182,11 |
| TP364001_P | 193 | Nal45188II | 75 | 118,75 |
| TP364001_K | 193 | Nal45188II |  | 193 |
| TP364001_P | 193 | Pfl8569I |  | 193 |
| TP364001_K | 193 | Pfl8569I | 75 | 118,75 |
| TP3712_P | 200 | AchA6III | 172 | 172,28 |
| TP3712_K | 200 | AchA6III | 124,172 | 124,48,28 |
| TP3712_P | 200 | Adh6U21I | 123,195 | 123,72,5 |
| TP3712_K | 200 | Adh6U21I | 195 | 195,5 |
| TP3712_P | 200 | BstC8I |  | 200 |
| TP3712_K | 200 | BstC8I | 125 | 125,75 |
| TP3712_P | 200 | Cac8I |  | 200 |
| TP3712_K | 200 | Cac8I | 125 | 125,75 |
| TP3712_P | 200 | CviJI | 171 | 171,29 |
| TP3712_K | 200 | CviJI | 123,171 | 123,48,29 |
| TP3712_P | 200 | CviKI-1 | 171 | 171,29 |
| TP3712_K | 200 | CviKI-1 | 123,171 | 123,48,29 |
| TP3712_P | 200 | DrdII | 123,189 | 123,66,11 |
| TP3712_K | 200 | DrdII | 189 | 189,11 |
| TP3712_P | 200 | Nal45188II | 125 | 125,75 |
| TP3712_K | 200 | Nal45188II |  | 200 |
| TP3712_P | 200 | Pfl8569I |  | 200 |
| TP3712_K | 200 | Pfl8569I | 125 | 125,75 |
| TP37593_P | 119 | AjuI |  | 119 |
| TP37593_K | 119 | AjuI | 80 | 80,39 |
| TP37593_P | 119 | BseGI | 61,113 | 61,52,6 |
| TP37593_K | 119 | BseGI | 113 | 113,6 |
| TP37593_P | 119 | BstF5I | 61,113 | 61,52,6 |
| TP37593_K | 119 | BstF5I | 113 | 113,6 |
| TP37593_P | 119 | BtsCI | 61,113 | 61,52,6 |
| TP37593_K | 119 | BtsCI | 113 | 113,6 |
| TP37593_P | 119 | CchII |  | 119 |
| TP37593_K | 119 | CchII | 71 | 71,48 |
| TP37593_P | 119 | EcoMVII | 59 | 60,59 |
| TP37593_K | 119 | EcoMVII |  | 119 |
| TP37593_P | 119 | FokI | 68,120 | 68,52,-1 |
| TP37593_K | 119 | FokI | 120 | 120,-1 |
| TP37593_P | 119 | HpyAXVI-mut2 | 56,108 | 56,52,11 |
| TP37593_K | 119 | HpyAXVI-mut2 | 108 | 108,11 |
| TP37593_P | 119 | HpyUM037X |  | 119 |
| TP37593_K | 119 | HpyUM037X | 55 | 64,55 |
| TP37593_P | 119 | MboII |  | 119 |
| TP37593_K | 119 | MboII | 68 | 68,51 |
| TP37593_P | 119 | PfrJS15III |  | 119 |
| TP37593_K | 119 | PfrJS15III | 56 | 63,56 |
| TP37593_P | 119 | StsI | 69,121 | 69,52,-2 |
| TP37593_K | 119 | StsI | 121 | 121,-2 |
| TP38227_P | 105 | BstMWI |  | 105 |
| TP38227_K | 105 | BstMWI | 29 | 76,29 |
| TP38227_P | 105 | CviJI | 88,102 | 88,14,3 |
| TP38227_K | 105 | CviJI | 32,88,102 | 56,32,14,3 |
| TP38227_P | 105 | CviKI-1 | 88,102 | 88,14,3 |
| TP38227_K | 105 | CviKI-1 | 32,88,102 | 56,32,14,3 |
| TP38227_P | 105 | HpyF10VI |  | 105 |
| TP38227_K | 105 | HpyF10VI | 29 | 76,29 |
| TP38227_P | 105 | LpnPI | 16,51,82 | 35,31,23,16 |
| TP38227_K | 105 | LpnPI | 16,22,51,82 | 31,29,23,16,6 |
| TP38227_P | 105 | MwoI |  | 105 |
| TP38227_K | 105 | MwoI | 29 | 76,29 |
| TP38227_P | 105 | Nal45188II |  | 105 |
| TP38227_K | 105 | Nal45188II | 34 | 71,34 |
| TP416765_P | 92 | Aco12261II | 56 | 56,36 |
| TP416765_K | 92 | Aco12261II |  | 92 |
| TP416765_P | 92 | AsuC2I |  | 92 |
| TP416765_K | 92 | AsuC2I | 56 | 56,36 |
| TP416765_P | 92 | BcnI |  | 92 |
| TP416765_K | 92 | BcnI | 56 | 56,36 |
| TP416765_P | 92 | BetI | 55 | 55,37 |
| TP416765_K | 92 | BetI |  | 92 |
| TP416765_P | 92 | Bme1390I |  | 92 |
| TP416765_K | 92 | Bme1390I | 56 | 56,36 |
| TP416765_P | 92 | BmrFI |  | 92 |
| TP416765_K | 92 | BmrFI | 56 | 56,36 |
| TP416765_P | 92 | BpuMI |  | 92 |
| TP416765_K | 92 | BpuMI | 56 | 56,36 |
| TP416765_P | 92 | BsaJI |  | 92 |
| TP416765_K | 92 | BsaJI | 54 | 54,38 |
| TP416765_P | 92 | BsaWI | 55 | 55,37 |
| TP416765_K | 92 | BsaWI |  | 92 |
| TP416765_P | 92 | BseDI |  | 92 |
| TP416765_K | 92 | BseDI | 54 | 54,38 |
| TP416765_P | 92 | BssECI |  | 92 |
| TP416765_K | 92 | BssECI | 54 | 54,38 |
| TP416765_P | 92 | BstSCI |  | 92 |
| TP416765_K | 92 | BstSCI | 54 | 54,38 |
| TP416765_P | 92 | CauII |  | 92 |
| TP416765_K | 92 | CauII | 56 | 56,36 |
| TP416765_P | 92 | EcoHI |  | 92 |
| TP416765_K | 92 | EcoHI | 54 | 54,38 |
| TP416765_P | 92 | Eli8509II | 56 | 56,36 |
| TP416765_K | 92 | Eli8509II |  | 92 |
| TP416765_P | 92 | MspR9I |  | 92 |
| TP416765_K | 92 | MspR9I | 56 | 56,36 |
| TP416765_P | 92 | NciI |  | 92 |
| TP416765_K | 92 | NciI | 56 | 56,36 |
| TP416765_P | 92 | ScrFI |  | 92 |
| TP416765_K | 92 | ScrFI | 56 | 56,36 |
| TP416765_P | 92 | SecI |  | 92 |
| TP416765_K | 92 | SecI | 54 | 54,38 |
| TP416765_P | 92 | Sth132I |  | 92 |
| TP416765_K | 92 | Sth132I | 62 | 62,30 |
| TP416765_P | 92 | StyD4I |  | 92 |
| TP416765_K | 92 | StyD4I | 54 | 54,38 |
| TP440375_P | 99 | AhaIII |  | 99 |
| TP440375_K | 99 | AhaIII | 29 | 70,29 |
| TP440375_P | 99 | DraI |  | 99 |
| TP440375_K | 99 | DraI | 29 | 70,29 |
| TP446132_P | 240 | Bst4CI | 110 | 130,110 |
| TP446132_K | 240 | Bst4CI |  | 240 |
| TP446132_P | 240 | BstC8I |  | 240 |
| TP446132_K | 240 | BstC8I | 113 | 127,113 |
| TP446132_P | 240 | BtsIMutI | 115 | 125,115 |
| TP446132_K | 240 | BtsIMutI |  | 240 |
| TP446132_P | 240 | Cac8I |  | 240 |
| TP446132_K | 240 | Cac8I | 113 | 127,113 |
| TP446132_P | 240 | CjuI | 107 | 133,107 |
| TP446132_K | 240 | CjuI |  | 240 |
| TP446132_P | 240 | HpyCH4III | 110 | 130,110 |
| TP446132_K | 240 | HpyCH4III |  | 240 |
| TP446132_P | 240 | HsoII | 127 | 127,113 |
| TP446132_K | 240 | HsoII |  | 240 |
| TP446132_P | 240 | Pfl8569I |  | 240 |
| TP446132_K | 240 | Pfl8569I | 113 | 127,113 |
| TP446132_P | 240 | TaaI | 110 | 130,110 |
| TP446132_K | 240 | TaaI |  | 240 |
| TP446132_P | 240 | TscAI | 106,115 | 125,106,9 |
| TP446132_K | 240 | TscAI |  | 240 |
| TP446132_P | 240 | Tsp4CI | 110 | 130,110 |
| TP446132_K | 240 | Tsp4CI |  | 240 |
| TP446132_P | 240 | TspRI | 106,115 | 125,106,9 |
| TP446132_K | 240 | TspRI |  | 240 |
| TP47110_P | 66 | BseMII |  | 66 |
| TP47110_K | 66 | BseMII | 38 | 38,28 |
| TP47110_P | 66 | BspCNI |  | 66 |
| TP47110_K | 66 | BspCNI | 37 | 37,29 |
| TP47110_P | 66 | BstDEI |  | 66 |
| TP47110_K | 66 | BstDEI | 24 | 42,24 |
| TP47110_P | 66 | DdeI |  | 66 |
| TP47110_K | 66 | DdeI | 24 | 42,24 |
| TP47110_P | 66 | Hpy188I |  | 66 |
| TP47110_K | 66 | Hpy188I | 27 | 39,27 |
| TP47110_P | 66 | HpyF3I |  | 66 |
| TP47110_K | 66 | HpyF3I | 24 | 42,24 |
| TP88533_P | 99 | AjnI | 68 | 68,31 |
| TP88533_K | 99 | AjnI | 51,68 | 51,31,17 |
| TP88533_P | 99 | Asp114II |  | 99 |
| TP88533_K | 99 | Asp114II | 49 | 50,49 |
| TP88533_P | 99 | AspBHI | 18,40 | 59,22,18 |
| TP88533_K | 99 | AspBHI | 18,44 | 55,26,18 |
| TP88533_P | 99 | BciT130I | 70 | 70,29 |
| TP88533_K | 99 | BciT130I | 53,70 | 53,29,17 |
| TP88533_P | 99 | Bme1390I | 70 | 70,29 |
| TP88533_K | 99 | Bme1390I | 53,70 | 53,29,17 |
| TP88533_P | 99 | BmrFI | 70 | 70,29 |
| TP88533_K | 99 | BmrFI | 53,70 | 53,29,17 |
| TP88533_P | 99 | BsaJI |  | 99 |
| TP88533_K | 99 | BsaJI | 52 | 52,47 |
| TP88533_P | 99 | BseBI | 70 | 70,29 |
| TP88533_K | 99 | BseBI | 53,70 | 53,29,17 |
| TP88533_P | 99 | BseDI |  | 99 |
| TP88533_K | 99 | BseDI | 52 | 52,47 |
| TP88533_P | 99 | BssECI |  | 99 |
| TP88533_K | 99 | BssECI | 52 | 52,47 |
| TP88533_P | 99 | Bst2UI | 70 | 70,29 |
| TP88533_K | 99 | Bst2UI | 53,70 | 53,29,17 |
| TP88533_P | 99 | BstNI | 70 | 70,29 |
| TP88533_K | 99 | BstNI | 53,70 | 53,29,17 |
| TP88533_P | 99 | BstSCI | 68 | 68,31 |
| TP88533_K | 99 | BstSCI | 51,68 | 51,31,17 |
| TP88533_P | 99 | CviAII | 52 | 52,47 |
| TP88533_K | 99 | CviAII |  | 99 |
| TP88533_P | 99 | CviJI | 76,90 | 76,14,9 |
| TP88533_K | 99 | CviJI | 51,76,90 | 51,25,14,9 |
| TP88533_P | 99 | CviKI-1 | 76,90 | 76,14,9 |
| TP88533_K | 99 | CviKI-1 | 51,76,90 | 51,25,14,9 |
| TP88533_P | 99 | Dde51507I | 70 | 70,29 |
| TP88533_K | 99 | Dde51507I | 53,70 | 53,29,17 |
| TP88533_P | 99 | EcoRII | 68 | 68,31 |
| TP88533_K | 99 | EcoRII | 51,68 | 51,31,17 |
| TP88533_P | 99 | FaeI | 55 | 55,44 |
| TP88533_K | 99 | FaeI |  | 99 |
| TP88533_P | 99 | FaiI | 26,53 | 46,27,26 |
| TP88533_K | 99 | FaiI | 26 | 73,26 |
| TP88533_P | 99 | FatI | 51 | 51,48 |
| TP88533_K | 99 | FatI |  | 99 |
| TP88533_P | 99 | Hin1II | 55 | 55,44 |
| TP88533_K | 99 | Hin1II |  | 99 |
| TP88533_P | 99 | Hsp92II | 55 | 55,44 |
| TP88533_K | 99 | Hsp92II |  | 99 |
| TP88533_P | 99 | LpnPI | 20,59,82 | 39,23,20,17 |
| TP88533_K | 99 | LpnPI | 20,42,59,65,82 | 22,20,17,17,17,6 |
| TP88533_P | 99 | MspR9I | 70 | 70,29 |
| TP88533_K | 99 | MspR9I | 53,70 | 53,29,17 |
| TP88533_P | 99 | MvaI | 70 | 70,29 |
| TP88533_K | 99 | MvaI | 53,70 | 53,29,17 |
| TP88533_P | 99 | NlaIII | 55 | 55,44 |
| TP88533_K | 99 | NlaIII |  | 99 |
| TP88533_P | 99 | Psp6I | 68 | 68,31 |
| TP88533_K | 99 | Psp6I | 51,68 | 51,31,17 |
| TP88533_P | 99 | PspGI | 68 | 68,31 |
| TP88533_K | 99 | PspGI | 51,68 | 51,31,17 |
| TP88533_P | 99 | PspPRI |  | 99 |
| TP88533_K | 99 | PspPRI | 37 | 62,37 |
| TP88533_P | 99 | ScrFI | 70 | 70,29 |
| TP88533_K | 99 | ScrFI | 53,70 | 53,29,17 |
| TP88533_P | 99 | SecI |  | 99 |
| TP88533_K | 99 | SecI | 52 | 52,47 |
| TP88533_P | 99 | SgrTI | 20,59,62,82 | 39,20,20,17,3 |
| TP88533_K | 99 | SgrTI | 20,42,59,62,65,82 | 22,20,17,17,17,3,3 |
| TP88533_P | 99 | StyD4I | 68 | 68,31 |
| TP88533_K | 99 | StyD4I | 51,68 | 51,31,17 |
| TP93026_P | 56 | AccII | 23 | 33,23 |
| TP93026_K | 56 | AccII |  | 56 |
| TP93026_P | 56 | Bsh1236I | 23 | 33,23 |
| TP93026_K | 56 | Bsh1236I |  | 56 |
| TP93026_P | 56 | BspFNI | 23 | 33,23 |
| TP93026_K | 56 | BspFNI |  | 56 |
| TP93026_P | 56 | BstFNI | 23 | 33,23 |
| TP93026_K | 56 | BstFNI |  | 56 |
| TP93026_P | 56 | BstUI | 23 | 33,23 |
| TP93026_K | 56 | BstUI |  | 56 |
| TP93026_P | 56 | CviAII |  | 56 |
| TP93026_K | 56 | CviAII | 20 | 36,20 |
| TP93026_P | 56 | FaeI |  | 56 |
| TP93026_K | 56 | FaeI | 23 | 33,23 |
| TP93026_P | 56 | FaiI | 38 | 38,18 |
| TP93026_K | 56 | FaiI | 21,38 | 21,18,17 |
| TP93026_P | 56 | FatI |  | 56 |
| TP93026_K | 56 | FatI | 19 | 37,19 |
| TP93026_P | 56 | FnuDII | 23 | 33,23 |
| TP93026_K | 56 | FnuDII |  | 56 |
| TP93026_P | 56 | Hin1II |  | 56 |
| TP93026_K | 56 | Hin1II | 23 | 33,23 |
| TP93026_P | 56 | Hsp92II |  | 56 |
| TP93026_K | 56 | Hsp92II | 23 | 33,23 |
| TP93026_P | 56 | MvnI | 23 | 33,23 |
| TP93026_K | 56 | MvnI |  | 56 |
| TP93026_P | 56 | NlaIII |  | 56 |
| TP93026_K | 56 | NlaIII | 23 | 33,23 |
| TP93026_P | 56 | PfrJS15III | 18 | 38,18 |
| TP93026_K | 56 | PfrJS15III |  | 56 |
| TP93026_P | 56 | SelI | 21 | 35,21 |
| TP93026_K | 56 | SelI |  | 56 |

# Supplementary Table S2. Parameters for linkage mapping.

| **Parameter** | **Value** |
| --- | --- |
| program | Joinmap 5.0 |
| method | maximum likelihood mapping |
| spatial sampling thresholds | 0.150, 0.045, 0.015, 0.003, 0.001 |
| sampling batch size | 50 |
| optimization rounds per sample | 4, 4, 4, 6, 6, 6 |
| chain length with constants acc. prob. | 2000, 2000, 2000, 6000, 10000, 20000 |
| stop after #chains without improvement | 2000, 2000, 2000, 6000, 10000, 20000 |
| initial acceptance probability | 0.25 |
| cooling control | 0.001 |
| length of burn-in chain | 10000 |
| Monte Carlo EM cycles | 4 |
| chain length per Monte Carlo EM cycle | 1000 |

# Supplementary Table S3. Parameters for QTL mapping.

| **Program** | **MapQTL6** |
| --- | --- |
| method | interval mapping |
| algorithm | mixture model |
| mapping step size | 0.1 |
| test statistic | LOD |
| maximum no. of iterations | 200 |
| functional tolerance | 1.0e-08 |
| population type | RI8 |
| dominance fitted | no |
| QTL genotype probability approximation | no |
| maximum no. of neighboring markers used | 10 |
| **Program** | **Windows QTL Cartographer V2.5** |
| method | composite interval mapping |
| algorithm | backward regression |
| walk speed | 0.5 cM |
| test statistic | likelihood ratio (LR) |
| model | 6 standard model |
| control marker number | 20 |
| window size | 10 cM |

# Supplementary Table S4. The list of white lupin lines used for anthracnose resistance marker validation.

| **Field**  **No.** | **Accession** | **Name** | **Country** | **Type** |
| --- | --- | --- | --- | --- |
| 1 | 95015 | San Felices | Spain | primitive population |
| 3 | 95038 | Population-8018 | Spain | primitive population |
| 4 | 95041 | Population-8021 | Spain | primitive population |
| 6 | 95047 | Population-8030 | Spain | primitive population |
| 7 | 95077 | Population B-297/79 | Spain | primitive population |
| 9 | 95080 | Population B-2007/80 | Spain | primitive population |
| 10 | 95101 | Calabria | Italy | primitive population |
| 11 | 95109 | Population-84003 | Spain | primitive population |
| 12 | 95113 | LA-108 | France | landrace |
| 13 | 95114 | LA-109 | France | landrace |
| 14 | 95130 | Population-84152 | Spain | landrace |
| 15 | 95132 | Tremoco Gorzki | Portugal | landrace |
| 17 | 95143 | Population-199 | Spain | primitive population |
| 18 | 95146 | AL-19 | Spain | landrace |
| 19 | 95149 | AL-22 | Spain | landrace |
| 20 | 95151 | AL-26 | Spain | landrace |
| 21 | 95152 | AL-27 | Spain | landrace |
| 22 | 95155 | R-32 | Spain | landrace |
| 23 | 95177 | Mutant Self Completing | Poland | mutant |
| 24 | 95187 | BGRC-3911 | Italy | primitive population |
| 25 | 95211 | Turmus balady gadim,Druze L.R. | Israel | landrace |
| 26 | 95212 | Turmus b. g. L.R.M.-2738 | Jordan | landrace |
| 27 | 95213 | Turmus balady L.R.M.-2675 | Jordan | landrace |
| 28 | 95216 | L-229-N | Republic of South Africa | primitive population |
| 29 | 95219 | Giza-2 | Egypt | landrace |
| 30 | 95223 | TR-5 | Turkey | primitive population |
| 31 | 95234 | P.21525 | Chile | landrace |
| 32 | 95243 | Pop.2023 | Algeria | primitive population |
| 33 | 95244 | Pop. 2027 | Algeria | primitive population |
| 34 | 95245 | Lupini Bean | United Kingdom | primitive population |
| 35 | 95246 | Pop.2078 | Ethiopia | primitive population |
| 36 | 95267 | Lobbi Barcelona | Spain | primitive population |
| 37 | 95276 | Siebacher Red BGR 6321 | Portugal | cultivar |
| 38 | 95406 | R-933 | Poland | cross derivative |
| 39 | 95407 | Tremoco Doce | Portugal | landrace |
| 40 | 95409 | Neutra | Germany | cultivar |
| 43 | 95422 | Start | Russia | cultivar |
| 44 | 95424 | Kali x Laju Biały-1 | Poland | cross derivative |
| 45 | 95427 | Sel.ecot. x LA-109/1 | Spain | cross derivative |
| 46 | 95428 | Pflugs Gela | Germany | cultivar |
| 47 | 95440 | HetmanxStart | Poland | cross derivative |
| 48 | 95468 | Azurnyj | Belarus | landrace |
| 49 | 95479 | Kijewskij Mutant | Russia | cultivar |
| 50 | 95480 | Nelly | Hungary | cultivar |
| 53 | 95513 | Butan | Poland | cultivar |
| 58 | 95523 | FRA6708B | Turkey | primitive population |
| 59 | 95524 | FRA6712B | Turkey | primitive population |
| 60 | 95525 | FRA6713B | Turkey | primitive population |
| 61 | 95526 | LA359 | France | primitive population |
| 62 | 95527 | LA382 | Egypt | primitive population |
| 63 | 95528 | LA384 | Egypt | primitive population |
| 64 | 95602 | Population-775 | Greece | primitive population |
| 65 | 95605 | Population-743 | Greece | primitive population |
| 66 | 95482 | Pikador | Poland | cultivar |
| 68 | 95516 | MJS208-2 | Spain | primitive population |
| 69 | 95517 | Damascus | Syrian Arab Republic | primitive population |
| 70 | 95522 | FRA6706B | Turkey | primitive population |
| 71 | 95529 | LA423 | Sudan | primitive population |
| 72 | 95530 | Boros | Poland | cultivar |
| 74 | 95419 | Kalina | Poland | cultivar |
| 75 | 95403 | Wat | Poland | cultivar |
| 76 | 95481 | Bardo | Poland | cultivar |
| 77 | 95036 | Population-8011 | Spain | landrace |
| 78 | 95072 | Population B-267/79 | Spain | primitive population |
| 79 | 95075 | Population B-295/79 | Spain | primitive population |
| 80 | 95163 | SF-387 | Spain | landrace |
| 81 | 95442 | Mutant SelfCompleting x Nahrq. | Poland | cross derivative |
| 82 | 95048 | Population-8031 | Spain | primitive population |
| 83 | 95064 | Population-8062 | Spain | primitive population |
| 84 | 95076 | Population B-296/76 | Spain | primitive population |
| 85 | 95084 | El Harrach-1 | Algeria | landrace |
| 86 | 95102 | LA-31 San Felices | Spain | primitive population |
| 87 | 95109 | Population-84003 | Spain | primitive population |
| 88 | 95145 | AL-18 | Spain | landrace |
| 89 | 95149 | AL-22 | Spain | landrace |
| 90 | 95470 | R6902 x El Harrach 2 | Poland | cross derivative |
| 91 | 95054 | Population-8039 | Spain | primitive population |
| 92 | 95059 | Population-8053 | Spain | primitive population |
| 93 | 95237 | L. graecus x Mut.Topless x Hetman F | Poland | cross derivative |
| 94 | 95125 | Population-877 | Greece | primitive population |
| 95 | 95147 | AL-20 | Spain | landrace |
| 96 | 95151 | AL-26 | Spain | landrace |
| 97 | 95033 | Population-8006 | Spain | primitive population |
| 98 | 95035 | Population-8010 | Spain | primitive population |
| 99 | 95042 | Population-8022 | Spain | primitive population |
| 100 | 95078 | Population B-300/79 | Spain | primitive population |
| 101 | 95097 | Tremoco | Portugal | landrace |
| 102 | 95124 | Population-780 | Greece | primitive population |
| 103 | 95144 | M.Mut.z Hiszp.(topless) | Spain | mutant |
| 104 | 95153 | AL-28 | Spain | landrace |
| 105 | 95175 | R-84141 | Spain | landrace |
| 106 | 95148 | AL-21 | Spain | landrace |
| 107 | 95476 | Athos | France | cultivar |
| 108 | 95049 | Population-8032 | Spain | primitive population |
| 109 | 95116 | Mutant cienkoscienny | Spain | mutant |
| 110 | 95071 | Population B-266/79 | Spain | primitive population |
| 111 | 95157 | R-114 | Spain | landrace |
| 112 | 95103 | LA-20 | Spain | landrace |
| 113 | 95038 | Population-8018 | Spain | primitive population |
| 114 | 95609 | P.26780 | Greece | primitive population |
| 115 | 95108 | Population-84002 | Spain | primitive population |
| 116 | 95040 | Population-8020 | Spain | primitive population |
| 117 | 95047 | Population-8030 | Spain | primitive population |
| 132 | 95434 | Lotos | Russia | cultivar |
| 133 | 95510 | Hansa (95510) | Germany | cultivar |
| D | - | P27174 | Ethiopia | landrace |
| K | - | Kiev Mutant | Ukraine | cultivar |

# Supplementary Table S5. The summary of transformation of white lupin GBS-derived markers localized in anthracnose resistance loci to PCR-based markers.

| **QTL name** | **Marker name** | **Analyzed** | **Transcript assigned** | **Possibility of direct transformation to PCR marker** | **Possibility of transformation to PCR marker using transcript** | **Attempt to PCR marker development** |
| --- | --- | --- | --- | --- | --- | --- |
| antr04_1, antr05_1 | TP23903 | yes | no | yes | - | yes |
| antr04_1, antr05_1 | TP229924 | yes | yes | no | yes | yes |
| antr04_1, antr05_1 | TP222136 | yes | yes | no | yes | yes |
| antr04_1, antr05_1 | WANR1 | yes | - | - | - | no |
| antr04_1, antr05_1 | TP24947 | yes | yes | no | no | no |
| antr04_1, antr05_1 | TP272531 | yes | yes | yes | yes | yes |
| antr04_1, antr05_1 | TP94529 | yes | no | no | - | no |
| antr04_1, antr05_1 | TP135791 | yes | no | no | - | no |
| antr04_1, antr05_1 | TP254603 | yes | yes | no | yes | yes |
| antr04_1, antr05_1 | TP47110 | yes | yes | yes | yes | yes |
| antr04_1, antr05_1 | TP458 | yes | no | no | - | no |
| antr04_1, antr05_1 | TP2471 | yes | no | no | - | no |
| antr04_1, antr05_1 | TP291372 | yes | yes | no | yes | yes |
| antr04_1, antr05_1 | TP323017 | yes | no | no | - | no |
| antr04_1, antr05_1 | TP236266 | yes | no | no | - | no |
| antr04_1, antr05_1 | TP13313 | yes | no | no | - | no |
| antr04_1, antr05_1 | TP231214 | yes | yes | no | no | no |
| antr04_1, antr05_1 | TP446132 | yes | yes | no | yes | yes |
| antr04_1, antr05_1 | TP237794 | yes | yes | yes | yes | yes |
| antr04_1, antr05_1 | TP38227 | yes | yes | no | yes | yes |
| antr04_1, antr05_1 | TP88533 | yes | yes | no | yes | yes |
| antr04_1, antr05_1 | TP152838 | yes | yes | no | yes | no |
| antr04_1, antr05_1 | TP68244 | yes | yes | no | yes | no |
| antr04_2, antr05_2 | TP270602 | yes | no | no | - | no |
| antr04_2, antr05_2 | TP154225 | yes | no | no | - | no |
| antr04_2, antr05_2 | TP89744 | yes | no | no | - | no |
| antr04_2, antr05_2 | TP149038 | yes | yes | yes | yes | yes |
| antr04_2, antr05_2 | TP416765 | yes | yes | yes | yes | yes |
| antr04_2, antr05_2 | TP272081 | yes | no | yes | yes | yes |
| antr04_2, antr05_2 | TP345661 | yes | no | no | - | no |
| antr04_2, antr05_2 | TP432453 | yes | yes | no | no | no |
| antr04_2, antr05_2 | WANR3 | yes | - | - | - | no |
| antr04_2, antr05_2 | lPms-517067 | no | - | - | - | no |
| antr04_2, antr05_2 | lPms-524687 | no | - | - | - | no |
| antr04_2, antr05_2 | TP444157 | yes | no | no | - | no |
| antr04_2, antr05_2 | TP73498 | yes | no | no | - | no |
| antr04_2, antr05_2 | LG96 | no | - | - | - | no |
| antr04_2, antr05_2 | TP3712 | yes | yes | no | yes | yes |
| antr04_2, antr05_2 | TP364001 | yes | yes | yes | yes | yes |
| antr04_2, antr05_2 | TP327093 | yes | yes | no | yes | yes |
| antr04_2, antr05_2 | TP277222 | yes | no | no | - | no |
| antr04_2, antr05_2 | TP26007 | yes | yes | no | - | yes |
| antr04_2, antr05_2 | TP37593 | yes | yes | no | - | yes |
| antr04_2, antr05_2 | TP338317 | yes | yes | no | yes | no |
| antr04_2, antr05_2 | TP106254 | yes | yes | no | yes | yes |
| antr04_2, antr05_2 | TP93026 | yes | no | yes | - | yes |
| antr04_2, antr05_2 | TP287341 | yes | no | no | - | no |
| antr04_2, antr05_2 | TP440375 | yes | yes | no | yes | yes |
| antr04_2, antr05_2 | TP338761 | yes | yes | yes | yes | yes |
| antr04_2, antr05_2 | TP9255 | yes | yes | no | no | no |
| antr04_2, antr05_2 | lPms-527184 | no | - | - | - | no |

# Supplementary Table S6. The list of white lupin transcriptome sequences anchored to white lupin GBS marker sequences with alignment data.

P, line P27174; K, Kiev Mutant.

| **GBS read (query)** | **Query coverage %** | **Transcript (hit)** | **Hit start nt** | **Hit end nt** | **Pairwise Identity %** | **Bit-Score** | **E Value** |
| --- | --- | --- | --- | --- | --- | --- | --- |
| TP106254_P | 100% | P27174_comp40075_c0_seq1 | 390 | 327 | 100% | 117 | 3.E-26 |
| TP149038_P | 100% | P27174_comp54298_c0_seq1 | 103 | 40 | 100% | 117 | 3.E-26 |
| TP222136_K | 100% | LAGI01_42695 | 1249 | 1312 | 100% | 117 | 4.E-26 |
| TP229924_K | 100% | Kiev_comp451_c0_seq1 | 205 | 142 | 100% | 117 | 2.E-26 |
| TP237794_K | 100% | LAGI01_1593 | 1693 | 1756 | 100% | 117 | 4.E-26 |
| TP23903_K | - | - | - | - | - | - | - |
| TP254603_K | 100% | LAGI01_37098 | 1042 | 979 | 100% | 117 | 4.E-26 |
| TP26007_K | 100% | LAGI01_59788 | 375 | 312 | 100% | 117 | 4.E-26 |
| TP272081_K | - | - | - | - | - | - | - |
| TP272531_K | 100% | LAGI01_27710 | 637 | 574 | 100% | 117 | 4.E-26 |
| TP291372_K | 77% | LAGI01_24193 | 1147 | 1195 | 100% | 90 | 6.E-18 |
| TP327093_K | 100% | LAGI01_15083 | 854 | 917 | 100% | 117 | 4.E-26 |
| TP338761_K | 100% | LAGI01_64257 | 116 | 53 | 100% | 117 | 4.E-26 |
| TP364001_K | 100% | LAGI01_21661 | 1577 | 1640 | 100% | 117 | 4.E-26 |
| TP3712_K | 100% | LAGI01_21661 | 1663 | 1600 | 100% | 117 | 4.E-26 |
| TP37593_K | 100% | LAGI01_93174 | 115 | 52 | 100% | 117 | 4.E-26 |
| TP38227_K | 100% | LAGI01_20906 | 1782 | 1719 | 100% | 117 | 4.E-26 |
| TP416765_K | 100% | LAGI01_10703 | 270 | 207 | 100% | 117 | 4.E-26 |
| TP440375_K | 100% | LAGI01_45536 | 1140 | 1203 | 100% | 117 | 4.E-26 |
| TP446132_K | 100% | LAGI01_1593 | 2802 | 2739 | 100% | 117 | 4.E-26 |
| TP47110_K | 100% | LAGI01_37072 | 445 | 508 | 100% | 117 | 4.E-26 |
| TP88533_K | 100% | LAGI01_20906 | 1673 | 1736 | 100% | 117 | 4.E-26 |
| TP93026_K | - | - | - | - | - | - | - |

# Supplementary Table S7. The list of narrow-leafed lupin genome sequences anchored to designed white lupin PCR marker sequences with alignment data.

| **PCR marker (query)** | **Query coverage %** | **Chromosome (hit)** | **Hit start nt** | **Hit end nt** | **Pairwise Identity %** | **Bit-Score** | **E Value** |
| --- | --- | --- | --- | --- | --- | --- | --- |
| TP338761 | 100% | NLL-02 | 8904913 | 8904803 | 96% | 178 | 1.E-43 |
| TP364001 | 100% | NLL-02 | 5415942 | 5415750 | 92% | 281 | 3.E-74 |
| TP440375 | 100% | NLL-02 | 9939893 | 9939990 | 90% | 131 | 1.E-29 |
| TP3712 | 100% | NLL-02 | 5415743 | 5415942 | 92% | 290 | 6.E-77 |
| TP26007 | 100% | NLL-02 | 5935771 | 5935877 | 96% | 176 | 4.E-43 |
| TP37593 | 100% | NLL-02 | 11383664 | 11383798 | 85% | 160 | 4.E-38 |
| TP93026 | 93% | NLL-02 | 10741580 | 10741529 | 85% | 59 | 3.E-08 |
| TP106254 | 100% | NLL-02 | 11998673 | 11998558 | 91% | 162 | 1.E-38 |
| TP149038 | 99% | NLL-02 | 4773789 | 4773887 | 94% | 153 | 5.E-36 |
| TP222136 | 73% | NLL-04 | 26705333 | 26705484 | 85% | 167 | 5.E-40 |
| TP291372 | 100% | NLL-08 | 9128142 | 9128038 | 98% | 182 | 1.E-44 |
| TP416765 | 97% | NLL-11 | 21630677 | 21630589 | 88% | 113 | 4.E-24 |
| TP272081 | 88% | NLL-13 | 15443784 | 15443839 | 96% | 93 | 2.E-18 |
| TP327093 | 76% | NLL-13 | 16001072 | 16000996 | 75% | 54 | 3.E-06 |
| TP272531 | 100% | NLL-20 | 684868 | 684708 | 97% | 268 | 2.E-70 |
| TP446132 | 100% | NLL-20 | 1194191 | 1193952 | 98% | 407 | 4.E-112 |
| TP23903 | 100% | NLL-20 | 189188 | 189251 | 92% | 93 | 2.E-18 |
| TP38227 | 99% | NLL-20 | 1377983 | 1377880 | 96% | 171 | 2.E-41 |
| TP47110 | 100% | NLL-20 | 696253 | 696188 | 100% | 120 | 1.E-26 |
| TP88533 | 100% | NLL-20 | 1377806 | 1377904 | 95% | 156 | 4.E-37 |
| TP229924 | 85% | NLL-20 | 535379 | 535307 | 81% | 66 | 4.E-10 |
| TP237794 | 100% | NLL-20 | 1193008 | 1193073 | 94% | 102 | 4.E-21 |
| TP254603 | 100% | NLL-20 | 695664 | 695764 | 97% | 169 | 6.E-41 |

# Supplementary Table S8. Updated marker segregation data for white lupin RIL population.

| **Marker** | **WANR1a** | **TP23903a** | **TP229924a** | **TP222136a** | **TP272531a** | **TP47110a** | **TP291372a** | **TP446132a** | **TP237794a** | **TP38227a** | **TP88533a** | **WANR3a** | **TP149038a** | **TP416765a** | **TP3712a** | **TP364001a** | **TP26007a** | **TP37593a** | **TP106254a** | **TP93026a** | **TP440375a** | **TP338761a** |
| --- | --- | --- | --- | --- | --- | --- | --- | --- | --- | --- | --- | --- | --- | --- | --- | --- | --- | --- | --- | --- | --- | --- |
| RIL-1 | a | a | a | a | a | a | a | a | a | a | a | b | b | b | b | b | b | b | b | b | b | b |
| RIL-2 | - | - | - | - | - | - | - | - | - | - | - | b | - | - | - | - | - | - | - | - | - | - |
| RIL-3 | a | a | a | a | a | a | a | a | a | b | b | b | b | b | b | b | b | b | b | b | b | b |
| RIL-4 | b | b | b | b | b | b | b | b | b | b | b | a | a | a | a | a | a | a | a | a | a | a |
| RIL-5 | b | b | b | b | b | b | b | b | b | b | b | a | a | a | a | a | b | b | b | b | b | b |
| RIL-6 | b | b | b | b | b | b | b | b | b | b | b | a | a | a | a | a | a | a | a | a | a | a |
| RIL-7 | a | a | a | a | a | a | a | a | a | a | a | a | a | a | a | a | a | a | a | a | a | a |
| RIL-8 | b | b | b | b | b | b | b | b | b | b | b | b | a | b | b | b | b | b | b | b | b | b |
| RIL-9 | a | a | a | a | a | a | a | a | a | a | a | b | b | b | b | b | b | b | b | b | b | b |
| RIL-10 | b | a | b | b | b | b | b | b | b | b | b | a | a | a | a | a | a | a | a | a | a | a |
| RIL-11 | a | a | a | a | a | a | a | a | a | a | a | b | b | b | b | b | b | b | b | b | b | b |
| RIL-12 | a | a | a | a | a | a | a | a | a | a | a | a | a | a | a | a | b | b | b | b | b | b |
| RIL-13 | b | b | b | b | b | b | b | b | b | b | b | b | b | b | b | b | b | b | b | b | b | b |
| RIL-14 | b | b | b | b | b | b | b | b | b | b | b | a | a | a | b | b | b | b | b | b | b | b |
| RIL-15 | b | b | b | b | b | b | b | b | b | b | b | a | a | a | b | b | b | b | b | b | b | b |
| RIL-16 | a | a | a | a | a | a | a | a | a | a | a | a | a | a | a | a | a | a | a | a | a | a |
| RIL-18 | b | b | b | b | b | b | b | b | b | b | b | a | a | a | a | a | a | a | a | a | a | a |
| RIL-19 | b | b | b | b | b | b | b | b | b | b | b | a | a | a | a | a | a | a | a | a | a | a |
| RIL-20 | a | a | a | a | a | a | a | a | a | a | a | b | b | b | b | b | b | b | b | b | b | b |
| RIL-21 | b | b | b | b | b | b | b | b | b | b | b | b | b | b | b | b | b | b | b | b | b | b |
| RIL-22 | a | a | a | a | a | a | a | a | a | a | a | b | b | b | b | b | b | b | b | b | b | b |
| RIL-23 | b | b | b | b | b | b | b | b | b | b | b | a | a | a | a | a | a | a | a | a | a | a |
| RIL-25 | a | a | a | a | a | a | a | a | a | a | a | b | b | b | b | b | b | b | b | b | b | b |
| RIL-26 | a | a | a | a | a | a | a | a | a | a | a | b | b | b | b | b | b | b | b | b | b | b |
| RIL-27 | b | b | b | b | b | b | b | b | b | b | b | a | a | a | a | a | a | a | a | a | a | a |
| RIL-28 | a | a | a | a | a | a | a | a | a | a | a | a | a | a | a | a | a | a | a | a | a | a |
| RIL-29 | b | b | b | b | b | b | b | b | b | b | b | a | a | a | a | a | a | a | a | a | a | a |
| RIL-30 | b | b | b | b | b | b | b | b | b | b | b | a | a | a | a | a | a | a | a | a | a | a |
| RIL-31 | b | b | b | b | b | b | b | b | b | b | b | b | b | b | b | b | b | b | b | b | b | b |
| RIL-32 | b | b | b | b | b | b | b | b | b | b | b | b | b | b | b | b | b | b | b | b | b | b |
| RIL-33 | a | a | a | a | a | a | a | a | a | a | a | a | a | a | a | a | a | a | a | a | a | a |
| RIL-34 | b | b | b | b | b | b | b | b | b | b | b | b | b | b | b | b | b | b | b | b | b | b |
| RIL-35 | a | a | a | a | a | a | a | a | a | a | a | a | a | a | a | a | a | a | a | a | a | a |
| RIL-36 | b | b | b | b | b | b | b | b | b | b | b | a | b | a | a | a | a | a | a | a | a | a |
| RIL-37 | b | b | b | b | b | b | b | b | b | b | b | a | a | a | a | a | a | a | a | a | a | a |
| RIL-38 | a | a | a | a | a | a | a | a | a | a | a | b | b | b | b | b | b | b | b | b | b | b |
| RIL-39 | a | a | a | a | a | a | a | a | a | a | a | b | b | b | b | b | b | b | b | b | b | b |
| RIL-40 | b | b | b | b | b | b | a | a | a | a | a | a | a | a | a | a | a | a | a | a | a | a |
| RIL-41 | b | a | b | b | b | b | b | b | b | b | b | b | b | b | b | b | a | a | a | a | a | a |
| RIL-42 | b | b | b | b | b | b | b | b | b | b | b | b | b | b | b | b | b | b | b | b | b | b |
| RIL-43 | b | b | b | b | b | h | a | a | a | a | a | b | b | b | b | b | b | b | b | b | b | b |
| RIL-44 | a | a | a | a | a | a | a | a | a | a | a | b | b | b | b | b | b | b | b | b | b | b |
| RIL-45 | a | a | a | a | a | a | a | a | a | a | a | b | b | b | b | b | b | b | b | b | b | b |
| RIL-46 | b | b | b | b | b | b | b | b | b | b | b | b | - | b | b | b | b | b | b | b | b | b |
| RIL-47 | a | a | a | a | a | a | a | a | a | a | a | a | a | a | a | a | a | a | a | a | a | a |
| RIL-48 | b | b | b | b | b | b | b | b | b | b | b | a | a | a | a | a | a | a | a | a | a | a |
| RIL-49 | a | a | a | a | a | a | a | a | a | a | a | a | a | a | - | a | a | a | a | a | a | b |
| RIL-50 | a | a | a | a | a | a | a | a | a | a | a | a | a | a | a | a | a | a | a | a | a | a |
| RIL-51 | b | b | b | b | b | b | b | b | b | b | b | b | b | b | b | b | b | b | b | b | b | b |
| RIL-52 | b | b | b | b | b | b | b | b | b | b | b | b | b | b | b | b | a | a | a | a | a | a |
| RIL-53 | b | b | b | b | b | b | b | b | b | b | b | b | b | b | b | b | b | b | b | b | b | b |
| RIL-54 | b | b | b | b | b | b | b | b | b | b | b | b | b | b | b | b | b | b | b | b | b | b |
| RIL-55 | b | b | b | b | b | b | b | b | b | b | b | b | b | b | b | b | b | b | b | b | b | b |
| RIL-56 | b | b | b | b | b | b | b | b | b | b | b | a | a | a | a | a | a | a | a | b | b | b |
| RIL-57 | a | a | a | a | a | a | a | a | a | a | a | a | a | a | a | a | a | a | b | b | b | b |
| RIL-58 | b | b | b | b | b | b | b | b | b | b | b | b | b | b | b | b | b | b | b | b | b | b |
| RIL-59 | b | b | b | b | b | b | b | b | b | b | b | b | b | b | b | b | b | b | b | b | b | b |
| RIL-60 | b | b | b | b | b | b | b | b | b | b | b | a | a | a | a | a | a | a | a | a | a | a |
| RIL-61 | b | b | b | b | b | b | b | b | b | b | b | a | a | a | a | a | a | a | a | a | a | a |
| RIL-62 | a | a | a | a | a | a | a | a | a | a | a | - | a | b | b | b | b | b | b | b | b | b |
| RIL-63 | b | b | b | b | b | b | b | b | b | b | b | a | a | a | a | a | a | a | a | a | a | a |
| RIL-64 | a | a | a | a | a | a | a | a | a | a | a | a | b | a | a | a | a | a | a | a | a | a |
| RIL-65 | b | b | b | b | b | b | b | b | b | b | b | b | b | b | b | b | b | b | b | a | a | a |
| RIL-66 | b | b | b | b | b | b | b | b | b | b | b | a | a | a | a | a | a | a | a | a | a | a |
| RIL-67 | a | a | a | a | a | a | a | a | a | a | a | b | b | b | b | b | b | b | b | b | b | b |
| RIL-68 | a | a | a | a | a | a | a | a | a | b | b | a | a | a | a | a | a | a | a | a | a | a |
| RIL-69 | b | b | b | b | b | b | b | b | b | b | b | a | a | a | a | a | a | a | a | a | b | b |
| RIL-70 | b | b | b | b | b | b | b | b | b | b | b | b | b | b | b | b | b | b | b | b | b | b |
| RIL-71 | a | a | a | a | a | a | a | a | a | a | a | a | a | a | a | a | a | a | a | a | a | a |
| RIL-72 | a | a | a | b | b | b | b | b | b | b | b | a | a | a | a | a | a | a | a | a | a | a |
| RIL-73 | b | b | b | b | b | b | b | b | b | b | b | a | a | a | a | a | a | a | a | a | a | a |
| RIL-74 | a | a | b | a | a | a | a | a | a | a | a | a | a | a | a | a | a | a | a | a | a | a |
| RIL-75 | b | b | b | b | b | b | b | b | b | b | b | b | b | b | b | b | b | b | b | b | b | b |
| RIL-76 | b | b | b | b | b | b | b | b | b | b | b | b | b | b | b | b | b | b | b | b | b | b |
| RIL-77 | a | a | a | a | a | a | a | a | a | a | a | b | a | b | b | b | b | b | b | b | b | b |
| RIL-78 | b | b | b | b | b | b | a | a | a | a | a | b | b | b | b | b | b | b | b | b | b | b |
| RIL-79 | a | a | a | a | a | a | a | b | b | b | b | b | b | b | b | b | b | b | b | b | b | b |
| RIL-80 | a | a | a | a | a | a | a | a | a | a | a | b | b | b | b | b | b | b | a | a | a | a |
| RIL-81 | b | b | b | b | b | b | b | b | b | b | b | b | b | b | b | b | b | b | b | b | b | b |
| RIL-82 | b | b | b | b | b | b | b | b | b | b | b | a | a | a | a | a | a | a | a | a | a | a |
| RIL-83 | b | b | b | b | b | b | b | b | b | b | b | a | a | a | a | a | b | b | b | b | b | b |
| RIL-84 | a | a | a | a | a | a | a | a | a | a | a | a | a | a | a | a | a | a | a | a | a | a |
| RIL-86 | b | b | b | b | b | a | a | a | a | b | b | b | b | b | b | b | b | b | b | b | b | b |
| RIL-87 | b | b | b | b | b | b | b | b | b | b | b | a | a | a | a | a | a | a | a | a | a | a |
| RIL-88 | a | a | a | a | a | a | a | a | a | a | a | b | b | b | b | b | b | b | b | b | b | b |
| RIL-89 | a | a | a | a | a | a | a | a | a | a | a | a | a | a | a | - | a | a | a | a | a | a |
| RIL-90 | b | b | b | b | b | b | b | b | b | b | b | b | b | b | b | b | b | b | b | b | b | b |
| RIL-91 | a | a | a | a | a | a | a | a | a | a | a | a | a | a | a | a | a | a | a | a | a | a |
| RIL-92 | b | b | b | b | b | b | b | b | b | b | b | a | a | a | a | a | a | a | a | a | a | a |
| RIL-93 | b | b | b | b | b | b | a | a | a | a | a | a | a | a | a | a | a | a | a | a | a | a |
| RIL-94 | a | a | a | a | a | a | a | a | a | a | a | a | a | a | a | a | a | a | a | a | a | a |
| RIL-96 | a | a | a | a | a | a | a | a | a | a | a | a | a | a | a | a | a | a | a | a | a | a |
| RIL-97 | b | b | b | b | b | b | b | b | b | b | b | b | b | b | b | b | a | a | a | a | a | a |
| RIL-98 | b | b | b | b | b | b | b | b | b | b | b | a | a | a | a | a | a | a | a | a | a | a |
| RIL-99 | b | a | b | b | b | b | a | a | a | a | a | b | b | b | b | b | b | b | b | b | b | b |
| RIL-100 | b | b | b | b | b | b | b | b | b | b | b | a | a | a | a | a | a | a | a | a | a | a |
| RIL-101 | a | a | a | a | a | a | a | a | a | a | a | a | a | a | a | a | a | a | a | a | a | a |
| RIL-102 | b | b | b | b | b | b | b | b | b | b | b | b | b | b | b | b | b | b | b | b | b | b |
| RIL-103 | a | a | a | a | a | a | a | a | a | a | a | a | a | a | a | a | a | a | a | a | a | a |
| RIL-104 | a | a | a | a | a | a | a | a | a | a | a | b | b | b | b | b | b | b | b | b | b | b |
| RIL-105 | b | b | b | b | b | b | b | b | b | b | b | b | b | b | b | b | b | b | b | b | b | b |
| RIL-106 | b | b | b | b | b | b | b | b | b | b | b | a | a | a | a | a | a | a | a | a | a | a |
| RIL-107 | a | a | a | a | a | a | a | a | a | a | a | a | a | a | a | a | a | a | a | a | a | a |
| RIL-108 | b | b | b | b | b | b | b | b | b | b | b | b | b | b | b | b | b | b | b | b | b | b |
| RIL-109 | a | a | a | a | a | a | a | a | a | a | a | a | a | a | a | a | a | a | a | a | a | a |
| RIL-110 | b | b | b | b | b | b | b | b | b | b | b | b | b | b | b | b | b | b | b | b | b | b |
| RIL-111 | a | a | a | a | a | a | a | a | a | a | a | b | b | b | b | b | b | b | b | b | b | b |
| RIL-112 | b | b | b | b | b | b | b | b | b | b | b | a | a | a | a | a | a | a | a | a | a | a |
| RIL-113 | b | b | b | b | b | b | b | b | b | b | b | a | a | a | a | a | a | a | a | a | a | a |
| RIL-114 | b | b | b | b | b | b | b | b | b | b | b | b | b | b | b | b | b | b | b | b | b | b |
| RIL-115 | b | b | b | b | b | b | b | b | b | b | b | b | a | b | b | b | b | b | b | b | b | b |
| RIL-117 | a | a | a | a | a | a | a | a | a | a | a | b | h | b | b | b | b | b | b | b | a | b |
| RIL-118 | a | a | a | a | a | a | a | a | a | a | a | b | b | b | b | b | b | b | b | b | b | b |
| RIL-119 | a | a | a | a | a | a | a | a | a | a | a | b | b | a | b | b | b | b | b | b | a | b |
| RIL-120 | b | b | b | b | b | b | b | b | b | b | b | a | a | a | a | a | a | a | a | a | a | a |
| RIL-121 | b | b | b | b | b | b | b | b | b | b | b | b | b | b | b | b | b | b | b | b | b | b |
| RIL-122 | b | b | b | b | b | b | b | b | b | b | b | a | a | a | a | a | a | a | a | a | a | a |
| RIL-123 | a | b | a | a | a | a | b | b | b | b | b | a | b | a | a | a | a | a | a | a | a | a |
| RIL-124 | b | b | b | b | b | b | b | b | b | b | b | a | a | a | a | a | a | a | a | a | a | a |
| RIL-125 | a | a | a | a | a | a | a | a | a | a | a | b | b | b | b | b | b | b | b | b | b | b |
| RIL-126 | a | a | a | a | a | a | a | a | a | a | a | b | b | b | b | b | b | b | b | b | b | b |
| RIL-127 | b | b | b | b | b | b | b | b | b | b | b | b | b | b | b | b | b | b | b | b | b | b |
| RIL-128 | a | a | a | a | a | a | a | a | a | a | a | b | b | b | b | b | b | b | b | b | b | b |
| RIL-129 | a | b | a | a | a | a | a | a | a | a | a | a | a | a | a | a | a | a | b | b | b | b |
| RIL-130 | b | b | b | b | b | b | b | b | b | b | b | a | a | a | a | a | a | a | a | a | a | a |
| RIL-131 | b | b | b | b | b | b | b | b | b | b | b | a | a | a | a | a | a | a | a | a | a | a |
| RIL-132 | b | b | - | b | b | b | b | a | a | a | a | a | a | a | a | a | a | a | a | a | a | a |
| RIL-134 | a | a | a | a | a | a | a | a | a | a | a | a | a | a | a | a | a | a | a | a | a | a |
| RIL-135 | b | b | b | b | b | b | b | b | b | a | b | b | b | b | b | b | b | b | b | b | b | b |
| RIL-136 | b | b | b | b | b | b | b | b | b | b | b | b | b | b | b | - | b | b | b | b | b | b |
| RIL-137 | b | b | b | b | b | b | b | b | b | b | b | a | - | a | a | a | a | a | a | b | b | b |
| RIL-138 | a | a | a | a | a | a | a | a | a | a | a | a | a | a | a | a | a | a | a | a | a | a |
| RIL-139 | a | a | a | a | a | a | a | a | a | a | a | b | b | b | b | b | b | b | b | b | b | b |
| RIL-140 | b | b | b | b | b | b | b | b | b | b | b | a | a | a | a | a | a | a | a | a | a | a |
| RIL-141 | a | a | a | a | a | a | a | a | a | a | a | b | b | b | b | b | b | b | b | b | b | b |
| RIL-142 | b | b | b | b | b | b | b | b | b | b | b | b | b | b | b | b | b | b | b | b | b | b |
| RIL-143 | b | b | b | b | b | b | b | b | b | b | b | a | a | a | a | a | a | a | a | a | a | a |
| RIL-144 | b | - | b | b | b | b | b | b | b | b | b | b | b | b | b | b | b | b | b | b | b | b |
| RIL-145 | a | a | a | a | a | a | a | a | a | a | a | b | b | b | b | - | b | b | b | b | b | b |
| RIL-146 | b | b | b | b | b | b | b | b | b | b | b | b | b | b | b | b | b | b | b | b | b | b |
| RIL-147 | b | b | b | b | b | b | b | b | b | b | b | a | a | a | a | a | a | a | a | a | a | a |
| RIL-148 | b | b | b | b | b | b | b | b | b | b | b | b | b | b | b | b | b | b | b | b | b | b |
| RIL-149 | a | a | a | a | a | a | a | a | a | a | a | a | a | a | a | a | a | a | a | a | a | a |
| RIL-150 | b | b | b | b | b | b | b | b | b | b | b | b | b | b | b | b | b | b | b | b | b | b |
| RIL-151 | a | a | a | a | a | a | b | - | b | b | b | b | b | b | b | - | b | b | b | b | a | b |
| RIL-152 | a | a | a | a | a | a | a | a | a | a | a | a | - | a | a | a | a | a | a | a | a | a |
| RIL-153 | b | b | b | b | b | b | b | b | b | b | b | b | b | b | b | b | b | b | b | b | b | b |
| RIL-154 | b | b | - | h | - | a | a | h | h | a | h | a | h | a | a | - | a | a | a | a | a | a |
| RIL-155 | b | b | b | b | b | b | b | b | b | b | b | b | b | b | b | b | b | b | b | b | b | b |
| RIL-156 | b | b | b | b | b | b | b | b | b | b | b | a | a | a | a | a | a | a | a | a | a | a |
| RIL-157 | b | b | b | b | b | b | b | b | b | b | b | a | a | a | a | a | a | a | a | a | a | a |
| RIL-158 | b | b | b | b | b | b | b | b | b | b | b | b | b | b | b | b | b | b | b | b | b | b |
| RIL-159 | b | b | b | b | b | b | b | b | b | b | b | b | a | b | b | b | b | b | b | b | b | b |
| RIL-160 | a | a | a | a | a | a | a | a | a | a | a | b | a | b | b | b | b | b | b | b | b | b |
| RIL-161 | b | b | b | b | b | b | b | b | b | b | b | a | a | a | a | a | b | b | b | b | b | b |
| RIL-162 | a | a | a | a | a | a | a | a | a | a | a | a | a | - | a | a | a | a | a | a | a | a |
| RIL-163 | a | a | a | a | a | a | a | a | a | a | a | b | b | b | b | b | b | b | b | b | b | b |
| RIL-164 | a | a | a | a | a | a | a | a | a | a | a | a | b | a | a | a | a | a | a | a | a | a |
| RIL-165 | b | b | b | b | b | b | b | b | b | b | b | a | a | a | a | a | a | a | a | a | a | a |
| RIL-166 | a | a | a | a | a | a | a | a | a | a | a | a | a | a | a | a | a | a | a | a | a | a |
| RIL-167 | a | a | a | a | a | a | a | a | a | a | a | a | a | a | a | a | a | a | a | a | a | a |
| RIL-168 | b | b | b | b | b | b | b | b | b | b | - | b | b | b | b | b | b | b | h | b | b | b |
| RIL-169 | a | a | a | a | a | a | a | a | a | a | a | b | b | b | b | b | b | b | b | b | b | b |
| RIL-170 | a | h | h | a | a | a | - | a | a | a | a | a | a | a | a | a | a | a | a | a | a | - |
| RIL-171 | b | b | b | b | b | b | b | b | b | b | b | b | b | b | b | b | b | b | b | b | h | b |
| RIL-172 | a | a | a | a | a | a | a | a | a | a | a | a | a | a | a | a | a | a | a | a | a | a |
| RIL-173 | a | a | a | a | a | a | a | a | a | a | a | a | a | a | a | a | a | a | a | a | a | a |
| RIL-174 | b | b | b | b | b | b | b | b | b | - | b | a | a | a | a | a | a | a | a | a | a | a |
| RIL-175 | a | a | a | a | a | a | a | a | a | - | b | b | b | b | b | b | b | b | b | b | a | b |
| RIL-176 | a | a | a | a | a | a | a | a | a | a | a | a | a | a | a | a | a | a | a | a | a | a |
| RIL-177 | a | a | a | a | a | a | a | a | a | - | a | b | b | b | b | b | b | b | b | b | b | b |
| RIL-178 | a | a | a | a | a | a | a | a | a | a | a | a | a | a | a | a | a | a | a | a | a | a |
| RIL-179 | a | a | a | a | a | a | a | a | a | a | a | b | b | b | b | b | b | b | b | b | b | b |
| RIL-180 | b | b | b | b | - | b | b | b | b | - | b | a | a | a | a | a | a | a | a | a | a | a |
| RIL-181 | a | b | h | a | a | a | b | a | a | a | a | a | a | a | a | a | a | a | a | a | a | a |
| RIL-182 | b | b | b | b | b | b | b | b | b | b | b | b | b | b | b | b | b | b | b | b | b | b |
| RIL-183 | a | a | a | - | a | a | a | a | a | - | a | a | a | a | a | a | a | a | a | a | a | a |
| RIL-184 | b | b | b | b | a | a | - | b | a | b | b | b | h | b | b | b | a | a | a | - | a | a |
| RIL-185 | b | b | b | b | b | b | b | b | b | - | b | a | a | a | a | a | a | a | a | a | a | a |
| RIL-186 | b | b | b | b | b | b | b | - | b | b | b | a | b | b | - | b | b | b | b | b | b | b |
| RIL-187 | a | b | - | a | - | - | - | - | a | - | b | b | b | b | b | - | b | - | - | - | a | - |
| RIL-188 | a | a | a | a | a | a | a | h | a | b | b | b | b | b | b | b | b | b | b | b | b | b |
| RIL-189 | a | a | a | a | a | a | a | a | a | a | a | b | - | b | b | - | - | b | b | b | b | b |
| RIL-190 | b | b | b | b | b | b | b | b | b | b | b | a | a | a | a | a | a | a | a | a | a | a |
| RIL-191 | a | a | a | a | a | a | a | a | a | a | a | a | a | a | a | a | a | a | a | a | a | a |
| RIL-192 | a | a | a | a | a | a | a | a | a | a | a | b | - | b | b | b | b | b | b | b | b | b |
| RIL-193 | b | b | b | b | b | b | b | b | b | b | b | a | a | a | a | a | a | a | a | a | a | a |
| RIL-194 | b | b | b | b | b | b | b | b | b | b | b | a | a | a | a | a | a | a | a | a | a | a |
| RIL-195 | b | b | b | b | b | b | b | b | b | b | b | b | b | b | b | b | b | b | b | b | b | b |
| RIL-196 | b | b | b | b | b | b | b | b | b | a | a | b | b | b | b | b | b | b | b | b | b | b |
| RIL-197 | b | b | b | b | b | b | b | b | b | b | b | b | b | b | b | b | b | b | b | b | b | b |
| RIL-198 | a | a | a | a | a | a | a | a | a | b | b | b | b | b | b | b | b | b | b | b | b | b |
| RIL-199 | a | a | a | a | a | a | a | a | a | a | a | a | a | a | a | a | a | a | a | a | a | a |
| RIL-200 | b | b | b | b | b | b | b | b | b | b | b | b | b | b | b | b | b | b | b | b | b | b |
| RIL-201 | - | a | a | a | a | a | a | a | a | a | a | b | b | b | b | b | b | b | b | b | b | b |
| RIL-202 | b | b | b | b | b | b | b | b | b | b | b | b | b | b | b | b | b | b | b | b | b | b |
| P27174 | a | a | a | a | a | a | a | a | a | a | a | a | a | a | a | a | a | a | a | a | a | a |
| Kiev Mutant | b | b | b | b | b | b | b | b | b | b | b | b | b | b | b | b | b | b | b | b | b | b |

# Supplementary Table S9. List of updated markers with percentages of RILs scored by GBS and PCR-based methods, Chi-square P-values for segregation distortion, previous and updated linkage group positions and LOD scores to adjacent loci on the updated map.

| **Name** | **RIL GBS data (%)** | **RIL PCR data (%)** | **Chi square**  **P value** | **Linkage group** | **Old locus cM** | **New locus**  **cM (±SD)** | **LOD**  **scores** |
| --- | --- | --- | --- | --- | --- | --- | --- |
| TP23903 | 41.3 | 99.0 | 0.36 | ALB02 | 0.00 | 0.00 (±0.00) | 45.3 |
| TP229924 | 68.9 | 98.0 | 0.33 | ALB02 | 0.00 | 2.14 (±0.04) | 45.3, 54.2 |
| WANR1^a^ | 47.4 | 99.0 | 0.27 | ALB02 | 0.38 | 2.48 (±0.26) | 54.2, 55.1 |
| TP222136 | 89.3 | 99.0 | 0.36 | ALB02 | 0.38 | 2.83 (±0.03) | 55.1, 30.1 |
| TP272531 | 60.2 | 98.0 | 0.31 | ALB02 | 0.38 | 3.17 (±0.04) | 30.1, 54.8 |
| TP47110 | 70.9 | 99.0 | 0.53 | ALB02 | 1.26 | 3.52 (±0.05) | 54.8, 23.5 |
| TP291372 | 77.6 | 98.0 | 0.39 | ALB02 | 2.64 | 5.89 (±0.51) | 51.5, 49.9 |
| TP446132 | 57.7 | 98.0 | 0.61 | ALB02 | 2.64 | 6.69 (±0.59) | 49.9, 54.5 |
| TP237794 | 89.8 | 99.5 | 0.68 | ALB02 | 2.64 | 6.95 (±0.51) | 54.5, 40.3 |
| TP88533 | 90.8 | 99.0 | 0.31 | ALB02 | 4.74 | 9.44 (±0.51) | 54.2, 25.3 |
| TP38227 | 91.3 | 95.9 | 0.30 | ALB02 | 4.74 | 9.70 (±0.51) | 24.7, 22.3 |
| TP149038 | 74.5 | 96.9 | 0.64 | ALB04 | 115.99 | 93.31 (±1.69) | 49.7, 48.5 |
| TP416765 | 64.3 | 99.0 | 0.67 | ALB04 | 117.41 | 96.70 (±2.18) | 39.4, 19.3 |
| WANR3 | 47.4 | 99.5 | 0.68 | ALB04 | 117.41 | 100.00 (±2.13) | 18.6, 45.5 |
| TP3712 | 92.9 | 98.5 | 0.65 | ALB04 | 125.39 | 102.37 (±2.16) | 32.1, 56.0 |
| TP364001 | 92.9 | 95.9 | 0.67 | ALB04 | 125.39 | 102.37 (±2.16) | 56.0, 29.2 |
| TP37593 | 55.1 | 99.0 | 0.66 | ALB04 | 127.30 | 104.52 (±0.62) | 53.3, 51.4 |
| TP26007 | 90.8 | 99.0 | 0.66 | ALB04 | 127.30 | 104.52 (±0.62) | 42.9, 53.3 |
| TP106254 | 83.2 | 99.0 | 0.92 | ALB04 | 127.94 | 105.36 (±0.74) | 51.4, 51.1 |
| TP93026 | 59.7 | 98.5 | 0.60 | ALB04 | 128.41 | 106.20 (±1.73) | 51.1, 41.2 |
| TP338761 | 80.6 | 98.5 | 0.54 | ALB04 | 128.41 | 107.89 (±1.61) | 44.7, 26.8 |
| TP440375 | 76.5 | 99.5 | 0.95 | ALB04 | 128.41 | 109.30 (±0.0) | 27.1 |

^a^ as previously published by Yang et al. 2010)

# Supplementary Table S10. Updated white lupin linkage groups ALB02 and ALB04.

Markers with updated segregation were named with “a” suffix.

| **Linkage group** | **Marker** | **Position cM** | **Linkage group** | **Marker** | **Position cM** |
| --- | --- | --- | --- | --- | --- |
| ALB02 | TP23903a | 0.00 | ALB04 | TP85114 | 0.000 |
| ALB02 | TP229924a | 2.14 | ALB04 | TP412583 | 0.000 |
| ALB02 | WANR1a | 2.48 | ALB04 | TP429796 | 0.000 |
| ALB02 | TP222136a | 2.83 | ALB04 | TP168090 | 0.000 |
| ALB02 | TP24947 | 3.00 | ALB04 | TP291286 | 0.794 |
| ALB02 | TP272531a | 3.17 | ALB04 | TP408444 | 2.408 |
| ALB02 | TP47110a | 3.52 | ALB04 | TP236093 | 2.408 |
| ALB02 | TP458 | 3.79 | ALB04 | TP264517 | 2.408 |
| ALB02 | TP135791 | 4.11 | ALB04 | TP227947 | 2.408 |
| ALB02 | TP94529 | 4.11 | ALB04 | TP10304 | 2.408 |
| ALB02 | TP254603 | 4.30 | ALB04 | TP447720 | 2.408 |
| ALB02 | TP2471 | 4.70 | ALB04 | TP99002 | 2.408 |
| ALB02 | TP236266 | 5.60 | ALB04 | TP1016 | 3.289 |
| ALB02 | TP323017 | 5.60 | ALB04 | TP320828 | 4.169 |
| ALB02 | TP13313 | 5.60 | ALB04 | TP270309 | 4.169 |
| ALB02 | TP231214 | 5.60 | ALB04 | TP95472 | 4.783 |
| ALB02 | TP291372a | 5.89 | ALB04 | TP271747 | 5.712 |
| ALB02 | TP446132a | 6.69 | ALB04 | TP34445 | 5.712 |
| ALB02 | TP237794a | 6.95 | ALB04 | TP430213 | 5.712 |
| ALB02 | TP68244 | 9.44 | ALB04 | TP390059 | 6.119 |
| ALB02 | TP88533a | 9.44 | ALB04 | TP111425 | 6.119 |
| ALB02 | TP152838 | 9.63 | ALB04 | TP95619 | 6.119 |
| ALB02 | TP38227a | 9.70 | ALB04 | TP54880 | 6.939 |
| ALB02 | TP258530 | 11.38 | ALB04 | TP412857 | 6.939 |
| ALB02 | M65E35C315 | 11.47 | ALB04 | TP284785 | 6.939 |
| ALB02 | TP8164 | 11.49 | ALB04 | TP402052 | 6.939 |
| ALB02 | TP262598 | 11.97 | ALB04 | TP90179 | 6.939 |
| ALB02 | TP22620 | 12.31 | ALB04 | M66E32A114 | 7.522 |
| ALB02 | TP330531 | 17.41 | ALB04 | lup93 | 7.522 |
| ALB02 | TP418891 | 17.42 | ALB04 | TP333120 | 9.652 |
| ALB02 | TP156253 | 18.79 | ALB04 | TP116439 | 9.652 |
| ALB02 | TP52677 | 19.93 | ALB04 | TP333121 | 9.652 |
| ALB02 | TP76043 | 19.93 | ALB04 | TP27876 | 9.934 |
| ALB02 | TP271235 | 20.63 | ALB04 | TP288609 | 9.934 |
| ALB02 | TP259052 | 20.63 | ALB04 | TP45824 | 10.686 |
| ALB02 | TP12213 | 20.63 | ALB04 | TP28589 | 10.686 |
| ALB02 | Lup313 | 20.74 | ALB04 | TP409424 | 10.686 |
| ALB02 | TP27066 | 20.93 | ALB04 | TP294609 | 11.220 |
| ALB02 | TP284610 | 20.93 | ALB04 | TP296318 | 12.369 |
| ALB02 | TP113073 | 21.32 | ALB04 | TP236034 | 14.218 |
| ALB02 | TP1839 | 22.49 | ALB04 | TP152770 | 14.943 |
| ALB02 | TP238590 | 23.75 | ALB04 | TP294650 | 14.943 |
| ALB02 | TP325389 | 24.48 | ALB04 | TP213815 | 14.943 |
| ALB02 | TP47049 | 24.48 | ALB04 | TP11961 | 14.943 |
| ALB02 | TP235826 | 24.48 | ALB04 | TP6207 | 14.943 |
| ALB02 | TP73282 | 24.90 | ALB04 | TP7395 | 16.622 |
| ALB02 | TP274906 | 24.90 | ALB04 | TP26280 | 17.001 |
| ALB02 | TP390179 | 24.90 | ALB04 | TP283602 | 18.566 |
| ALB02 | TP280948 | 24.90 | ALB04 | TP433066 | 18.566 |
| ALB02 | TP29252 | 24.90 | ALB04 | TP451694 | 18.566 |
| ALB02 | TP413110 | 25.59 | ALB04 | TP49051 | 21.380 |
| ALB02 | TP435069 | 25.59 | ALB04 | TP226437 | 22.271 |
| ALB02 | TP23060 | 25.59 | ALB04 | TP215559 | 22.271 |
| ALB02 | TP281869 | 27.37 | ALB04 | TP432760 | 22.271 |
| ALB02 | TP263332 | 27.37 | ALB04 | TP51864 | 22.271 |
| ALB02 | DSI | 27.98 | ALB04 | TP83912 | 23.090 |
| ALB02 | TP238723 | 27.98 | ALB04 | TP281829 | 23.090 |
| ALB02 | TP305240 | 27.98 | ALB04 | TP366148 | 23.090 |
| ALB02 | TP275341 | 28.04 | ALB04 | TP147627 | 23.413 |
| ALB02 | TP74427 | 28.94 | ALB04 | TP36219 | 24.742 |
| ALB02 | LSSR1b | 28.94 | ALB04 | TP297189 | 24.742 |
| ALB02 | TP78167 | 28.94 | ALB04 | TP7256 | 25.011 |
| ALB02 | TP5398 | 28.94 | ALB04 | TP450898 | 25.011 |
| ALB02 | TP213551 | 29.59 | ALB04 | TP233363 | 25.011 |
| ALB02 | LSSR1a | 29.59 | ALB04 | TP223390 | 25.011 |
| ALB02 | TP287384 | 30.23 | ALB04 | TP85618 | 25.011 |
| ALB02 | TP261526 | 30.23 | ALB04 | TP13116 | 25.555 |
| ALB02 | TP38832 | 30.23 | ALB04 | TP26850 | 25.555 |
| ALB02 | TP224508 | 33.48 | ALB04 | TP295274 | 25.555 |
| ALB02 | TP376433 | 34.24 | ALB04 | TP280013 | 27.769 |
| ALB02 | TP236229 | 34.26 | ALB04 | LSSR16-2 | 28.444 |
| ALB02 | TP73833 | 34.26 | ALB04 | TP10483 | 28.444 |
| ALB02 | TP233016 | 34.27 | ALB04 | TP98592 | 31.079 |
| ALB02 | CALTL | 35.76 | ALB04 | TP406223 | 31.395 |
| ALB02 | TP59439 | 35.76 | ALB04 | TP353134 | 32.902 |
| ALB02 | TP227862 | 36.29 | ALB04 | TP380722 | 33.638 |
| ALB02 | M48E32A188 | 36.29 | ALB04 | TP330881 | 33.638 |
| ALB02 | TP147007 | 36.29 | ALB04 | TP237496 | 33.948 |
| ALB02 | TP338512 | 36.29 | ALB04 | TP198678 | 33.948 |
| ALB02 | TP70881 | 36.60 | ALB04 | TP282223 | 33.948 |
| ALB02 | TP266604 | 36.60 | ALB04 | TP91394 | 34.370 |
| ALB02 | TP414637 | 37.86 | ALB04 | TP450471 | 34.792 |
| ALB02 | TP2927 | 39.13 | ALB04 | TP274186 | 35.071 |
| ALB02 | TP376589 | 39.13 | ALB04 | TP20691 | 35.071 |
| ALB02 | TP340895 | 39.13 | ALB04 | TP29570 | 35.071 |
| ALB02 | TP275722 | 40.20 | ALB04 | TP262648 | 35.071 |
| ALB02 | TP270275 | 40.20 | ALB04 | TP76619 | 35.923 |
| ALB02 | TP15121 | 41.44 | ALB04 | TP447185 | 35.923 |
| ALB02 | lPms-522533 | 41.77 | ALB04 | TP226000 | 36.204 |
| ALB02 | lPms-533193 | 41.77 | ALB04 | TP104710 | 38.213 |
| ALB02 | lPms-524434 | 41.78 | ALB04 | TP271784 | 38.213 |
| ALB02 | lPms-527037 | 41.79 | ALB04 | TP21756 | 40.232 |
| ALB02 | TP215446 | 41.82 | ALB04 | M76E35A194 | 40.558 |
| ALB02 | TP23177 | 42.62 | ALB04 | TP451781 | 41.514 |
| ALB02 | TP242382 | 43.25 | ALB04 | TP429301 | 43.607 |
| ALB02 | TP73593 | 43.72 | ALB04 | lPms-750651 | 44.142 |
| ALB02 | TP70123 | 44.04 | ALB04 | lPms-750885 | 44.142 |
| ALB02 | TP409840 | 44.04 | ALB04 | TP2722 | 44.997 |
| ALB02 | TP91431 | 44.04 | ALB04 | TP377994 | 44.997 |
| ALB02 | TP265407 | 45.87 | ALB04 | TP36760 | 45.369 |
| ALB02 | TP156581 | 45.87 | ALB04 | TP156702 | 45.370 |
| ALB02 | TP113243 | 47.65 | ALB04 | TP273308 | 45.370 |
| ALB02 | TP99366 | 50.49 | ALB04 | TP117990 | 45.691 |
| ALB02 | TP79211 | 50.50 | ALB04 | TP260170 | 46.929 |
| ALB02 | lPms-525247 | 53.79 | ALB04 | TP90046 | 47.330 |
| ALB02 | lPms-524499 | 53.79 | ALB04 | TP330657 | 47.332 |
| ALB02 | lPms-525389 | 53.79 | ALB04 | M75E32A283 | 48.002 |
| ALB02 | lPms-532980 | 53.79 | ALB04 | TP4085 | 48.551 |
| ALB02 | lPms-525206 | 53.79 | ALB04 | TP79477 | 48.553 |
| ALB02 | lPms-517115 | 53.79 | ALB04 | TP48165 | 48.553 |
| ALB02 | TP90243 | 54.28 | ALB04 | M65E32B279 | 48.927 |
| ALB02 | TP292384 | 54.33 | ALB04 | lPms-515808 | 51.020 |
| ALB02 | TP74140 | 54.33 | ALB04 | lPms-516192 | 51.020 |
| ALB02 | TP230400 | 54.33 | ALB04 | lPms-515888 | 51.020 |
| ALB02 | TP99192 | 56.72 | ALB04 | TP101279 | 54.461 |
| ALB02 | TP251175 | 57.49 | ALB04 | TP19090 | 57.639 |
| ALB02 | TP413765 | 57.49 | ALB04 | TP272393 | 60.096 |
| ALB02 | TP246733 | 57.49 | ALB04 | TP285116 | 60.096 |
| ALB02 | TP243375 | 57.49 | ALB04 | TP370389 | 60.096 |
| ALB02 | TP437988 | 59.82 | ALB04 | TP46065 | 60.650 |
| ALB02 | TP28359 | 61.08 | ALB04 | M77E38A165 | 60.650 |
| ALB02 | TP225469 | 62.27 | ALB04 | TP362226 | 60.650 |
| ALB02 | TP94870 | 62.65 | ALB04 | TP87632 | 61.534 |
| ALB02 | TP28057 | 62.65 | ALB04 | TP96144 | 61.534 |
| ALB02 | TP154184 | 62.65 | ALB04 | TP1017 | 63.994 |
| ALB02 | TP323343 | 64.08 | ALB04 | TP4959 | 63.994 |
| ALB02 | TP24959 | 64.08 | ALB04 | TP140317 | 63.994 |
| ALB02 | TP224596 | 67.92 | ALB04 | TP97207 | 63.994 |
| ALB02 | TP128324 | 67.92 | ALB04 | TP340521 | 65.659 |
| ALB02 | TP151960 | 67.92 | ALB04 | TP156909 | 65.659 |
| ALB02 | TP248931 | 68.50 | ALB04 | M78E38A116 | 66.304 |
| ALB02 | TP258773 | 69.09 | ALB04 | TP76928 | 66.745 |
| ALB02 | TP78536 | 69.09 | ALB04 | TP3626 | 66.745 |
| ALB02 | TP231446 | 69.88 | ALB04 | TP174329 | 66.745 |
| ALB02 | TP319284 | 70.27 | ALB04 | TP21317 | 66.745 |
| ALB02 | TP7383 | 70.27 | ALB04 | TP240160 | 69.414 |
| ALB02 | TP109912 | 70.27 | ALB04 | TP31574 | 71.766 |
| ALB02 | lPms-517297 | 72.56 | ALB04 | TP5833 | 72.352 |
| ALB02 | TP5188 | 73.27 | ALB04 | M63E38A304 | 72.352 |
| ALB02 | TP226595 | 73.27 | ALB04 | TP156518 | 74.002 |
| ALB02 | TP63661 | 73.27 | ALB04 | TP6216 | 74.002 |
| ALB02 | TP325533 | 73.27 | ALB04 | TP224974 | 74.645 |
| ALB02 | TP437050 | 73.27 | ALB04 | TP295286 | 78.138 |
| ALB02 | M76E32A128 | 74.35 | ALB04 | TP257123 | 78.139 |
| ALB02 | M63E38A204 | 75.86 | ALB04 | TP47426 | 79.063 |
| ALB02 | M75E38B244 | 76.16 | ALB04 | WANR2 | 79.063 |
| ALB02 | M65E32A100 | 76.45 | ALB04 | TP309516 | 79.063 |
| ALB02 | TP232432 | 77.12 | ALB04 | TP100752 | 81.266 |
| ALB02 | TP33442 | 77.12 | ALB04 | TP81412 | 81.266 |
| ALB02 | TP431257 | 77.12 | ALB04 | TP449451 | 83.469 |
| ALB02 | TP299131 | 78.33 | ALB04 | TP410385 | 83.469 |
| ALB02 | TP349407 | 78.33 | ALB04 | TP116058 | 83.469 |
| ALB02 | TP233650 | 78.33 | ALB04 | TP260558 | 83.979 |
| ALB02 | TP340809 | 78.33 | ALB04 | TP71029 | 84.027 |
| ALB02 | M63E38A202 | 79.11 | ALB04 | TP22756 | 85.262 |
| ALB02 | lPms-515855 | 79.45 | ALB04 | M66E32B386 | 85.262 |
| ALB02 | M64E41A148 | 79.45 | ALB04 | TP400902 | 85.573 |
| ALB02 | TP23446 | 80.13 | ALB04 | TP9417 | 85.606 |
| ALB02 | TP19741 | 80.14 | ALB04 | TP27938 | 86.399 |
| ALB02 | TP429721 | 80.99 | ALB04 | TP26890 | 87.194 |
| ALB02 | TP262063 | 80.99 | ALB04 | TP445144 | 87.713 |
| ALB02 | TP101421 | 82.91 | ALB04 | TP155926 | 87.713 |
| ALB02 | TP445667 | 83.49 | ALB04 | TP102131 | 89.008 |
| ALB02 | TP19988 | 83.61 | ALB04 | TP152487 | 89.008 |
| ALB02 | TP288585 | 83.63 | ALB04 | TP232900 | 89.008 |
| ALB02 | TP114572 | 83.63 | ALB04 | TP390425 | 90.401 |
| ALB02 | TP39614 | 83.63 | ALB04 | M59E32B265 | 90.401 |
| ALB02 | lPms-515411 | 83.63 | ALB04 | TP210950 | 91.143 |
| ALB02 | lPms-515626 | 84.00 | ALB04 | TP333642 | 91.143 |
| ALB02 | lPms-525122 | 84.00 | ALB04 | TP276324 | 91.143 |
| ALB02 | TP238828 | 84.57 | ALB04 | TP11994 | 92.945 |
| ALB02 | TP292201 | 85.38 | ALB04 | TP149038a | 93.309 |
| ALB02 | TP100999 | 87.27 | ALB04 | TP89744 | 93.309 |
| ALB02 | TP25763 | 87.93 | ALB04 | TP154225 | 93.309 |
| ALB02 | TP439845 | 88.28 | ALB04 | TP270602 | 93.309 |
| ALB02 | TP95056 | 89.35 | ALB04 | TP272081 | 96.704 |
| ALB02 | TP256124 | 89.35 | ALB04 | TP416765a | 96.704 |
| ALB02 | TP12311 | 89.63 | ALB04 | LG96 | 98.196 |
| ALB02 | TP241628 | 90.77 | ALB04 | WANR3a | 99.995 |
| ALB02 | M48E32B277 | 91.53 | ALB04 | TP73498 | 99.995 |
| ALB02 | M61E32B396 | 93.45 | ALB04 | TP432453 | 100.506 |
| ALB02 | TP58457 | 94.85 | ALB04 | TP21440 | 101.052 |
| ALB02 | TP56963 | 95.17 | ALB04 | TP445741 | 101.052 |
| ALB02 | TP15653 | 95.50 | ALB04 | TP96614 | 101.052 |
| ALB02 | TP235608 | 95.50 | ALB04 | TP61122 | 101.378 |
| ALB02 | TP264653 | 95.83 | ALB04 | TP345661 | 101.873 |
| ALB02 | TP408623 | 96.75 | ALB04 | TP3712a | 102.368 |
| ALB02 | TP278885 | 98.44 | ALB04 | TP364001a | 102.368 |
| ALB02 | TP94353 | 98.44 | ALB04 | TP444157 | 102.368 |
| ALB02 | TP115697 | 98.77 | ALB04 | TP277222 | 103.914 |
| ALB02 | TP237672 | 101.64 | ALB04 | TP327093 | 103.914 |
| ALB02 | TP285942 | 101.64 | ALB04 | TP26007a | 104.517 |
| ALB02 | TP450480 | 101.64 | ALB04 | TP338317 | 104.517 |
| ALB02 | TP250121 | 101.65 | ALB04 | TP37593a | 104.517 |
| ALB02 | TP119917 | 102.32 | ALB04 | TP106254a | 105.358 |
| ALB02 | TP94867 | 105.93 | ALB04 | TP93026a | 106.198 |
| ALB02 | TP114357 | 106.90 | ALB04 | TP9255 | 107.331 |
| ALB02 | TP100814 | 107.99 | ALB04 | TP338761a | 107.889 |
| ALB02 | TP54890 | 107.99 | ALB04 | TP287341 | 108.605 |
| ALB02 | TP27589 | 107.99 | ALB04 | TP440375a | 109.303 |
| ALB02 | TP277893 | 109.75 |  |  |  |
| ALB02 | TP283681 | 109.75 |  |  |  |
| ALB02 | TP152958 | 109.75 |  |  |  |
| ALB02 | TP92269 | 111.73 |  |  |  |
| ALB02 | TP130298 | 111.73 |  |  |  |
| ALB02 | TP260154 | 111.73 |  |  |  |
| ALB02 | TP405457 | 113.64 |  |  |  |
| ALB02 | TP447033 | 113.97 |  |  |  |
| ALB02 | TP447301 | 114.81 |  |  |  |
| ALB02 | M48E35A161 | 115.66 |  |  |  |
| ALB02 | TP271658 | 116.83 |  |  |  |
| ALB02 | TP20987 | 116.83 |  |  |  |
| ALB02 | TP327232 | 116.83 |  |  |  |
| ALB02 | TP234977 | 119.42 |  |  |  |
| ALB02 | TP261886 | 119.42 |  |  |  |

# Supplementary Table S11. Marker scores for white lupin lines from world germplasm collection.

Line names in the Supplementary Table 4

D, line P27174; K, Kiev Mutant

| **Field No.** | **TP23903** | **WANR1** | **TP229924** | **TP272531** | **TP222136** | **TP47110** | **TP446132** | **TP291372** | **TP237794** | **TP38227** | **TP88533** | **TP149038** | **TP416765** | **WANR3** | **TP3712** | **TP364001** | **TP37593** | **TP26007** | **TP106254** | **TP338761** | **TP440375** | **TP93026** |
| --- | --- | --- | --- | --- | --- | --- | --- | --- | --- | --- | --- | --- | --- | --- | --- | --- | --- | --- | --- | --- | --- | --- |
| **1** | B | B | B | B | B | B | A | A | A | B | B | A | A | A | A | A | B | B | B | B | A | A |
| **3** | A | B | B | B | B | B | A | B | A | B | B | B | A | B | B | B | A | B | A | B | B | B |
| **4** | B | A | B | B | B | B | H | A | A | B | B | B | A | B | B | H | A | B | A | B | B | A |
| **6** | B | A | A | B | B | B | A | B | A | B | B | B | B | B | B | B | B | B | B | B | A | A |
| **7** | B | B | B | B | B | B | H | B | B | B | B | B | A | A | B | B | B | A | B | B | A | A |
| **9** | A | B | B | B | B | B | A | A | A | A | A | B | H | H | B | H | B | B | A | C | H | H |
| **10** | B | A | B | A | B | B | H | A | A | H | H | B | B | A | B | H | B | B | B | A | B | B |
| **11** | A | B | B | B | B | B | A | A | A | A | A | B | A | B | B | H | A | B | A | B | B | B |
| **12** | A | B | B | B | B | B | A | A | A | A | A | A | A | H | B | H | B | A | B | B | A | A |
| **13** | A | B | B | B | B | A | A | A | A | B | B | B | A | A | B | H | B | B | A | B | A | A |
| **14** | A | B | B | A | B | B | B | A | A | B | B | A | A | H | A | H | B | B | B | C | A | A |
| **15** | A | B | B | B | B | B | A | A | A | H | B | B | B | B | A | A | B | B | B | B | A | A |
| **17** | A | B | B | A | B | B | A | A | A | A | A | B | B | B | B | B | B | A | B | A | A | A |
| **18** | B | B | B | B | B | B | B | B | B | B | B | B | B | B | B | B | B | B | B | B | B | B |
| **19** | A | A | A | B | B | B | A | A | A | A | A | A | B | A | A | H | A | B | A | B | H | H |
| **20** | A | B | B | B | B | B | A | A | A | H | A | A | A | B | B | H | H | A | B | B | A | A |
| **21** | B | B | B | B | B | B | B | B | B | B | B | B | A | A | A | H | A | A | B | B | B | B |
| **22** | B | B | B | A | B | A | A | A | A | A | A | B | B | B | B | H | B | B | B | B | A | A |
| **23** | B | B | B | B | B | B | A | A | A | B | B | B | B | B | B | B | B | B | B | B | B | B |
| **24** | A | B | A | B | B | B | A | A | A | H | A | B | B | B | B | H | B | B | B | B | B | B |
| **25** | A | A | A | A | B | B | A | A | A | A | A | A | B | B | A | A | B | A | A | B | A | A |
| **26** | A | B | A | A | B | B | A | B | A | H | A | B | A | B | B | B | A | B | A | B | B | B |
| **27** | A | B | B | A | B | B | A | B | A | A | A | A | B | B | A | A | A | B | B | A | B | B |
| **28** | B | B | B | B | B | B | B | B | B | B | B | B | B | B | B | B | B | B | B | B | B | B |
| **29** | B | B | B | B | A | B | B | B | B | B | B | A | B | B | B | B | B | A | B | B | A | A |
| **30** | B | A | B | B | B | B | A | B | A | A | A | A | A | B | A | H | A | B | A | B | B | B |
| **31** | A | B | B | B | B | B | A | A | A | A | A | B | B | B | B | B | B | B | B | B | B | B |
| **32** | A | A | A | B | B | B | A | A | A | H | H | B | B | A | A | A | B | A | A | B | B | B |
| **33** | B | A | A | B | B | B | A | A | A | H | H | H | A | B | B | H | A | B | A | B | H | H |
| **34** | A | A | A | B | B | B | A | A | A | H | A | A | B | B | A | A | A | B | B | B | A | A |
| **35** | A | B | A | A | B | B | A | B | A | A | A | A | A | B | A | A | B | A | B | B | A | A |
| **36** | A | B | A | B | B | B | B | A | B | A | A | A | A | B | B | B | A | A | B | B | B | B |
| **37** | B | B | B | B | A | B | H | A | A | H | B | A | A | A | B | A | A | B | A | B | B | B |
| **38** | B | B | B | B | B | B | B | A | B | B | B | B | B | B | B | B | B | B | B | B | B | B |
| **39** | B | A | B | B | B | B | H | A | A | H | H | B | A | C | B | H | B | B | B | B | B | B |
| **40** | B | B | B | B | B | B | A | A | A | B | B | A | B | B | B | B | B | B | B | B | B | B |
| **43** | B | B | B | B | B | B | B | B | B | B | B | B | B | B | B | B | B | B | B | A | B | B |
| **44** | B | B | B | B | B | B | H | A | A | B | B | B | B | B | B | B | B | C | A | B | B | B |
| **45** | B | A | A | B | B | B | B | A | B | A | A | B | B | B | B | H | B | A | B | A | B | B |
| **46** | A | B | B | A | B | B | A | A | A | B | B | A | A | B | A | H | A | B | B | B | A | B |
| **47** | B | B | B | B | B | B | A | A | A | B | B | B | B | B | B | B | B | B | B | C | B | B |
| **48** | B | A | B | A | B | B | A | A | B | B | B | B | B | B | B | B | B | B | B | B | A | A |
| **49** | B | B | B | B | B | B | B | B | A | B | B | A | B | B | B | A | A | B | B | B | A | A |
| **50** | B | B | B | B | B | B | B | B | B | B | B | A | A | B | A | H | A | B | B | A | B | B |
| **53** | B | B | B | B | B | B | B | B | B | B | B | B | B | B | B | B | B | B | B | B | B | B |
| **58** | A | A | B | B | B | B | A | B | A | A | A | A | A | B | B | B | A | C | B | B | B | B |
| **59** | A | A | B | B | B | B | A | B | A | A | A | A | B | B | A | A | A | A | B | B | A | A |
| **60** | B | B | B | B | B | B | A | B | A | A | A | A | A | B | B | A | B | A | A | B | A | A |
| **61** | B | A | B | A | B | B | A | A | A | B | B | A | B | C | A | A | B | A | B | B | A | A |
| **62** | A | B | B | B | B | B | A | B | A | A | A | B | B | B | A | A | B | B | B | B | B | B |
| **63** | B | B | B | B | B | B | A | A | A | B | B | A | A | A | B | A | A | B | B | A | B | B |
| **64** | A | A | B | A | B | B | B | B | B | B | B | A | A | A | B | A | A | B | B | B | B | B |
| **65** | B | B | B | B | B | B | A | A | A | A | A | H | B | B | B | B | A | A | B | B | A | A |
| **66** | B | B | B | B | B | B | B | B | B | B | B | B | B | B | A | H | A | A | B | B | B | B |
| **68** | A | B | B | A | B | B | A | B | A | A | A | B | B | A | A | A | B | A | B | A | A | A |
| **69** | A | A | B | B | B | B | A | A | A | B | B | A | A | B | A | A | A | A | A | B | B | A |
| **70** | A | B | A | B | B | B | B | B | B | B | B | A | A | B | B | H | B | A | B | B | A | A |
| **71** | A | B | A | B | B | B | H | A | A | A | A | B | B | A | A | H | B | B | A | B | B | B |
| **72** | B | B | B | B | B | B | A | A | A | B | B | B | B | B | B | B | B | B | B | B | B | B |
| **74** | A | A | A | B | B | B | B | A | A | A | A | B | B | B | B | H | B | B | B | B | B | B |
| **75** | B | B | B | B | B | B | A | A | A | B | B | A | B | B | B | B | B | B | B | A | B | B |
| **76** | B | B | B | B | B | B | A | A | A | B | B | B | B | B | B | B | B | B | B | B | B | B |
| **77** | B | B | B | B | B | B | A | A | A | A | A | B | A | A | A | A | B | B | A | B | H | H |
| **78** | A | B | B | B | B | B | A | A | A | A | A | B | B | B | B | B | B | A | B | B | A | A |
| **79** | B | A | A | B | B | B | A | A | A | A | A | B | A | A | A | H | A | B | A | B | A | A |
| **80** | B | B | B | B | B | B | A | A | A | A | A | A | B | B | B | A | B | B | B | B | B | B |
| **81** | B | B | B | B | B | B | A | A | A | H | B | A | A | A | A | A | B | B | A | B | B | B |
| **82** | B | B | B | B | B | B | A | A | A | B | B | A | A | B | B | H | B | A | B | B | B | B |
| **83** | B | B | B | B | B | B | A | A | A | B | B | B | A | A | A | H | H | A | A | B | A | A |
| **84** | B | B | B | B | B | B | B | B | B | B | B | B | B | A | A | H | B | A | A | B | B | B |
| **85** | B | B | B | B | B | B | B | B | B | B | B | B | B | B | B | B | B | B | B | B | B | B |
| **86** | B | A | A | B | B | A | A | A | A | B | B | A | A | B | A | A | A | A | A | B | B | B |
| **87** | A | B | A | B | B | B | H | A | B | A | A | B | A | H | A | A | B | B | B | C | H | H |
| **88** | A | A | A | B | B | B | A | A | A | A | A | B | A | A | A | A | B | A | A | B | B | B |
| **89** | A | A | B | B | B | B | A | A | A | A | A | A | A | A | A | H | A | B | A | B | B | B |
| **90** | B | B | B | B | B | B | A | A | A | B | B | B | B | B | B | H | B | B | B | B | B | B |
| **91** | A | B | B | B | B | B | A | B | A | B | B | B | B | B | B | H | B | A | A | B | A | A |
| **92** | A | B | B | B | B | B | A | B | A | A | A | A | B | A | A | A | A | A | B | B | B | B |
| **93** | B | B | B | B | B | B | A | A | A | B | B | H | B | B | B | H | B | B | B | B | B | B |
| **94** | A | B | A | A | B | B | H | A | A | H | H | H | B | A | A | A | B | B | A | B | B | B |
| **95** | A | B | B | B | B | B | B | B | A | H | H | A | B | H | A | A | A | A | A | B | A | A |
| **96** | A | B | B | B | B | B | A | A | A | A | A | A | B | A | A | A | A | A | A | A | B | B |
| **97** | B | A | B | B | B | B | A | A | A | A | A | A | B | A | A | H | B | A | A | B | A | A |
| **98** | B | A | B | B | B | B | A | A | A | A | A | H | A | A | A | H | A | B | A | B | B | B |
| **99** | A | B | B | B | B | B | A | A | B | B | B | H | A | A | A | A | B | B | B | B | B | B |
| **100** | B | B | B | B | B | B | B | A | B | B | B | H | A | A | B | H | A | B | A | B | B | B |
| **101** | B | A | A | B | B | B | A | B | B | B | B | A | B | B | B | A | A | B | B | B | B | B |
| **102** | B | B | B | B | B | B | A | A | A | A | A | H | H | H | A | A | A | A | A | B | B | B |
| **103** | B | B | B | B | B | B | B | A | A | B | B | H | B | B | B | B | B | B | B | B | B | B |
| **104** | A | A | A | B | B | B | A | A | A | A | A | H | A | B | A | A | B | A | B | B | B | B |
| **105** | B | B | B | B | B | B | B | B | B | B | B | B | B | B | B | B | B | B | B | B | B | B |
| **106** | A | B | B | A | B | B | A | A | A | A | A | B | A | B | A | A | B | A | B | A | B | B |
| **107** | B | B | B | B | B | B | B | B | B | B | B | B | B | B | B | B | B | B | A | B | B | B |
| **108** | A | B | B | B | B | B | A | A | A | A | A | A | B | A | A | A | A | A | B | B | B | B |
| **109** | A | B | B | B | B | B | A | A | A | B | B | B | A | A | B | H | A | B | B | C | A | H |
| **110** | B | B | B | B | B | B | B | B | B | H | B | B | B | A | A | H | B | A | A | B | B | B |
| **111** | A | B | B | B | B | B | A | A | A | A | A | B | A | B | A | A | B | A | B | B | B | B |
| **112** | B | A | A | A | A | B | A | A | A | A | A | A | B | B | B | B | B | B | B | B | B | B |
| **113** | A | B | B | B | B | B | A | A | A | A | A | B | H | A | A | A | B | A | A | B | A | H |
| **114** | A | B | B | B | B | B | H | A | A | A | A | A | A | A | A | A | B | B | B | B | B | B |
| **115** | A | B | B | A | B | B | A | A | A | A | A | B | A | B | A | A | B | A | A | B | B | B |
| **116** | B | B | B | A | B | A | A | A | A | B | B | B | B | B | B | B | B | B | B | B | B | B |
| **117** | A | A | A | B | B | B | A | A | A | B | B | B | B | B | B | B | B | B | B | B | B | B |
| **132** | B | B | B | B | B | B | B | B | B | B | B | B | B | B | B | B | B | B | B | A | B | B |
| **133** | A | B | H | B | A | B | A | B | A | B | B | A | A | B | B | B | A | B | A | B | B | A |
| **K** | B | B | B | B | B | B | B | B | B | B | B | B | B | B | B | B | B | B | B | B | B | B |
| **D** | A | A | A | A | A | A | A | A | A | A | A | A | A | A | A | A | A | A | A | A | A | A |

# Supplementary Table S12. Genes identified in the regions of white lupin genome carrying anthracnose resistance loci.

| **Chr** | **Chr start** | **Chr end** | **Strand** | **Type** | **Locus** | **Product** |
| --- | --- | --- | --- | --- | --- | --- |
| Chr02 | 24162 | 25978 | + | mRNA | Lalb_Chr02g0140531 | Putative major facilitator, sugar transporter, major facilitator superfamily |
| Chr02 | 26434 | 30004 | - | mRNA | Lalb_Chr02g0140541 | Putative non-specific serine/threonine protein kinase |
| Chr02 | 32475 | 35441 | - | mRNA | Lalb_Chr02g0140551 | Putative transferase |
| Chr02 | 40728 | 41033 | - | mRNA | Lalb_Chr02g0140561 | Putative pentatricopeptide |
| Chr02 | 41064 | 41414 | - | mRNA | Lalb_Chr02g0140571 | Putative tetratricopeptide-like helical domain-containing protein |
| Chr02 | 42027 | 43644 | - | mRNA | Lalb_Chr02g0140581 | Putative photosystem II Pbs27 |
| Chr02 | 43646 | 44501 | - | mRNA | Lalb_Chr02g0140591 | Putative Beta-grasp domain-containing protein |
| Chr02 | 45159 | 51852 | + | mRNA | Lalb_Chr02g0140601 | Putative XS domain-containing protein |
| Chr02 | 52881 | 53974 | - | mRNA | Lalb_Chr02g0140611 | Putative ferredoxin [2Fe-2S], plant, Beta-grasp domain-containing protein |
| Chr02 | 56127 | 59994 | + | mRNA | Lalb_Chr02g0140621 | Putative cysteine synthase |
| Chr02 | 60327 | 61780 | - | mRNA | Lalb_Chr02g0140631 | hypothetical protein |
| Chr02 | 63647 | 64814 | - | mRNA | Lalb_Chr02g0140641 | putative protein |
| Chr02 | 71021 | 71658 | + | mRNA | Lalb_Chr02g0140651 | Putative bifunctional inhibitor/plant lipid transfer protein/seed storage helical |
| Chr02 | 72024 | 74595 | - | mRNA | Lalb_Chr02g0140661 | Putative RNA-directed DNA polymerase |
| Chr02 | 74891 | 76092 | + | mRNA | Lalb_Chr02g0140671 | hypothetical protein |
| Chr02 | 76707 | 77026 | - | mRNA | Lalb_Chr02g0140681 | hypothetical protein |
| Chr02 | 77432 | 80320 | - | mRNA | Lalb_Chr02g0140691 | Putative IQ motif, EF-hand binding protein |
| Chr02 | 80809 | 83232 | + | mRNA | Lalb_Chr02g0140701 | hypothetical protein |
| Chr02 | 83535 | 86318 | - | mRNA | Lalb_Chr02g0140711 | Putative perakine reductase |
| Chr02 | 87577 | 90201 | + | mRNA | Lalb_Chr02g0140721 | putative protein |
| Chr02 | 91277 | 99850 | + | mRNA | Lalb_Chr02g0140731 | Putative mitogen-activated protein kinase kinase kinase TKL-Pl-2 family |
| Chr02 | 102072 | 102164 | + | ncRNA | Lalb_Chr02g0140741 | microRNA MIR167_1 |
| Chr02 | 102166 | 102460 | + | mRNA | Lalb_Chr02g0140751 | hypothetical protein |
| Chr02 | 110807 | 114889 | + | mRNA | Lalb_Chr02g0140761 | Putative sulfate adenylyltransferase |
| Chr02 | 115177 | 117050 | - | mRNA | Lalb_Chr02g0140771 | Putative CHORD domain-containing protein |
| Chr02 | 121021 | 122804 | + | mRNA | Lalb_Chr02g0140781 | Putative exostosin |
| Chr02 | 123858 | 124847 | + | mRNA | Lalb_Chr02g0140791 | hypothetical protein |
| Chr02 | 126142 | 128058 | - | mRNA | Lalb_Chr02g0140801 | Putative alpha-L-fucosidase |
| Chr02 | 131978 | 133545 | + | mRNA | Lalb_Chr02g0140811 | Putative oxidoreductase, Very-long-chain 3-oxoacyl-CoA synthase |
| Chr02 | 135264 | 137980 | + | mRNA | Lalb_Chr02g0140821 | Putative cucumisin |
| Chr02 | 137983 | 140140 | + | mRNA | Lalb_Chr02g0140831 | Putative protein kinase RLK-Pelle-RLCK-XI family |
| Chr02 | 142052 | 144278 | + | mRNA | Lalb_Chr02g0140841 | Putative non-specific protein-tyrosine kinase RLK-Pelle-RLCK-XV family |
| Chr02 | 144360 | 145473 | + | mRNA | Lalb_Chr02g0140851 | Putative carboxylesterase |
| Chr02 | 145475 | 151724 | - | mRNA | Lalb_Chr02g0140861 | Phosphoglucomutase |
| Chr02 | 152497 | 158544 | - | mRNA | Lalb_Chr02g0140871 | Putative GDP-fucose protein O-fucosyltransferase |
| Chr02 | 152571 | 152634 | - | marker | TP23903 |  |
| Chr02 | 159434 | 162015 | - | mRNA | Lalb_Chr02g0140881 | putative protein |
| Chr02 | 170766 | 173322 | - | mRNA | Lalb_Chr02g0140891 | Putative transcription factor MADS-MIKC family |
| Chr02 | 177635 | 180305 | - | mRNA | Lalb_Chr02g0140901 | Putative molybdenum cofactor sulfurtransferase |
| Chr02 | 188230 | 191891 | + | mRNA | Lalb_Chr02g0140911 | Putative S-adenosyl-L-methionine-dependent methyltransferase |
| Chr02 | 204087 | 210069 | + | mRNA | Lalb_Chr02g0140921 | Putative transcription factor %26 lipid binding HD-SAD family |
| Chr02 | 220845 | 222232 | - | mRNA | Lalb_Chr02g0140931 | hypothetical protein |
| Chr02 | 222611 | 222990 | + | mRNA | Lalb_Chr02g0140941 | Putative RNA-directed DNA polymerase |
| Chr02 | 224196 | 228511 | + | mRNA | Lalb_Chr02g0140951 | Putative protein kinase RLK-Pelle-LRR-Xb-1 family |
| Chr02 | 229028 | 230129 | - | mRNA | Lalb_Chr02g0140961 | Putative 6-phospho-3-hexuloisomerase |
| Chr02 | 231923 | 232723 | - | mRNA | Lalb_Chr02g0140971 | hypothetical protein |
| Chr02 | 233900 | 234260 | + | mRNA | Lalb_Chr02g0140981 | hypothetical protein |
| Chr02 | 234846 | 237285 | - | mRNA | Lalb_Chr02g0140991 | Putative cytochrome b5-like heme/steroid binding domain-containing protein |
| Chr02 | 240818 | 244136 | + | mRNA | Lalb_Chr02g0141001 | Putative transcription factor MYB-HB-like family |
| Chr02 | 245426 | 247032 | + | mRNA | Lalb_Chr02g0141011 | Putative transcription factor MYB-HB-like family |
| Chr02 | 247538 | 250011 | - | mRNA | Lalb_Chr02g0141021 | Putative remorin |
| Chr02 | 252124 | 258655 | + | mRNA | Lalb_Chr02g0141031 | Putative EEIG1/EHBP1 domain-containing protein |
| Chr02 | 259732 | 260668 | + | ncRNA | Lalb_Chr02g0141041 |  |
| Chr02 | 261859 | 264950 | + | mRNA | Lalb_Chr02g0141051 | Putative pentatricopeptide |
| Chr02 | 265764 | 269862 | - | mRNA | Lalb_Chr02g0141061 | Putative transcription factor interactor and regulator CCHC(Zn) family |
| Chr02 | 272404 | 274729 | + | mRNA | Lalb_Chr02g0141071 | Putative purine-nucleoside phosphorylase |
| Chr02 | 275044 | 288985 | - | mRNA | Lalb_Chr02g0141081 | Putative tripeptidyl-peptidase II |
| Chr02 | 289780 | 295739 | - | mRNA | Lalb_Chr02g0141091 | Putative pectinacetylesterase/NOTUM |
| Chr02 | 296863 | 299943 | + | mRNA | Lalb_Chr02g0141101 | Putative presenilin/signal peptide peptidase |
| Chr02 | 300763 | 305200 | + | mRNA | Lalb_Chr02g0141111 | Putative transcription factor Nin-like family |
| Chr02 | 305515 | 308179 | - | mRNA | Lalb_Chr02g0141121 | Putative ribosomal protein L36e |
| Chr02 | 308194 | 310975 | + | mRNA | Lalb_Chr02g0141131 | Putative triacylglycerol lipase |
| Chr02 | 312171 | 319233 | + | mRNA | Lalb_Chr02g0141141 | Putative transcription factor C2H2 family |
| Chr02 | 319931 | 320465 | - | mRNA | Lalb_Chr02g0141151 | hypothetical protein |
| Chr02 | 320467 | 324967 | + | mRNA | Lalb_Chr02g0141161 | Short-chain dehydrogenase TIC 32 |
| Chr02 | 327695 | 328847 | + | mRNA | Lalb_Chr02g0141171 | Putative sec-independent protein translocase protein TatA/B/E |
| Chr02 | 329638 | 336391 | - | mRNA | Lalb_Chr02g0141181 | Putative mRNA (2'-O-methyladenosine-N(6)-)-methyltransferase |
| Chr02 | 336955 | 338787 | - | mRNA | Lalb_Chr02g0141191 | putative protein |
| Chr02 | 342401 | 347269 | + | mRNA | Lalb_Chr02g0141201 | Putative proline--tRNA ligase |
| Chr02 | 347412 | 349760 | - | mRNA | Lalb_Chr02g0141211 | putative protein |
| Chr02 | 349828 | 352760 | + | mRNA | Lalb_Chr02g0141221 | Putative protein kinase RLK-Pelle-CrRLK1L-1 family |
| Chr02 | 354381 | 355328 | - | mRNA | Lalb_Chr02g0141231 | hypothetical protein |
| Chr02 | 355330 | 356739 | - | mRNA | Lalb_Chr02g0141241 | hypothetical protein |
| Chr02 | 358295 | 360261 | + | mRNA | Lalb_Chr02g0141251 | Putative galacturan 1,4-alpha-galacturonidase |
| Chr02 | 361594 | 362728 | + | mRNA | Lalb_Chr02g0141261 | Putative heavy metal-associated domain, HMA |
| Chr02 | 363175 | 364765 | + | mRNA | Lalb_Chr02g0141271 | Putative thiomorpholine-carboxylate dehydrogenase |
| Chr02 | 366190 | 370031 | + | mRNA | Lalb_Chr02g0141281 | Putative 43kDa postsynaptic protein |
| Chr02 | 371985 | 374379 | - | mRNA | Lalb_Chr02g0141291 | Putative pigment precursor permease, P-loop containing nucleoside triphosphate hydrolase |
| Chr02 | 379493 | 381066 | - | mRNA | Lalb_Chr02g0141301 | hypothetical protein |
| Chr02 | 384415 | 386074 | + | mRNA | Lalb_Chr02g0141311 | putative protein |
| Chr02 | 386195 | 389718 | - | mRNA | Lalb_Chr02g0141321 | Putative F-box domain-containing protein |
| Chr02 | 392301 | 395995 | + | mRNA | Lalb_Chr02g0141331 | Putative voltage-dependent anion channel |
| Chr02 | 396116 | 397373 | - | mRNA | Lalb_Chr02g0141341 | hypothetical protein |
| Chr02 | 399163 | 401999 | - | mRNA | Lalb_Chr02g0141351 | Putative CAAX amino terminal protease |
| Chr02 | 402709 | 404007 | - | mRNA | Lalb_Chr02g0141361 | Putative chitinase |
| Chr02 | 405340 | 418022 | + | mRNA | Lalb_Chr02g0141371 | Putative chromatin regulator PHD family |
| Chr02 | 430343 | 432434 | + | mRNA | Lalb_Chr02g0141381 | Putative Extensin domain-containing protein |
| Chr02 | 437705 | 439779 | - | mRNA | Lalb_Chr02g0141391 | Putative transcription factor bZIP family |
| Chr02 | 445842 | 446909 | - | mRNA | Lalb_Chr02g0141401 | hypothetical protein |
| Chr02 | 451867 | 453656 | - | mRNA | Lalb_Chr02g0141411 | Putative ABA/WDS induced protein |
| Chr02 | 461711 | 463159 | - | mRNA | Lalb_Chr02g0141421 | Putative proline--tRNA ligase |
| Chr02 | 463162 | 463411 | + | ncRNA | Lalb_Chr02g0141431 |  |
| Chr02 | 463901 | 467415 | - | mRNA | Lalb_Chr02g0141441 | Putative protein-disulfide reductase |
| Chr02 | 469528 | 470021 | + | ncRNA | Lalb_Chr02g0141461 |  |
| Chr02 | 470023 | 472456 | + | mRNA | Lalb_Chr02g0141471 | Putative ribosomal protein L3 |
| Chr02 | 494562 | 500566 | + | mRNA | Lalb_Chr02g0141481 | Putative RNA recognition motif domain-containing protein |
| Chr02 | 500751 | 501773 | - | mRNA | Lalb_Chr02g0141491 | Putative pectinesterase inhibitor domain-containing protein |
| Chr02 | 501106 | 501189 | + | marker | TP229924 |  |
| Chr02 | 503325 | 508771 | + | mRNA | Lalb_Chr02g0141501 | Putative histone-lysine N-methyltransferase chromatin remodeling SET family |
| Chr02 | 508773 | 511310 | + | mRNA | Lalb_Chr02g0141511 | Putative protein kinase RLK-Pelle-LRR-Xb-1 family |
| Chr02 | 511611 | 515870 | - | mRNA | Lalb_Chr02g0141521 | Putative protein-serine/threonine kinase CMGC-CDK-CRK7-CDK9 family |
| Chr02 | 516640 | 521409 | - | mRNA | Lalb_Chr02g0141531 | hypothetical protein |
| Chr02 | 525216 | 529295 | - | mRNA | Lalb_Chr02g0141541 | putative protein |
| Chr02 | 530028 | 530651 | - | mRNA | Lalb_Chr02g0141551 | Putative ribosomal protein S13 |
| Chr02 | 530688 | 532388 | - | mRNA | Lalb_Chr02g0141561 | Putative ribosomal protein S13 |
| Chr02 | 533299 | 544011 | - | mRNA | Lalb_Chr02g0141571 | putative protein |
| Chr02 | 545656 | 552963 | + | mRNA | Lalb_Chr02g0141581 | Putative metallocarboxypeptidase D |
| Chr02 | 550608 | 550817 | + | marker | TP222136 |  |
| Chr02 | 552965 | 557685 | - | mRNA | Lalb_Chr02g0141591 | Putative mitochondrial carrier protein |
| Chr02 | 558546 | 561638 | + | mRNA | Lalb_Chr02g0141601 | Putative phosphotransferase with an alcohol group as acceptor |
| Chr02 | 563559 | 567825 | + | mRNA | Lalb_Chr02g0141611 | Putative protein kinase RLK-Pelle-LRR-XI-1 family |
| Chr02 | 569176 | 571847 | - | mRNA | Lalb_Chr02g0141621 | Putative hypoxanthine phosphoribosyltransferase |
| Chr02 | 572017 | 575956 | - | mRNA | Lalb_Chr02g0141631 | putative protein |
| Chr02 | 576192 | 580762 | + | mRNA | Lalb_Chr02g0141641 | Putative pseudouridylate synthase, Isomerase |
| Chr02 | 580812 | 583306 | + | mRNA | Lalb_Chr02g0141651 | Putative tRNA pseudouridine(38/39) synthase |
| Chr02 | 583586 | 585915 | - | mRNA | Lalb_Chr02g0141661 | Putative sphingolipid C4-monooxygenase |
| Chr02 | 586957 | 592466 | + | mRNA | Lalb_Chr02g0141671 | Putative RNA recognition motif domain, cleavage stimulation factor subunit 2, hinge |
| Chr02 | 593044 | 595692 | - | mRNA | Lalb_Chr02g0141681 | Putative laccase |
| Chr02 | 598557 | 599715 | - | mRNA | Lalb_Chr02g0141691 | Putative elicitor peptide |
| Chr02 | 603077 | 608756 | + | mRNA | Lalb_Chr02g0141701 | Putative transferase, protein kinase RLK-Pelle-LRR-II family |
| Chr02 | 609345 | 612308 | - | mRNA | Lalb_Chr02g0141711 | Putative xylogalacturonan beta-1,3-xylosyltransferase |
| Chr02 | 612696 | 616083 | + | mRNA | Lalb_Chr02g0141721 | Putative glycoprotein 3-alpha-L-fucosyltransferase |
| Chr02 | 616559 | 621481 | + | mRNA | Lalb_Chr02g0141731 | Putative thioredoxin-like protein |
| Chr02 | 623954 | 628508 | + | mRNA | Lalb_Chr02g0141741 | Putative transcription factor C2H2 family |
| Chr02 | 625038 | 625198 | + | marker | TP272531 |  |
| Chr02 | 632539 | 633669 | - | mRNA | Lalb_Chr02g0141751 | putative protein |
| Chr02 | 634738 | 636770 | - | mRNA | Lalb_Chr02g0141761 | Putative ABC-2 type transporter |
| Chr02 | 635819 | 635884 | + | marker | TP47110 |  |
| Chr02 | 637387 | 641111 | - | mRNA | Lalb_Chr02g0141771 | Putative polar-amino-acid-transporting ATPase |
| Chr02 | 643521 | 645028 | - | mRNA | Lalb_Chr02g0141781 | hypothetical protein |
| Chr02 | 646674 | 647385 | + | mRNA | Lalb_Chr02g0141791 | Putative myc-type, basic helix-loop-helix (bHLH) domain-containing protein |
| Chr02 | 649109 | 652405 | + | mRNA | Lalb_Chr02g0141801 | Putative protein kinase CAMK-CDPK family |
| Chr02 | 653066 | 656019 | - | mRNA | Lalb_Chr02g0141811 | Putative SNARE associated golgi family protein |
| Chr02 | 657808 | 659742 | - | mRNA | Lalb_Chr02g0141821 | Putative transcription factor NAM family |
| Chr02 | 667618 | 672455 | + | mRNA | Lalb_Chr02g0141831 | putative protein |
| Chr02 | 672520 | 673275 | + | mRNA | Lalb_Chr02g0141841 | putative protein |
| Chr02 | 674316 | 678800 | - | mRNA | Lalb_Chr02g0141851 | Putative dihydrolipoyllysine-residue acetyltransferase |
| Chr02 | 685173 | 686415 | + | mRNA | Lalb_Chr02g0141861 | Putative gibberellin regulated protein |
| Chr02 | 688194 | 694435 | - | mRNA | Lalb_Chr02g0141871 | Putative alcohol O-acetyltransferase |
| Chr02 | 695417 | 698833 | + | mRNA | Lalb_Chr02g0141881 | Putative RNA recognition motif domain-containing protein |
| Chr02 | 700447 | 706033 | + | mRNA | Lalb_Chr02g0141891 | Putative beta-fructofuranosidase |
| Chr02 | 715369 | 717050 | + | mRNA | Lalb_Chr02g0141901 | Putative transcription factor AS2-LOB family |
| Chr02 | 719885 | 721958 | + | mRNA | Lalb_Chr02g0141911 | Putative ATP synthase, F0 complex, subunit D |
| Chr02 | 722280 | 729142 | - | mRNA | Lalb_Chr02g0141921 | Putative calcium binding protein |
| Chr02 | 730542 | 735109 | - | mRNA | Lalb_Chr02g0141931 | Putative IQ motif, EF-hand binding protein |
| Chr02 | 736853 | 738048 | - | mRNA | Lalb_Chr02g0141941 | hypothetical protein |
| Chr02 | 739464 | 742067 | + | mRNA | Lalb_Chr02g0141951 | Elongation factor 1-alpha |
| Chr02 | 742750 | 750876 | + | mRNA | Lalb_Chr02g0141961 | Putative Taxilin family |
| Chr02 | 752494 | 755456 | + | mRNA | Lalb_Chr02g0141971 | hypothetical protein |
| Chr02 | 757146 | 765269 | - | mRNA | Lalb_Chr02g0141981 | Putative transcription factor MYB/SANT family |
| Chr02 | 771801 | 774162 | + | mRNA | Lalb_Chr02g0141991 | Putative tetraspanin/Peripherin |
| Chr02 | 774362 | 775064 | - | ncRNA | Lalb_Chr02g0142001 |  |
| Chr02 | 776293 | 779620 | + | mRNA | Lalb_Chr02g0142011 | Putative tetratricopeptide-like helical domain, DYW domain-containing protein |
| Chr02 | 780762 | 781383 | + | mRNA | Lalb_Chr02g0142021 | hypothetical protein |
| Chr02 | 787524 | 787871 | + | mRNA | Lalb_Chr02g0142041 | hypothetical protein |
| Chr02 | 788785 | 793366 | - | mRNA | Lalb_Chr02g0142051 | Putative chromatin regulator PHD family |
| Chr02 | 795009 | 795294 | + | mRNA | Lalb_Chr02g0142061 | hypothetical protein |
| Chr02 | 796506 | 804123 | - | mRNA | Lalb_Chr02g0142071 | Putative cadmium-transporting ATPase |
| Chr02 | 804278 | 806155 | + | mRNA | Lalb_Chr02g0142081 | Putative tetratricopeptide-like helical domain-containing protein |
| Chr02 | 806246 | 807838 | + | mRNA | Lalb_Chr02g0142091 | Putative tetratricopeptide-like helical domain, DYW domain-containing protein |
| Chr02 | 816755 | 817613 | + | ncRNA | Lalb_Chr02g0142101 |  |
| Chr02 | 817615 | 817700 | + | ncRNA | Lalb_Chr02g0142111 | mir-160 microRNA precursor family |
| Chr02 | 817704 | 820483 | + | mRNA | Lalb_Chr02g0142121 | hypothetical protein |
| Chr02 | 821219 | 829695 | - | mRNA | Lalb_Chr02g0142131 | Putative aminodeoxychorismate synthase |
| Chr02 | 830260 | 835932 | + | mRNA | Lalb_Chr02g0142141 | Putative chorismate synthase |
| Chr02 | 840614 | 840734 | + | ncRNA | Lalb_Chr02g0142151 | 5S ribosomal RNA |
| Chr02 | 841632 | 846953 | - | mRNA | Lalb_Chr02g0142161 | Putative phospholipase A(2) |
| Chr02 | 848969 | 851806 | - | mRNA | Lalb_Chr02g0142171 | Putative major intrinsic protein |
| Chr02 | 853399 | 853829 | - | mRNA | Lalb_Chr02g0142181 | Putative major intrinsic protein |
| Chr02 | 858592 | 862113 | + | mRNA | Lalb_Chr02g0142191 | Putative reverse transcriptase domain, domain X |
| Chr02 | 863385 | 866546 | + | mRNA | Lalb_Chr02g0142201 | Putative transcription regulator GNAT family |
| Chr02 | 866719 | 875659 | - | mRNA | Lalb_Chr02g0142211 | Putative phosphotransferase (phosphomutase) |
| Chr02 | 876032 | 879840 | - | mRNA | Lalb_Chr02g0142221 | Putative transcription factor C2C2-GATA family |
| Chr02 | 885014 | 892032 | - | mRNA | Lalb_Chr02g0142231 | Putative protein ENHANCED DISEASE RESISTANCE 2, PH domain, START |
| Chr02 | 892832 | 897182 | - | mRNA | Lalb_Chr02g0142241 | Putative dolichyl-diphosphooligosaccharide--protein glycotransferase |
| Chr02 | 897779 | 900934 | - | mRNA | Lalb_Chr02g0142251 | Putative C2 domain, synaptotagmin-like mitochondrial-lipid-binding domain-containing protein |
| Chr02 | 900936 | 905804 | + | mRNA | Lalb_Chr02g0142261 | Putative phenylalanine ammonia-lyase |
| Chr02 | 906458 | 906845 | - | mRNA | Lalb_Chr02g0142271 | Putative small auxin-up RNA |
| Chr02 | 911112 | 918742 | + | mRNA | Lalb_Chr02g0142281 | Putative [Histone H3]-lysine-36 demethylase |
| Chr02 | 926229 | 927560 | + | mRNA | Lalb_Chr02g0142291 | Putative transcription factor AS2-LOB family |
| Chr02 | 935238 | 936598 | - | mRNA | Lalb_Chr02g0142301 | Putative transcription factor AS2-LOB family |
| Chr02 | 940298 | 940897 | - | mRNA | Lalb_Chr02g0142311 | Putative transcription factor C2H2 family |
| Chr02 | 942739 | 942946 | + | mRNA | Lalb_Chr02g0142321 | hypothetical protein |
| Chr02 | 944983 | 946767 | - | mRNA | Lalb_Chr02g0142331 | Putative cytochrome P450 |
| Chr02 | 952571 | 956359 | + | mRNA | Lalb_Chr02g0142341 | Putative flagellum site-determining protein YlxH/ Fe-S cluster assembling factor NBP35 |
| Chr02 | 957252 | 961110 | + | mRNA | Lalb_Chr02g0142351 | Putative 5-dehydro-2-deoxygluconokinase |
| Chr02 | 961773 | 966881 | - | mRNA | Lalb_Chr02g0142361 | Putative K domain-containing protein |
| Chr02 | 968103 | 971304 | - | mRNA | Lalb_Chr02g0142371 | Putative transcription factor WRKY family |
| Chr02 | 974416 | 975241 | + | mRNA | Lalb_Chr02g0142381 | hypothetical protein |
| Chr02 | 975600 | 976491 | - | mRNA | Lalb_Chr02g0142391 | Putative RNA-directed DNA polymerase |
| Chr02 | 982487 | 986283 | - | mRNA | Lalb_Chr02g0142401 | Putative transcription factor C2H2 family |
| Chr02 | 994875 | 999484 | + | mRNA | Lalb_Chr02g0142411 | Putative F-box domain, leucine-rich repeat domain, L domain-containing protein |
| Chr02 | 997603 | 997707 | - | marker | TP291372 |  |
| Chr02 | 1002794 | 1003936 | + | mRNA | Lalb_Chr02g0142431 | Putative transcription factor interactor and regulator AUX-IAA family |
| Chr02 | 1004572 | 1006948 | - | mRNA | Lalb_Chr02g0142441 | Putative primosome PriB/single-strand DNA-binding protein |
| Chr02 | 1007763 | 1011575 | - | mRNA | Lalb_Chr02g0142451 | hypothetical protein |
| Chr02 | 1011737 | 1013289 | + | mRNA | Lalb_Chr02g0142461 | Putative transcription factor Hap3/NF-YB family |
| Chr02 | 1015152 | 1017872 | + | mRNA | Lalb_Chr02g0142471 | Putative 2-methyl-6-phytyl-1,4-hydroquinone methyltransferase |
| Chr02 | 1018550 | 1019729 | + | mRNA | Lalb_Chr02g0142481 | Putative EF-hand domain pair protein |
| Chr02 | 1020945 | 1021055 | - | ncRNA | Lalb_Chr02g0142491 | microRNA MIR167_1 |
| Chr02 | 1023701 | 1024699 | - | ncRNA | Lalb_Chr02g0142501 |  |
| Chr02 | 1024906 | 1025976 | - | ncRNA | Lalb_Chr02g0142511 |  |
| Chr02 | 1026837 | 1033065 | + | mRNA | Lalb_Chr02g0142521 | Putative [Histone H3]-lysine-36 demethylase |
| Chr02 | 1034449 | 1037339 | + | mRNA | Lalb_Chr02g0142531 | putative protein |
| Chr02 | 1037524 | 1038739 | - | mRNA | Lalb_Chr02g0142541 | Putative pleckstrin-like, plant |
| Chr02 | 1038747 | 1039442 | + | mRNA | Lalb_Chr02g0142551 | hypothetical protein |
| Chr02 | 1039965 | 1041388 | - | mRNA | Lalb_Chr02g0142561 | putative protein |
| Chr02 | 1042846 | 1044641 | - | mRNA | Lalb_Chr02g0142571 | Putative isopenicillin N synthase |
| Chr02 | 1046969 | 1048212 | + | mRNA | Lalb_Chr02g0142581 | hypothetical protein |
| Chr02 | 1052350 | 1057982 | + | mRNA | Lalb_Chr02g0142591 | Putative SLC26A/SulP transporter |
| Chr02 | 1058109 | 1066489 | - | mRNA | Lalb_Chr02g0142601 | Putative protein Networked (NET), actin-binding (NAB) |
| Chr02 | 1060908 | 1060973 | - | marker | TP237794 |  |
| Chr02 | 1061852 | 1062091 | + | marker | TP446132 |  |
| Chr02 | 1076676 | 1082549 | + | mRNA | Lalb_Chr02g0142611 | Putative phosphoric monoester hydrolase |
| Chr02 | 1084291 | 1084736 | - | mRNA | Lalb_Chr02g0142621 | hypothetical protein |
| Chr02 | 1087995 | 1090356 | - | mRNA | Lalb_Chr02g0142631 | hypothetical protein |
| Chr02 | 1091834 | 1092908 | + | mRNA | Lalb_Chr02g0142641 | Putative HSP20-like chaperone |
| Chr02 | 1094305 | 1095435 | + | mRNA | Lalb_Chr02g0142651 | Putative Seed maturation protein |
| Chr02 | 1098717 | 1099233 | - | ncRNA | Lalb_Chr02g0142661 |  |
| Chr02 | 1099235 | 1099567 | + | mRNA | Lalb_Chr02g0142671 | Putative transcription factor OFP family |
| Chr02 | 1100614 | 1108035 | - | mRNA | Lalb_Chr02g0142681 | Putative histone acetyltransferase chromatin regulator PHD family |
| Chr02 | 1108567 | 1109388 | - | mRNA | Lalb_Chr02g0142691 | hypothetical protein |
| Chr02 | 1112391 | 1113794 | + | mRNA | Lalb_Chr02g0142701 | putative protein |
| Chr02 | 1114548 | 1116039 | - | mRNA | Lalb_Chr02g0142711 | putative protein |
| Chr02 | 1116182 | 1120066 | + | mRNA | Lalb_Chr02g0142721 | Putative methionine N(alpha)-acetyltransferase NatB transcription regulator GNAT family |
| Chr02 | 1120166 | 1125312 | - | mRNA | Lalb_Chr02g0142731 | hypothetical protein |
| Chr02 | 1131027 | 1134857 | - | mRNA | Lalb_Chr02g0142741 | Putative ribosome recycling factor |
| Chr02 | 1135210 | 1135930 | + | mRNA | Lalb_Chr02g0142751 | Putative cupredoxin |
| Chr02 | 1137909 | 1139639 | + | mRNA | Lalb_Chr02g0142761 | Putative phospholipase A(2) |
| Chr02 | 1145626 | 1147477 | + | mRNA | Lalb_Chr02g0142771 | Putative aldolase-type TIM barrel |
| Chr02 | 1147479 | 1152515 | + | mRNA | Lalb_Chr02g0142781 | Putative dynamin GTPase |
| Chr02 | 1154388 | 1155533 | + | mRNA | Lalb_Chr02g0142791 | Putative The fantastic four family protein |
| Chr02 | 1162646 | 1166297 | - | mRNA | Lalb_Chr02g0142801 | Putative F-box domain-containing protein |
| Chr02 | 1167579 | 1169197 | - | mRNA | Lalb_Chr02g0142811 | Putative nepenthesin |
| Chr02 | 1171088 | 1172707 | - | mRNA | Lalb_Chr02g0142821 | Putative nepenthesin |
| Chr02 | 1173694 | 1177229 | - | mRNA | Lalb_Chr02g0142831 | Putative R3H domain-containing protein |
| Chr02 | 1177716 | 1179051 | - | mRNA | Lalb_Chr02g0142841 | Putative transferase |
| Chr02 | 1188771 | 1190797 | - | mRNA | Lalb_Chr02g0142851 | Putative plant organelle RNA recognition domain-containing protein |
| Chr02 | 1191813 | 1196416 | + | mRNA | Lalb_Chr02g0142861 | hypothetical protein |
| Chr02 | 1196953 | 1198884 | - | mRNA | Lalb_Chr02g0142871 | Putative glutathione peroxidase |
| Chr02 | 1200566 | 1213520 | - | mRNA | Lalb_Chr02g0142881 | Putative PWWP domain-containing protein |
| Chr02 | 1215468 | 1218584 | - | mRNA | Lalb_Chr02g0142891 | Putative U6 snRNA-associated Sm-like protein LSm2 |
| Chr02 | 1219123 | 1222776 | - | mRNA | Lalb_Chr02g0142901 | Putative chromatin remodeling %26 transcription regulator BTB-POZ-MATH family |
| Chr02 | 1232161 | 1239001 | + | mRNA | Lalb_Chr02g0142911 | Putative oxoglutarate/iron-dependent dioxygenase, alpha-ketoglutarate-dependent dioxygenase AlkB |
| Chr02 | 1238559 | 1238657 | - | marker | TP88533 |  |
| Chr02 | 1238632 | 1238736 | + | marker | TP38227 |  |
| Chr04 | 15399105 | 15399204 | + | marker | TP149038 |  |
| Chr04 | 15402379 | 15403874 | + | mRNA | Lalb_Chr04g0262821 | Putative acyl-CoA hydrolase |
| Chr04 | 15410610 | 15414222 | - | mRNA | Lalb_Chr04g0262831 | Putative proton-dependent oligopeptide transporter family, major facilitator superfamily |
| Chr04 | 15420657 | 15424468 | - | mRNA | Lalb_Chr04g0262841 | Putative peroxyureidoacrylate/ureidoacrylate amidohydrolase |
| Chr04 | 15426840 | 15432426 | - | mRNA | Lalb_Chr04g0262851 | Putative transcription factor bHLH family |
| Chr04 | 15439271 | 15440510 | + | mRNA | Lalb_Chr04g0262861 | Putative F-box associated interaction domain-containing protein |
| Chr04 | 15443013 | 15447843 | - | mRNA | Lalb_Chr04g0262871 | hypothetical protein |
| Chr04 | 15456903 | 15458177 | + | mRNA | Lalb_Chr04g0262881 | Putative transcription factor AP2-EREBP family |
| Chr04 | 15485089 | 15488379 | - | mRNA | Lalb_Chr04g0262901 | Putative casparian strip membrane protein |
| Chr04 | 15489512 | 15490763 | - | mRNA | Lalb_Chr04g0262911 | hypothetical protein |
| Chr04 | 15497934 | 15500313 | - | mRNA | Lalb_Chr04g0262921 | Putative transcription factor C2C2-GATA family |
| Chr04 | 15510058 | 15511285 | - | ncRNA | Lalb_Chr04g0262931 |  |
| Chr04 | 15513721 | 15515780 | - | mRNA | Lalb_Chr04g0262941 | hypothetical protein |
| Chr04 | 15516900 | 15520613 | + | mRNA | Lalb_Chr04g0262951 | Putative oxoglutarate/iron-dependent dioxygenase, non-haem dioxygenase domain-containing protein |
| Chr04 | 15523518 | 15528315 | + | mRNA | Lalb_Chr04g0262961 | Putative malate dehydrogenase (oxaloacetate-decarboxylating) (NADP(+)) |
| Chr04 | 15530914 | 15536558 | + | mRNA | Lalb_Chr04g0262971 | Putative RNA polymerase II associated factor Paf1 |
| Chr04 | 15538785 | 15542071 | + | mRNA | Lalb_Chr04g0262981 | Putative IQ motif, EF-hand binding protein |
| Chr04 | 15550490 | 15556213 | - | mRNA | Lalb_Chr04g0262991 | Putative quinol--cytochrome-c reductase, Mitochondrial processing peptidase |
| Chr04 | 15556961 | 15565467 | - | mRNA | Lalb_Chr04g0263001 | Putative DNA repair protein RadA |
| Chr04 | 15565910 | 15569305 | + | mRNA | Lalb_Chr04g0263011 | hypothetical protein |
| Chr04 | 15569765 | 15573510 | - | mRNA | Lalb_Chr04g0263021 | Putative inositol oxygenase |
| Chr04 | 15578964 | 15584895 | + | mRNA | Lalb_Chr04g0263041 | Putative vacuolar protein sorting protein 26 related protein |
| Chr04 | 15588947 | 15591532 | + | mRNA | Lalb_Chr04g0263051 | Putative glycosyl transferase, family 14 |
| Chr04 | 15591719 | 15592665 | - | mRNA | Lalb_Chr04g0263061 | Putative leucine-rich repeat domain, L domain-containing protein |
| Chr04 | 15594229 | 15596110 | - | mRNA | Lalb_Chr04g0263071 | Putative F-box domain, leucine-rich repeat domain, L domain-containing protein |
| Chr04 | 15597348 | 15608437 | - | mRNA | Lalb_Chr04g0263081 | Putative ubiquitinyl hydrolase 1 |
| Chr04 | 15617485 | 15625208 | + | mRNA | Lalb_Chr04g0263091 | Putative occludin domain-containing protein |
| Chr04 | 15629010 | 15638106 | + | mRNA | Lalb_Chr04g0263101 | Putative glutamate dehydrogenase (NADP(+)) |
| Chr04 | 15638509 | 15644040 | - | mRNA | Lalb_Chr04g0263111 | Putative ubiquitin-conjugating enzyme/RWD |
| Chr04 | 15647232 | 15652493 | + | mRNA | Lalb_Chr04g0263121 | Putative PWWP domain-containing protein |
| Chr04 | 15654472 | 15657824 | + | mRNA | Lalb_Chr04g0263131 | Putative ribosomal protein L28e |
| Chr04 | 15658293 | 15660210 | + | mRNA | Lalb_Chr04g0263141 | Putative hydrolase |
| Chr04 | 15660267 | 15662038 | - | mRNA | Lalb_Chr04g0263151 | hypothetical protein |
| Chr04 | 15664415 | 15664981 | - | mRNA | Lalb_Chr04g0263161 | Putative prenylated rab acceptor PRA1 |
| Chr04 | 15666067 | 15671449 | - | mRNA | Lalb_Chr04g0263171 | Putative RNA helicase |
| Chr04 | 15682694 | 15689301 | + | mRNA | Lalb_Chr04g0263181 | Putative beta-galactosidase |
| Chr04 | 15691433 | 15695552 | - | mRNA | Lalb_Chr04g0263191 | Putative nonaspanin (TM9SF), major facilitator superfamily domain-containing protein |
| Chr04 | 15697808 | 15706778 | + | mRNA | Lalb_Chr04g0263201 | Putative glucose-methanol-choline oxidoreductase, FAD/NAD(P)-binding domain-containing protein |
| Chr04 | 15707212 | 15714600 | + | mRNA | Lalb_Chr04g0263211 | Putative chaperonin Cpn60/TCP-1 family, groEL-like apical domain, groEL-like equatorial |
| Chr04 | 15715544 | 15716405 | - | mRNA | Lalb_Chr04g0263221 | Putative transcription factor AS2-LOB family |
| Chr04 | 15717573 | 15722614 | - | mRNA | Lalb_Chr04g0263231 | hypothetical protein |
| Chr04 | 15722861 | 15724697 | + | mRNA | Lalb_Chr04g0263241 | hypothetical protein |
| Chr04 | 15725570 | 15729961 | - | mRNA | Lalb_Chr04g0263251 | Putative F-box domain, leucine-rich repeat domain, L domain-containing protein |
| Chr04 | 15732060 | 15737033 | + | mRNA | Lalb_Chr04g0263261 | Putative beta-fructofuranosidase |
| Chr04 | 15737063 | 15739052 | + | mRNA | Lalb_Chr04g0263271 | hypothetical protein |
| Chr04 | 15740095 | 15746187 | + | mRNA | Lalb_Chr04g0263281 | Putative protein kinase CK1-CK1 family |
| Chr04 | 15752195 | 15756078 | + | mRNA | Lalb_Chr04g0263291 | Putative linoleate 9S-lipoxygenase |
| Chr04 | 15756820 | 15765841 | + | mRNA | Lalb_Chr04g0263301 | Putative dihydrolipoyllysine-residue acetyltransferase |
| Chr04 | 15766644 | 15769256 | + | mRNA | Lalb_Chr04g0263311 | Putative membrane alanyl aminopeptidase |
| Chr04 | 15769814 | 15774878 | - | mRNA | Lalb_Chr04g0263321 | Putative chromatin regulator PHD family |
| Chr04 | 15776188 | 15780527 | - | mRNA | Lalb_Chr04g0263331 | Putative transcription factor Hap2/NF-YA family |
| Chr04 | 15792854 | 15797434 | - | mRNA | Lalb_Chr04g0263341 | Putative transferase |
| Chr04 | 15813673 | 15817548 | + | mRNA | Lalb_Chr04g0263351 | Putative brevis radix (BRX) domain, transcription factor BREVIS RADIX domain-containing protein |
| Chr04 | 15818916 | 15828087 | + | mRNA | Lalb_Chr04g0263361 | Putative GBF-interacting protein |
| Chr04 | 15827739 | 15827830 | - | marker | TP416765 |  |
| Chr04 | 15834554 | 15836099 | + | mRNA | Lalb_Chr04g0263371 | hypothetical protein |
| Chr04 | 15839889 | 15841827 | + | mRNA | Lalb_Chr04g0263381 | Putative heavy metal-associated domain, HMA |
| Chr04 | 15844299 | 15847096 | + | mRNA | Lalb_Chr04g0263391 | Putative thioredoxin-like protein |
| Chr04 | 15848658 | 15855210 | - | mRNA | Lalb_Chr04g0263401 | Putative GTP binding protein, second |
| Chr04 | 15856209 | 15864688 | - | mRNA | Lalb_Chr04g0263411 | Putative transcription factor MYB-HB-like family |
| Chr04 | 15867245 | 15870326 | + | mRNA | Lalb_Chr04g0263421 | Putative NAD(P)-binding domain-containing protein |
| Chr04 | 15871399 | 15873468 | - | mRNA | Lalb_Chr04g0263431 | Putative protein EARLY FLOWERING 4 |
| Chr04 | 15875932 | 15881971 | + | mRNA | Lalb_Chr04g0263441 | Putative transcription factor Hap3/NF-YB family |
| Chr04 | 15876351 | 15876550 | - | marker | TP3712 |  |
| Chr04 | 15876358 | 15876550 | + | marker | TP364001 |  |
| Chr04 | 15882720 | 15884295 | + | mRNA | Lalb_Chr04g0263451 | Putative transcription factor MYB-HB-like family |
| Chr04 | 15889971 | 15890430 | + | mRNA | Lalb_Chr04g0263461 | Putative transcription factor MYB-HB-like family |
| Chr04 | 15892155 | 15892673 | + | mRNA | Lalb_Chr04g0263471 | hypothetical protein |
| Chr04 | 15897903 | 15899612 | + | mRNA | Lalb_Chr04g0263481 | Putative transcription factor AP2-EREBP family |
| Chr04 | 15899615 | 15901440 | + | mRNA | Lalb_Chr04g0263491 | Putative transcription factor AP2-EREBP family |
| Chr04 | 15907405 | 15911926 | - | mRNA | Lalb_Chr04g0263501 | putative protein |
| Chr04 | 15913278 | 15914699 | + | mRNA | Lalb_Chr04g0263511 | hypothetical protein |
| Chr04 | 15916257 | 15923169 | - | mRNA | Lalb_Chr04g0263521 | Putative protein kinase RLK-Pelle-LysM family |
| Chr04 | 15928352 | 15929451 | + | mRNA | Lalb_Chr04g0263531 | Putative germin |
| Chr04 | 15930501 | 15938308 | - | mRNA | Lalb_Chr04g0263541 | Putative aminoacyltransferase, E1 ubiquitin-activating enzyme |
| Chr04 | 15938552 | 15943565 | - | mRNA | Lalb_Chr04g0263551 | Putative GTP diphosphokinase |
| Chr04 | 15950950 | 15953687 | + | mRNA | Lalb_Chr04g0263561 | Putative protein kinase RLK-Pelle-RLCK-VIIa-2 family |
| Chr04 | 15960161 | 15967894 | - | mRNA | Lalb_Chr04g0263571 | Putative linoleate 13S-lipoxygenase |
| Chr04 | 15972801 | 15973594 | - | mRNA | Lalb_Chr04g0263581 | hypothetical protein |
| Chr04 | 15979549 | 15983383 | - | mRNA | Lalb_Chr04g0263591 | Putative G-protein gamma |
| Chr04 | 15988584 | 15993568 | + | mRNA | Lalb_Chr04g0263601 | Putative [Protein-PII] uridylyltransferase |
| Chr04 | 15993811 | 15997936 | - | mRNA | Lalb_Chr04g0263611 | Putative DNAJ-containing protein |
| Chr04 | 15999141 | 16002999 | - | mRNA | Lalb_Chr04g0263621 | Putative small monomeric GTPase |
| Chr04 | 16004807 | 16008423 | + | mRNA | Lalb_Chr04g0263631 | Putative transcription factor GRAS family |
| Chr04 | 16009656 | 16013849 | - | mRNA | Lalb_Chr04g0263641 | Putative glucuronosyltransferase |
| Chr04 | 16015750 | 16020880 | - | mRNA | Lalb_Chr04g0263651 | Putative rossmann-like alpha/beta/alpha sandwich protein |
| Chr04 | 16026874 | 16028937 | + | mRNA | Lalb_Chr04g0263661 | Putative glycolipid transfer protein |
| Chr04 | 16029867 | 16033109 | + | mRNA | Lalb_Chr04g0263671 | Putative class I myosin tail domain-containing protein |
| Chr04 | 16034305 | 16037446 | - | mRNA | Lalb_Chr04g0263681 | Putative transferase, protein kinase RLK-Pelle-LRR-III family |
| Chr04 | 16040841 | 16054397 | + | mRNA | Lalb_Chr04g0263691 | Putative ATPase, AAA-type, core, peptidase, FtsH |
| Chr04 | 16055079 | 16058361 | - | mRNA | Lalb_Chr04g0263701 | Putative soyasapogenol B glucuronide galactosyltransferase |
| Chr04 | 16059344 | 16061814 | - | mRNA | Lalb_Chr04g0263711 | Putative DnaJ domain-containing protein |
| Chr04 | 16064377 | 16071057 | + | mRNA | Lalb_Chr04g0263721 | Putative protein-serine/threonine phosphatase |
| Chr04 | 16077572 | 16079596 | - | ncRNA | Lalb_Chr04g0263731 |  |
| Chr04 | 16080864 | 16086849 | + | mRNA | Lalb_Chr04g0263741 | hypothetical protein |
| Chr04 | 16088034 | 16088986 | - | mRNA | Lalb_Chr04g0263751 | hypothetical protein |
| Chr04 | 16099019 | 16102919 | + | mRNA | Lalb_Chr04g0263761 | Putative chlorophyllase |
| Chr04 | 16103847 | 16104415 | - | mRNA | Lalb_Chr04g0263771 | hypothetical protein |
| Chr04 | 16104192 | 16104298 | + | marker | TP26007 |  |
| Chr04 | 16104417 | 16105132 | + | mRNA | Lalb_Chr04g0263781 | Putative Seed maturation protein |
| Chr04 | 16109255 | 16113205 | + | mRNA | Lalb_Chr04g0263791 | Putative transcription factor MADS-MIKC family |
| Chr04 | 16119716 | 16120105 | + | mRNA | Lalb_Chr04g0263801 | Putative carboxypeptidase D |
| Chr04 | 16121729 | 16122277 | + | mRNA | Lalb_Chr04g0263811 | Putative carboxypeptidase D |
| Chr04 | 16123607 | 16125805 | + | mRNA | Lalb_Chr04g0263821 | Putative carboxypeptidase D |
| Chr04 | 16133356 | 16136665 | + | mRNA | Lalb_Chr04g0263831 | Putative methionine N(alpha)-acetyltransferase NatB transcription regulator GNAT family |
| Chr04 | 16138316 | 16141870 | - | mRNA | Lalb_Chr04g0263841 | Putative choline transporter |
| Chr04 | 16141708 | 16141826 | - | marker | TP37593 |  |
| Chr04 | 16146117 | 16149014 | - | mRNA | Lalb_Chr04g0263851 | putative protein |
| Chr04 | 16149016 | 16153155 | - | mRNA | Lalb_Chr04g0263861 | putative protein |
| Chr04 | 16154557 | 16179182 | - | mRNA | Lalb_Chr04g0263871 | Putative structural maintenance of chromosomes protein |
| Chr04 | 16181117 | 16183326 | + | mRNA | Lalb_Chr04g0263881 | Putative RNA polymerase sigma-70 like domain-containing protein |
| Chr04 | 16184301 | 16196335 | + | mRNA | Lalb_Chr04g0263891 | Putative alpha-N-acetylglucosaminidase |
| Chr04 | 16197715 | 16198071 | - | mRNA | Lalb_Chr04g0263901 | Putative bifunctional inhibitor/plant lipid transfer protein/seed storage helical |
| Chr04 | 16200079 | 16200657 | + | mRNA | Lalb_Chr04g0263911 | hypothetical protein |
| Chr04 | 16200659 | 16209322 | + | mRNA | Lalb_Chr04g0263921 | Putative restriction endonuclease type II |
| Chr04 | 16210558 | 16211372 | - | mRNA | Lalb_Chr04g0263931 | Putative allene oxide cyclase/dirigent protein |
| Chr04 | 16212336 | 16217143 | - | mRNA | Lalb_Chr04g0263941 | Putative PMR5 domain, PC-Esterase |
| Chr04 | 16222210 | 16224717 | - | mRNA | Lalb_Chr04g0263951 | Putative xanthoxin dehydrogenase |
| Chr04 | 16234903 | 16243020 | + | mRNA | Lalb_Chr04g0263961 | Putative calcium-dependent channel, 7TM region phosphate |
| Chr04 | 16243023 | 16244922 | - | mRNA | Lalb_Chr04g0263971 | Putative leucine-rich repeat domain, L domain-containing protein |
| Chr04 | 16245209 | 16245641 | + | mRNA | Lalb_Chr04g0263981 | hypothetical protein |
| Chr04 | 16245968 | 16248087 | - | mRNA | Lalb_Chr04g0263991 | Putative TIR domain, P-loop containing nucleoside triphosphate hydrolase |
| Chr04 | 16251429 | 16253466 | - | mRNA | Lalb_Chr04g0264001 | Putative pre-rRNA-processing protein TSR2 |
| Chr04 | 16254570 | 16257142 | - | mRNA | Lalb_Chr04g0264011 | Putative annexin |
| Chr04 | 16270084 | 16272722 | + | mRNA | Lalb_Chr04g0264021 | Putative purine permease, plant |
| Chr04 | 16275830 | 16278370 | - | mRNA | Lalb_Chr04g0264031 | Putative DNA-directed RNA polymerase |
| Chr04 | 16279866 | 16284641 | + | mRNA | Lalb_Chr04g0264041 | Putative F-box domain, pectin lyase/virulence factor |
| Chr04 | 16281698 | 16281815 | + | marker | TP106254 |  |
| Chr04 | 16286029 | 16288160 | - | mRNA | Lalb_Chr04g0264051 | Putative orotate phosphoribosyltransferase, Orotidine-5'-phosphate decarboxylase |
| Chr04 | 16288300 | 16300195 | + | mRNA | Lalb_Chr04g0264061 | Putative peptidase Do |
| Chr04 | 16304569 | 16306497 | - | mRNA | Lalb_Chr04g0264071 | Putative thioredoxin-like protein |
| Chr04 | 16316356 | 16319777 | - | mRNA | Lalb_Chr04g0264081 | putative protein |
| Chr04 | 16321672 | 16330528 | + | mRNA | Lalb_Chr04g0264091 | Putative chromatin remodeling SNF2 family |
| Chr04 | 16335747 | 16340346 | - | mRNA | Lalb_Chr04g0264101 | Putative 1-phosphatidylinositol-4-phosphate 5-kinase |
| Chr04 | 16350852 | 16351025 | + | mRNA | Lalb_Chr04g0264111 | Putative knottin, scorpion toxin |
| Chr04 | 16357444 | 16361426 | - | mRNA | Lalb_Chr04g0264121 | Putative transcription factor Trihelix family |
| Chr04 | 16361588 | 16364821 | + | mRNA | Lalb_Chr04g0264131 | Putative protein-disulfide reductase |
| Chr04 | 16369696 | 16370257 | - | mRNA | Lalb_Chr04g0264141 | hypothetical protein |
| Chr04 | 16370880 | 16372390 | - | mRNA | Lalb_Chr04g0264151 | Putative transcription factor C2H2 family |
| Chr04 | 16372392 | 16373500 | - | mRNA | Lalb_Chr04g0264161 | Putative transcription factor C2H2 family |
| Chr04 | 16374881 | 16396259 | - | mRNA | Lalb_Chr04g0264171 | Putative protein kinase TKL-Pl-1 family transcription factor C2H2 family |
| Chr04 | 16397362 | 16400838 | - | mRNA | Lalb_Chr04g0264181 | hypothetical protein |
| Chr04 | 16398406 | 16398461 | - | marker | TP93026 |  |
| Chr04 | 16401291 | 16403146 | - | mRNA | Lalb_Chr04g0264191 | hypothetical protein |
| Chr04 | 16404320 | 16404622 | + | mRNA | Lalb_Chr04g0264201 | hypothetical protein |
| Chr04 | 16405347 | 16406366 | - | mRNA | Lalb_Chr04g0264211 | hypothetical protein |
| Chr04 | 16406551 | 16407023 | - | mRNA | Lalb_Chr04g0264221 | hypothetical protein |
| Chr04 | 16407025 | 16408982 | - | mRNA | Lalb_Chr04g0264231 | Putative CST complex subunit CTC1, plant protein |
| Chr04 | 16412381 | 16414218 | + | mRNA | Lalb_Chr04g0264241 | hypothetical protein |
| Chr04 | 16414574 | 16415202 | + | mRNA | Lalb_Chr04g0264251 | hypothetical protein |
| Chr04 | 16415408 | 16419956 | + | mRNA | Lalb_Chr04g0264261 | Putative ankyrin repeat-containing domain-containing protein |
| Chr04 | 16421559 | 16422293 | + | mRNA | Lalb_Chr04g0264271 | Putative rossmann-like alpha/beta/alpha sandwich protein |
| Chr04 | 16422705 | 16426291 | + | mRNA | Lalb_Chr04g0264281 | Putative protein kinase RLK-Pelle-RLCK-IXb family |
| Chr04 | 16426391 | 16428089 | + | mRNA | Lalb_Chr04g0264291 | Putative prohibitin, Band 7 domain-containing protein |
| Chr04 | 16429264 | 16433706 | + | mRNA | Lalb_Chr04g0264301 | Putative transcription factor C2H2 family |
| Chr04 | 16433708 | 16437398 | + | mRNA | Lalb_Chr04g0264311 | Putative ubiquitin system component Cue |
| Chr04 | 16437792 | 16446316 | - | mRNA | Lalb_Chr04g0264321 | Putative P-loop containing nucleoside triphosphate hydrolase |
| Chr04 | 16476635 | 16483463 | - | mRNA | Lalb_Chr04g0264331 | Putative proton-exporting ATPase |
| Chr04 | 16498546 | 16504610 | - | mRNA | Lalb_Chr04g0264341 | Putative transcription factor WD40-like family |
| Chr04 | 16504913 | 16510631 | + | mRNA | Lalb_Chr04g0264351 | Putative defective-in-cullin neddylation protein |
| Chr04 | 16511692 | 16514374 | - | mRNA | Lalb_Chr04g0264361 | Putative PPPDE putative peptidase domain-containing protein |
| Chr04 | 16519824 | 16528650 | + | mRNA | Lalb_Chr04g0264371 | Putative transcription factor C2H2 family |
| Chr04 | 16530922 | 16542328 | + | mRNA | Lalb_Chr04g0264381 | Putative XS domain-containing protein |
| Chr04 | 16543228 | 16544442 | - | mRNA | Lalb_Chr04g0264391 | hypothetical protein |
| Chr04 | 16545933 | 16548671 | - | mRNA | Lalb_Chr04g0264401 | Putative poly(A)-specific ribonuclease |
| Chr04 | 16547232 | 16547330 | + | marker | TP440375 |  |
| Chr04 | 16549593 | 16551841 | + | mRNA | Lalb_Chr04g0264411 | Putative ribosomal protein S12e |
| Chr04 | 16552197 | 16552571 | - | mRNA | Lalb_Chr04g0264421 | hypothetical protein |
| Chr04 | 16558080 | 16565337 | + | mRNA | Lalb_Chr04g0264431 | putative protein |
| Chr04 | 16575564 | 16575886 | + | mRNA | Lalb_Chr04g0264441 | Putative cinnamoyl-CoA reductase |
| Chr04 | 16577570 | 16578079 | + | mRNA | Lalb_Chr04g0264451 | Putative cinnamoyl-CoA reductase |
| Chr04 | 16580722 | 16582382 | + | mRNA | Lalb_Chr04g0264461 | Putative cinnamoyl-CoA reductase |
| Chr04 | 16597396 | 16599110 | + | mRNA | Lalb_Chr04g0264471 | Putative transcription factor WRKY family |
| Chr04 | 16599633 | 16604299 | - | mRNA | Lalb_Chr04g0264481 | Putative DNA-3-methyladenine glycosylase I |
| Chr04 | 16605558 | 16608710 | - | mRNA | Lalb_Chr04g0264491 | Putative methionine N(alpha)-acetyltransferase NatB transcription regulator GNAT family |
| Chr04 | 16614645 | 16614917 | + | ncRNA | Lalb_Chr04g0264501 |  |
| Chr04 | 16616058 | 16616539 | + | mRNA | Lalb_Chr04g0264511 | Putative O-methyltransferase COMT-type, S-adenosyl-L-methionine-dependent methyltransferase |
| Chr04 | 16627780 | 16630020 | - | mRNA | Lalb_Chr04g0264521 | Putative Type 1 galactoside alpha-(1,2)-fucosyltransferase |
| Chr04 | 16641078 | 16643100 | + | mRNA | Lalb_Chr04g0264531 | Putative Type 1 galactoside alpha-(1,2)-fucosyltransferase |
| Chr04 | 16645501 | 16650717 | + | mRNA | Lalb_Chr04g0264541 | Putative sugar phosphate transporter domain-containing protein |
| Chr04 | 16651651 | 16652808 | - | mRNA | Lalb_Chr04g0264551 | Putative F-box domain, galactose oxidase/kelch, beta-propeller |
| Chr04 | 16655912 | 16656252 | - | mRNA | Lalb_Chr04g0264561 | hypothetical protein |
| Chr04 | 16657082 | 16658583 | + | mRNA | Lalb_Chr04g0264571 | hypothetical protein |
| Chr04 | 16659234 | 16666175 | - | mRNA | Lalb_Chr04g0264581 | Putative protein-serine/threonine phosphatase |
| Chr04 | 16667359 | 16671214 | - | mRNA | Lalb_Chr04g0264591 | hypothetical protein |
| Chr04 | 16673062 | 16676533 | - | mRNA | Lalb_Chr04g0264601 | Putative protein-serine/threonine kinase CMGC-CDK-Pl family |
| Chr04 | 16680792 | 16686159 | + | mRNA | Lalb_Chr04g0264611 | hypothetical protein |
| Chr04 | 16686932 | 16689790 | - | mRNA | Lalb_Chr04g0264621 | Putative F-box domain, kelch-type beta propeller |
| Chr04 | 16691440 | 16698776 | + | mRNA | Lalb_Chr04g0264631 | Putative mannosyl-oligosaccharide 1,2-alpha-mannosidase |
| Chr04 | 16699275 | 16704369 | - | mRNA | Lalb_Chr04g0264641 | Putative cation efflux protein |
| Chr04 | 16705220 | 16707856 | - | mRNA | Lalb_Chr04g0264651 | Putative ribosomal protein L7 |
| Chr04 | 16707858 | 16722873 | + | mRNA | Lalb_Chr04g0264661 | Putative calcium-transporting ATPase |
| Chr04 | 16723528 | 16727899 | - | mRNA | Lalb_Chr04g0264681 | Putative GDP-fucose protein O-fucosyltransferase |
| Chr04 | 16724462 | 16724572 | - | marker | TP338761 |  |
| Chr04 | 16732826 | 16738225 | + | mRNA | Lalb_Chr04g0264691 | Putative adaptor protein complex AP-4, epsilon subunit |
| Chr04 | 16738558 | 16740490 | + | mRNA | Lalb_Chr04g0264701 | Putative mitochondrial glycoprotein |
| Chr04 | 16741889 | 16744832 | - | mRNA | Lalb_Chr04g0264711 | Putative Fe-S cluster assembly domain-containing protein |
| Chr04 | 16745944 | 16749236 | - | mRNA | Lalb_Chr04g0264721 | Putative FMN-binding split barrel |
| Chr04 | 16749493 | 16753658 | + | mRNA | Lalb_Chr04g0264731 | hypothetical protein |
| Chr04 | 16754504 | 16758887 | - | mRNA | Lalb_Chr04g0264741 | Putative snRNA-activating protein complex, subunit 3 |
| Chr04 | 16759739 | 16764006 | - | mRNA | Lalb_Chr04g0264751 | Putative phosphoglycerate dehydrogenase |
| Chr04 | 16765727 | 16769600 | - | mRNA | Lalb_Chr04g0264761 | Putative glucose-6-phosphate 1-epimerase |
| Chr04 | 16770536 | 16785951 | - | mRNA | Lalb_Chr04g0264771 | Putative histone acetyltransferase chromatin regulator PHD family |
| Chr04 | 16787624 | 16792338 | + | mRNA | Lalb_Chr04g0264781 | Putative 3,8-divinyl protochlorophyllide a 8-vinyl-reductase (NADPH) |
| Chr04 | 16793594 | 16795244 | + | mRNA | Lalb_Chr04g0264791 | Putative aminoacyltransferase, E1 ubiquitin-activating enzyme |
| Chr04 | 16795336 | 16797629 | + | mRNA | Lalb_Chr04g0264801 | Putative protein kinase RLK-Pelle-RLCK-IXb family |
| Chr04 | 16798927 | 16805936 | + | mRNA | Lalb_Chr04g0264811 | Putative inorganic diphosphatase |
| Chr04 | 16806710 | 16808933 | + | mRNA | Lalb_Chr04g0264821 | Putative small auxin-up RNA |
| Chr04 | 16809638 | 16812954 | + | mRNA | Lalb_Chr04g0264831 | Putative mitochondrial carrier protein |
| Chr04 | 16813672 | 16817369 | - | mRNA | Lalb_Chr04g0264841 | Putative ribosomal protein L34Ae |
| Chr04 | 16818373 | 16822407 | - | mRNA | Lalb_Chr04g0264851 | Putative plastid lipid-associated protein/fibrillin |
| Chr04 | 16823618 | 16823923 | - | ncRNA | Lalb_Chr04g0264861 |  |

# Supplementary Table S13. Kinase, coiled-coil (CC), leucine-rich repeat (LRR), nucleotide binding site (NBS), Toll/interleukin-1 receptor (TIR) and transmembrane (TM) domains identified in genes localized in the regions of white lupin genome carrying anthracnose resistance loci.

| **Locus** | **Kinase** | **CC** | **LRR** | **NBS** | **TIR** | **TM** | **EC number** |
| --- | --- | --- | --- | --- | --- | --- | --- |
| Lalb_Chr02g0140541 |  |  | + |  |  | + | 2.7.11.1 |
| Lalb_Chr02g0140731 | + |  | + |  |  | + | 2.7.11.25 |
| Lalb_Chr02g0140831 | + | + |  |  |  | + | 2.7.11.1 |
| Lalb_Chr02g0140841 | + |  |  |  |  | + | 2.7.10.2 |
| Lalb_Chr02g0140951 | + |  | + |  |  | + | 2.7.11.1 |
| Lalb_Chr02g0141221 | + |  |  |  |  | + | 2.7.11.1 |
| Lalb_Chr02g0141291 |  |  |  | + |  | + |  |
| Lalb_Chr02g0141511 | + |  |  |  |  | + | 2.7.11.1 |
| Lalb_Chr02g0141521 | + |  |  |  |  | + | 2.7.11.- |
| Lalb_Chr02g0141611 | + |  | + |  |  | + | 2.7.11.1 |
| Lalb_Chr02g0141701 | + |  | + |  |  | + | 2.7.-.- |
| Lalb_Chr02g0141771 |  |  |  | + |  | + | 3.6.3.21 |
| Lalb_Chr02g0141801 | + |  |  |  |  | + | 2.7.11.1 |
| Lalb_Chr02g0142341 |  |  |  | + |  |  |  |
| Lalb_Chr04g0263281 | + |  |  |  |  |  | 2.7.11.1 |
| Lalb_Chr04g0263521 | + |  |  |  |  | + | 2.7.11.1 |
| Lalb_Chr04g0263561 | + |  |  |  |  | + | 2.7.11.1 |
| Lalb_Chr04g0263681 | + |  | + |  |  | + | 2.7.-.- |
| Lalb_Chr04g0263971 |  |  | + |  |  |  |  |
| Lalb_Chr04g0263991 |  |  |  | + | + | + |  |
| Lalb_Chr04g0264171 | + |  |  |  |  | + | 2.7.11.1 |
| Lalb_Chr04g0264281 | + | + |  |  |  | + | 2.7.11.1 |
| Lalb_Chr04g0264601 | + |  |  |  |  |  | 2.7.11.- |
| Lalb_Chr04g0264651 |  | + |  |  |  |  |  |
| Lalb_Chr04g0264801 | + | + |  |  |  | + | 2.7.11.1 |

**
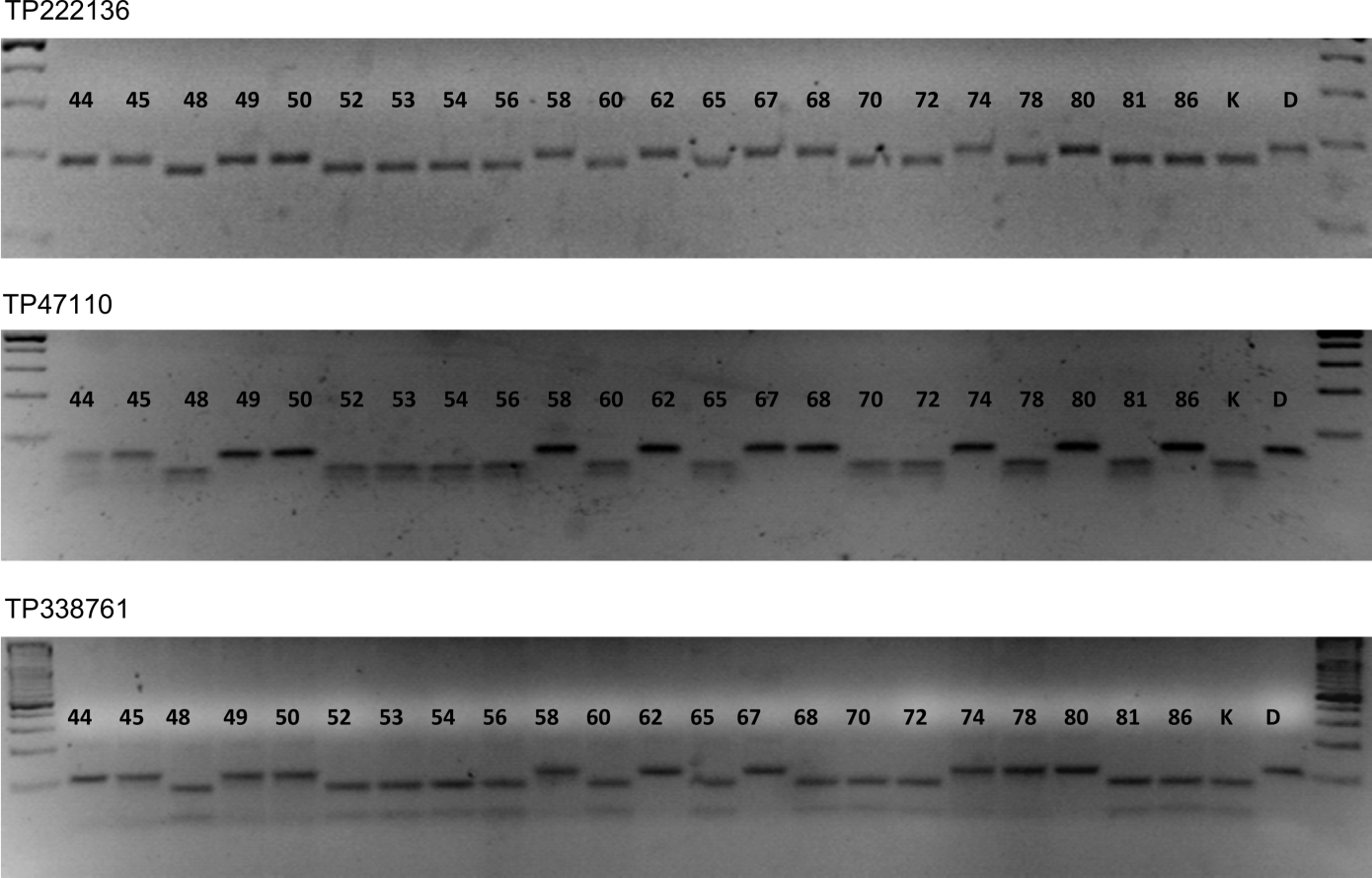
**

# Supplementary Figure S1. Agarose gel electrophoresis of positively validated PCR-based markers for two major anthracnose resistance QTL loci, antr04_1/antr05_1 (TP222136 and TP47110) and antr04_2/antr05_2 (TP338761).

44-86, recombinant inbred lines of K × D mapping population; K, Kiev Mutant; D, line P27174
